# Impact of population aging on future temperature-related mortality at different global warming levels

**DOI:** 10.1038/s41467-024-45901-z

**Published:** 2024-02-27

**Authors:** Kai Chen, Evan de Schrijver, Sidharth Sivaraj, Francesco Sera, Noah Scovronick, Leiwen Jiang, Dominic Roye, Eric Lavigne, Jan Kyselý, Aleš Urban, Alexandra Schneider, Veronika Huber, Joana Madureira, Malcolm N. Mistry, Ivana Cvijanovic, MCC Collaborative Research Network, Ben Armstrong, Rochelle Schneider, Aurelio Tobias, Christofer Astrom, Yuming Guo, Yasushi Honda, Rosana Abrutzky, Shilu Tong, Micheline de Sousa Zanotti Stagliorio Coelho, Paulo Hilario Nascimento Saldiva, Patricia Matus Correa, Nicolás Valdés Ortega, Haidong Kan, Samuel Osorio, Hans Orru, Ene Indermitte, Jouni J. K. Jaakkola, Niilo Ryti, Mathilde Pascal, Klea Katsouyanni, Antonis Analitis, Fatemeh Mayvaneh, Alireza Entezari, Patrick Goodman, Ariana Zeka, Paola Michelozzi, Francesca de’Donato, Masahiro Hashizume, Barrak Alahmad, Magali Hurtado Diaz, César De la Cruz Valencia, Ala Overcenco, Danny Houthuijs, Caroline Ameling, Shilpa Rao, Gabriel Carrasco-Escobar, Xerxes Seposo, Susana Pereira da Silva, Iulian Horia Holobaca, Fiorella Acquaotta, Ho Kim, Whanhee Lee, Carmen Íñiguez, Bertil Forsberg, Martina S. Ragettli, Yue-Liang Leon Guo, Shih-Chun Pan, Shanshan Li, Valentina Colistro, Antonella Zanobetti, Joel Schwartz, Tran Ngoc Dang, Do Van Dung, Hanne Krage Carlsen, John Paul Cauchi, Souzana Achilleos, Raanan Raz, Antonio Gasparrini, Ana M. Vicedo-Cabrera

**Affiliations:** 1 Department of Environmental Health Sciences, Yale School of Public Health, New Haven, CT USA; 2 Yale Center on Climate Change and Health, Yale School of Public Health, New Haven, CT USA; 3 Institute of Social and Preventive Medicine, University of Bern, Bern, Switzerland; 4 Oeschger Center for Climate Change Research, University of Bern, Bern, Switzerland; 5 Department of Statistics, Computer Science and Applications “G. Parenti”, University of Florence, Florence, Italy; 6 Gangarosa Department of Environmental Health. Rollins School of Public Health, Emory University, Atlanta, GA USA; 7 Asian Demographic Research Institute, Shanghai University, Shanghai, China; 8 Population Council, New York, NY USA; 9 Climate Research Foundation (FIC), Madrid, Spain; 10 CIBER de Epidemiología y Salud Pública (CIBERESP), Madrid, Spain; 11 Environmental Health Science and Research Bureau, Health Canada, Ottawa, ON Canada; 12 School of Epidemiology and Public Health, University of Ottawa, Ottawa, ON Canada; 13 Institute of Atmospheric Physics, Czech Academy of Sciences, Prague, Czech Republic; 14 Faculty of Environmental Sciences, Czech University of Life Sciences, Prague, Czech Republic; 15 Institute of Epidemiology, Helmholtz Zentrum München – German Research Center for Environmental Health (GmbH), Neuherberg, Germany; 16 Chair of Epidemiology, Faculty of Medicine, LMU Munich, Munich, Germany; 17 Department of Enviromental Health, Instituto Nacional de Saúde Dr Ricardo Jorge, Porto, Portugal; 18 EPIUnit – Instituto de Saúde Pública, Universidade do Porto, Porto, Portugal; 19 Laboratório para a Investigação Integrativa e Translacional em Saúde Populacional (ITR), Porto, Portugal; 20 Environment & Health Modelling (EHM) Lab, Department of Public Health, Environments and Society, London School of Hygiene & Tropical Medicine, London, United Kingdom; 21 Department of Economics, Ca’ Foscari University of Venice, Venice, Italy; 22 ISGlobal - Barcelona Institute for Global Health, Barcelona, Spain; 23 Department of Public Health, Environments and Society, London School of Hygiene & Tropical Medicine, London, United Kingdom; 24 Φ-Lab, European Space Agency, Frascati, Italy; 25 Forecast Department, European Centre for Medium-Range Weather Forecast (ECMWF), Reading, UK; 26 Institute of Environmental Assessment and Water Research, Spanish Council for Scientific Research, Barcelona, Spain; 27 School of Tropical Medicine and Global Health, Nagasaki University, Nagasaki, Japan; 28 Department of Public Health and Clinical Medicine, Umeå University, Umeå, Sweden; 29 Climate, Air Quality Research Unit, School of Public Health and Preventive Medicine, Monash University, Melbourne, VIC Australia; 30 Faculty of Health and Sport Sciences, University of Tsukuba, Tsukuba, Japan; 31 Universidad de Buenos Aires, Facultad de Ciencias Sociales, Instituto de Investigaciones Gino Germani, Buenos Aires, Argentina; 32 National Institute of Environmental Health, Chinese Center for Disease Control and Prevention, Beijing, China; 33 School of Public Health and Social Work, Queensland University of Technology, Brisbane, QLD Australia; 34 Urban Health Laboratory, University of São Paulo, Faculty of Medicine, São Paulo, Brazil; 35 Centro Interdisciplinario de Cambio Global, Pontificia, Universidad Católica de Chile, Santiago, Chile; 36 Shanghai Key Laboratory of Atmospheric Particle Pollution and Prevention (LAP3), Fudan University, Shanghai, 200030 China; 37 Department of Environmental Health, University of São Paulo, São Paulo, Brazil; 38 Institute of Family Medicine and Public Health, University of Tartu, Tartu, Estonia; 39 Center for Environmental and Respiratory Health Research (CERH), University of Oulu, Oulu, Finland; 40 Finnish Meteorological Institute, Helsinki, Finland; 41 Santé Publique France, Department of Environmental Health, French National Public Health Agency, Saint Maurice, France; 42 Department of Hygiene, Epidemiology and Medical Statistics, National and Kapodistrian University of Athens, Athens, Greece; 43 Environmental Research Group, School of Public Health, Imperial College London, London, UK; 44 Faculty of Geography and Environmental Sciences, Hakim Sabzevari University, Sabzevar Khorasan Razavi, Iran; 45 Technological University Dublin, Dublin, Ireland; 46 Institute for the Environment, Brunel University London, London, UK; 47 Department of Epidemiology, Lazio Regional Health Service, ASL Roma 1, Rome, Italy; 48 Department of Global Health Policy, School of International Health, Graduate School of Medicine, The University of Tokyo, Tokyo, Japan; 49 Department of Environmental Health, Harvard T.H. Chan School of Public Health, Harvard University, Boston, MA USA; 50 Department of Environmental Health, National Institute of Public Health, Cuernavaca Morelos, Mexico; 51 Laboratory of Management in Science and Public Health, National Agency for Public Health of the Ministry of Health, Chisinau, Republic of Moldova; 52 National Institute for Public Health and the Environment (RIVM), Centre for Sustainability and Environmental Health, Bilthoven, Netherlands; 53 Norwegian Institute of Public Health, Oslo, Norway; 54 Institute of Tropical Medicine “Alexander von Humboldt”, Universidad Peruana Cayetano Heredia, Lima, Peru; 55 Department of Environmental Engineering, Graduate School of Engineering, Kyoto University, Kyoto, Japan; 56 Department of Epidemiology, Instituto Nacional de Saúde Dr Ricardo Jorge, Lisboa, Portugal; 57 Faculty of Geography, Babes-Bolay University, Babes-Bolay, Romania; 58 Department of Earth Sciences, University of Torino, Turin, Italy; 59 Graduate School of Public Health & Institute of Health and Environment, Seoul National University, Seoul, Republic of Korea; 60 School of Biomedical Convergence Engineering, Pusan National University, Yangsan, South Korea; 61 Department of Statistics and Computational Research, Universitat de València, València, Spain; 62 Ciberesp, Madrid, Spain; 63 Swiss Tropical and Public Health Institute, Allschwil, Switzerland; 64 University of Basel, Basel, Switzerland; 65 Environmental and Occupational Medicine, and Institute of Environmental and Occupational Health Sciences, National Taiwan University (NTU) and NTU Hospital, Taipei, Taiwan; 66 National Institute of Environmental Health Science, National Health Research Institutes, Zhunan, Taiwan; 67 Department of Quantitative Methods, School of Medicine, University of the Republic, Montevideo, Uruguay; 68 Department of Environmetal Health, Faculty of Public Health, University of Medicine and Pharmacy at Ho Chi Minh City, Ho Chi Minh City, Vietnam; 69 School of Public Health and Community Medicine, University of Gothenburg, Gothenburg, Sweden; 70 Infectious Disease Prevention and Control Unit (IDCU), Health Promotion and Disease Prevention, Msida, Malta; 71 Department of Primary Care and Population Health, University of Nicosia Medical School, Nicosia, Cyprus; 72 Braun School of Public Health and Community Medicine, The Hebrew University of Jerusalem, Jerusalem, Israel

**Keywords:** Risk factors, Environmental impact

## Abstract

Older adults are generally amongst the most vulnerable to heat and cold. While temperature-related health impacts are projected to increase with global warming, the influence of population aging on these trends remains unclear. Here we show that at 1.5 °C, 2 °C, and 3 °C of global warming, heat-related mortality in 800 locations across 50 countries/areas will increase by 0.5%, 1.0%, and 2.5%, respectively; among which 1 in 5 to 1 in 4 heat-related deaths can be attributed to population aging. Despite a projected decrease in cold-related mortality due to progressive warming alone, population aging will mostly counteract this trend, leading to a net increase in cold-related mortality by 0.1%–0.4% at 1.5–3 °C global warming. Our findings indicate that population aging constitutes a crucial driver for future heat- and cold-related deaths, with increasing mortality burden for both heat and cold due to the aging population.

## Abstract (web summary)

This study reveals that population aging intensifies heat- and cold-related deaths, more so than climate change, in 50 countries. At 1.53 °C global warming, aging contributes to rising heat-related deaths, offsetting declines in cold related death.

## Introduction

Climate change poses profound public health threats for current and future generations^1,2^ Among the many pathways by which climate change affects human health, heat has the most immediate and direct impact^2^. The world set a new warming record in 2022 reaching 1.2 °C of global temperature increase above the pre-industrial levels (1850–1900)^3^. Between 1991–2018, 37% of the warm-season heat-related mortality burden could be attributed to recent human-induced climate change^4^. In addition to heat, cold is also associated with increased morbidity and mortality^5,6^. According to a recent global analysis, non-optimal temperatures (i.e., both heat and cold, considering the whole range of observed values) account for more than 5 million deaths globally every year (2000–2019), with the burden attributable to cold 9 times greater than that attributable to heat^7^. Current projections indicate that heat-related deaths will steeply increase under warmer climates, while cold-related deaths will decline in most locations given the reduction in cold days^8,9^. However, these studies do not account for an aging population. Current estimates of future mortality burden mostly account only for changes in climate, while there is limited quantitative evidence on future health impacts of climate change under more complex and potentially realistic scenarios. In this respect, understanding the influence of other global changes in addition to the changing climate, on the change in temperature (heat and cold)-related mortality at different levels of warming vs. current times remains a critical issue in adequately quantifying the overall health impact of climate change.

While the climate is changing, the world is facing another public health challenge: aging. The proportion of world’s population aged 65 years and above is projected to rise from 9% at present to 16% in 2050^10^. Older adults are considered among the most vulnerable populations to non-optimal temperatures^11^, due to factors such as more limited thermoregulatory responses, relatively high prevalence of chronic conditions, and a higher likelihood of social isolation^12,13^. Understanding the influence of different population demographic scenarios on the estimated temperature-related health impacts can provide important insights on plausible future health burdens. Given the increased risk present amongst older adults, it is expected that population aging will substantially amplify the future temperature-related impacts by increasing the vulnerability of the populations^14^. A recent review of local and regional temperature-related health impacts found that accounting for population aging could lead to a lower reduction or even an increase in the cold-related mortality burden, resulting in substantial increases in temperature-related mortality^14^. A recent nation-wide assessment in Switzerland found an increasing trend in the projected cold-related mortality under warmer climates due to an increase in the size of the population at risk when demographic changes were accounted for^1^. However, evidence on the global scale using a systematic and standardized analytical approach is limited. No study before has provided explicit estimates of the contribution of aging in future climate warming scenarios.

**Fig. 1:**
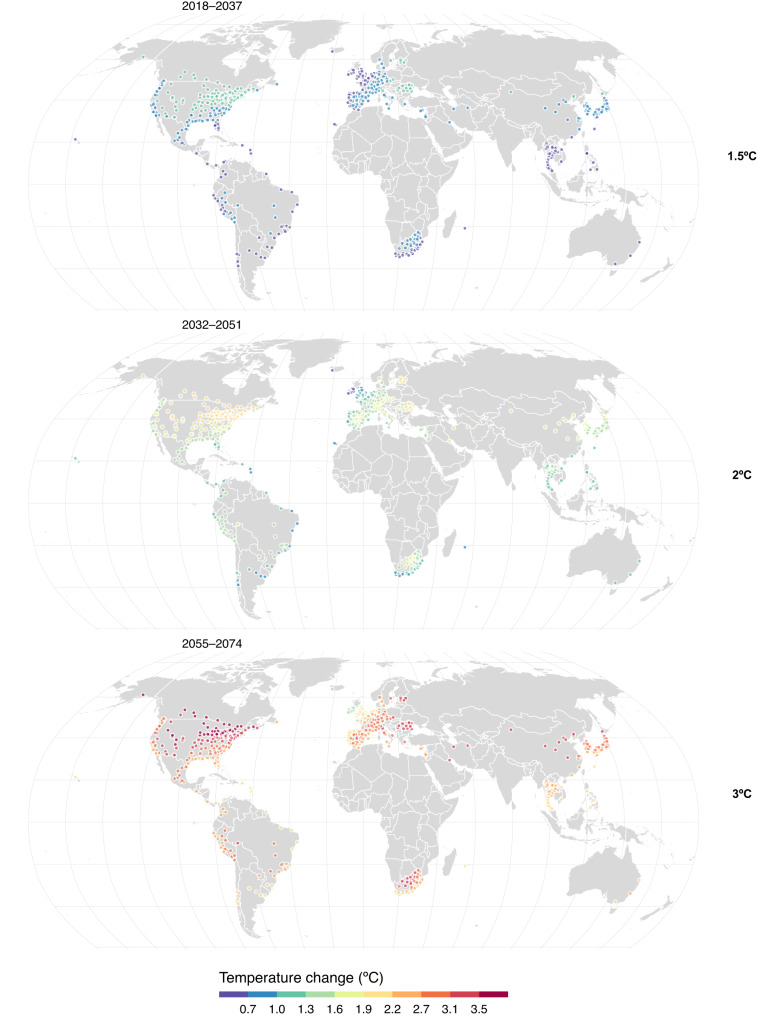
Projections of average daily mean temperature changes under global warming levels. Distribution of the 800 locations with projected temperature changes at 1.5 °C, 2 °C, and 3 °C) of global warming. The future periods in which the 20-year running mean of global mean temperature first reaches the 1.5 °C, 2 °C, and 3 °C of warming above pre-industrial level (1850–1900) are 2018–2037, 2032–2051, and 2055–2074, respectively under SSP5-8.5. The administrative map data is from the giscoR package (https://ropengov.github.io/giscoR/).

In this work, we assess the impact of population aging on future temperature-related excess mortality at different levels of global warming (1.5 °C: 2018–2037, 2 °C: 2032–2051, and 3 °C: 2055–2074) in 800 locations across 50 countries/areas (Fig. 1). In brief, we first estimate the location-age-specific temperature-mortality associations in a two-stage time-series analysis using quasi-Poisson regression with distributed lag nonlinear models and multivariate random meta-regression using data from the Multi-Country Multi-City (MCC) Collaborative Research Network (http://mccstudy.lshtm.ac.uk/) (description provided in Supplementary Tables 1 and 2). We then combine the association estimates with bias-corrected future temperature series from 18 general circulation models (GCMs) (Coupled Model Inter-comparison Project 6 - CMIP6)^15^, and future trends in age-specific baseline mortality^2^ to derive excess temperature-mortality projections under three warming scenarios (1.5 °C, 2 °C, and 3 °C of global warming above preindustrial level) and for the historical period (1995–2014). Finally, we quantify the impact of population aging as the difference in the change in temperature-related mortality fractions (warming window minus historical period) between climate-only (i.e., not accounting for changes in population demographics) and climate-population scenarios. Mortality fractions refer to the percentage of all-cause deaths attributed to heat and cold over total mortality in each time window.

## Results

### Projected temperature changes at different global warming levels

We used the temperature projections under the very high greenhouse gas (GHG) emission scenario – the Shared Socio-economic Pathway 5-8.5 (SSP5-8.5) and identified temperature windows consistent with such rises in global mean surface temperature^16^. To explore the influence of the timing of the temperature windows on population aging, we also conducted a sensitivity analysis using the emission scenario SSP3-7.0 (i.e., 1.5 °C: 2021–2040, 2 °C: 2037–2056, and 3 °C: 2066–2085), under which a 3 °C warming will be also crossed but under different trajectories of warming and population structure changes^16^. The ‘regional rivalry’ SSP3 scenario is a socio-political scenario envisioning a resurgent nationalism, concerns about competitiveness and security, and regional conflicts that result in high challenges for both mitigation and adaptation^17^. Supplementary Fig. 1A shows the multi-model ensemble-average and range of global mean surface temperature changes relative to 1995–2014 (defined as present-day in the IPCC Sixth Assessment Report) under SSP5-8.5 and SSP3-7.0 scenarios from 18 GCMs (Supplementary Table 3). Trends in global mean surface temperature changes are similar under both emission scenarios before 2040 and begin to slowly diverge after 2040. By the end of the 21^st^ century (2081-2100), the ensemble average of 18 GCMs suggests global surface temperature changes increase by 4.6 °C and 3.7 °C under SSP5-8.5 and SSP3-7.0, respectively, relative to our historical baseline (1995–2014). We further bias-corrected the daily temperature projections at each MCC location, using the historical temperature observations and a method described previously^3,4^. Figure 1 illustrates the average temperature changes in 800 locations at 1.5 °C (2018-2037), 2 °C (2032-2051), and 3 °C (2055-2074) warming levels under the SSP5-8.5. For these time windows, temperatures in the 800 studied locations increase by 0.8 °C (range of 0.3 °C to 1.3 °C), 1.5 °C (0.7 °C to 2.2 °C), and 2.7 °C (1.3 °C to 4.2 °C) compared to the historical period (1995–2014) (Supplementary Fig. 1B). With increasing levels of global warming, larger variability can be observed in temperature changes across the locations. The largest temperature increases mostly occur in locations in Northern America (e.g., Winnipeg, Manitoba, Canada; and Fargo, North Dakota, USA) and Northern Europe (e.g., Narva linn, Estonia), with an average historical temperature below 13.8 °C. Almost identical temperature increases occur at the studied locations under SSP3-7.0, with an average of 0.8 °C (0.4 °C to 1.3 °C), 1.5 °C (0.7 °C to 2.2 °C), and 2.7 °C (1.3 °C to 4.3 °C) increase compared with the historical period at the 1.5 °C (2021–2040), 2 °C (2037–2056), and 3 °C (2066–2085) warming levels, respectively.

### Population aging at different global warming levels

**Fig. 2:**
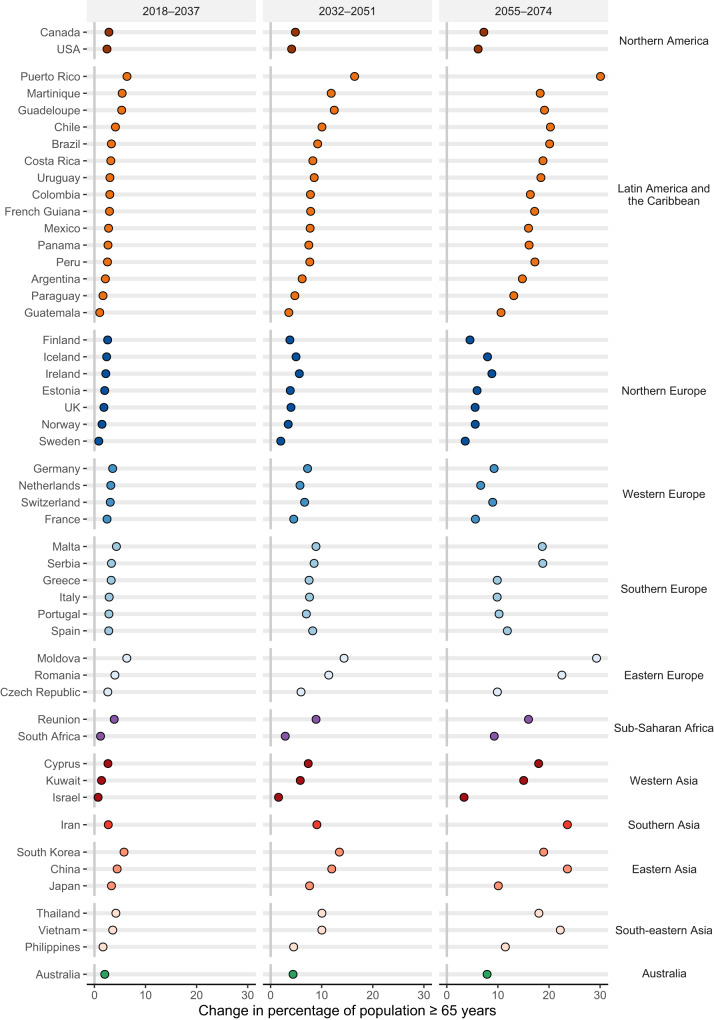
Population aging at 20-year periods corresponding to different levels (1.5 °C, 2 °C, and 3 °C - SSP5-8.5) of global warming by country/area. Country/area-specific age-group population projections are derived from the SSP5 scenario in the first 20-year periods reaching 1.5 °C (2018–2037), 2 °C (2032–2051), and 3 °C (2055–2074) of warming, respectively. X-axis shows the changes in percentage of population ≥ 65 years (%).

We estimated population aging as the change in the percentage of the population of 65 years and above under SSP5^5^. in each country during the 20-year period reaching 1.5 °C (2018-2037), 2 °C (2032–2051), and 3 °C (2055–2074) global warming relative to the historical period (Fig. 2). Across the 50 countries/areas, the average percentage of the population 65 years and above is projected to increase by 3.0%, 7.3%, and 13.8% when global warming reaches 1.5 °C, 2 °C, and 3 °C, respectively. In our study countries/areas, at 3 °C of warming (2055-2074), countries/areas in Southern Asia experience the largest increase in population aging (23.6%), followed by the countries/areas in Eastern Europe (20.1%), Latin America and the Caribbean (17.8%), Eastern Asia (17.50%), South-eastern Asia (17.2%); whereas countries/areas in North Europe (6.0%) have the lowest rate of population aging, followed by Northern America (6.7%), Western Europe (7.6%), and Australia (7.9%). In the sensitivity analysis under SSP3, a similar increasing trend in the average percentage of the population 65 years and above is expected at 1.5 °C (3.6%), 2 °C (6.4%), and 3 °C (9.1%) of warming levels, respectively (Supplementary Fig. 2).

### Changes in temperature-related mortality burden at different warming levels

We derived heat- and cold-related mortality estimates assuming: 1) constant demographic structure and size of the population consistent with the year 2015 (climate-only), and 2) time-varying population demographic structure and size according to SSP5 (fossil-fueled development with high challenge for mitigation and low challenge for adaptation) population scenario (and SSP3 population scenario in the sensitivity analysis)^18^. We derived age group-specific baseline mortality projections during each temperature window according to the selected SSP based on projected population size and mortality rates, and used the age-specific association estimates (description provided in Methods and Supplementary Text) to derive the corresponding heat- and cold-related mortality. We assume no changes in population adaptation across time (i.e., constant exposure-response association).

In the climate-population scenario, we calculated the heat- and cold-related mortality fractions, and total (i.e. non-optimal temperatures), and their changes in the future period relative to the historical period by applying the location and age group-specific associations (Supplementary Fig. 3) to the corresponding daily mean temperature and baseline mortality series in each 20-year period. For the “climate-only” scenario, the same methodology was used but assuming a constant baseline mortality corresponding to the average mortality in each day of the year of the historical period. Because using SSP5-8.5 and SSP3-7.0 scenarios yielded similar or even identical results on the temperature-related mortality burden estimates (Table 1 and Supplementary Fig. 4), we focus on presenting the main results from the SSP5-8.5 scenario hereafter.

**Table 1:** Changes in temperature-related excess mortality fractions (%) at different levels of global warming vs. historical period under the climate-population and climate-only scenarios

Scenario	Warming level	Cold	Heat	Non-optimal temperatures
SSP5-8.5				
Climate-only scenario	1.5 °C warming	−0.60 (−0.97 −0.29)	0.38 (0.02, 0.85)	−0.22 (−0.65, 0.12)
	2 °C warming	−1.03 (−1.56, −0.59)	0.77 (−0.01, 1.69)	−0.25 (−1.10, 0.50)
	3 °C warming	−1.75 (−2.65, −1.05)	1.83 (−0.47, 4.46)	0.08 (−2.35, 2.31)
Climate-population scenario	1.5 °C warming	0.08 (−0.22, 0.39)	0.46 (0.03, 1.00)	0.54 (0.06, 0.90)
	2 °C warming	0.43 (0.01, 0.83)	1.04 (−0.04, 2.18)	1.48 (0.33, 2.38)
	3 °C warming	0.17 (−0.70, 0.76)	2.50 (−1.25, 5.94)	2.67 (−1.28, 5.62)
SSP3-7.0				
Climate-only scenario	1.5 °C warming	−0.62 (−1.10, −0.32)	0.39 (0.02, 0.94)	−0.23 (−0.69, 0.13)
	2 °C warming	−1.02 (−1.65, −0.60)	0.77 (−0.03, 1.82)	−0.25 (−1.12, 0.53)
	3 °C warming	−1.74 (−2.62, −1.04)	1.81 (−0.54, 4.44)	0.07 (−2.38, 2.31)
Climate-population scenario	1.5 °C warming	0.67 (0.24, 0.99)	0.53 (0.04, 1.17)	1.20 (0.64, 1.67)
	2 °C warming	1.05 (0.47, 1.50)	1.11 (−0.02, 2.39)	2.16 (0.92, 3.20)
	3 °C warming	0.33 (−0.45, 0.85)	2.56 (−1.32, 6.09)	2.89 (−1.18, 6.02)

Estimates correspond to the change in temperature-related mortality fractions (heat and cold) at different levels of global warming under SSP5-8.5 and SSP3-7.0 scenarios compared with the historical period (1995–2014). Heat is defined as all temperatures above the minimum mortality temperature (MMT) and cold is defined as all temperatures below the MMT (Supplementary Fig. 3). The future periods in which the 20-year running mean of global mean temperature first reaches the 1.5 °C, 2 °C, and 3 °C of warming above pre-industrial level (1850–1900) are 2018–2037, 2032–2051, and 2055–2074, respectively under SSP5-8.5; and 2021–2040, 2037–2056, 2066–2085, respectively under SSP3-7.0.

**Fig. 3:**
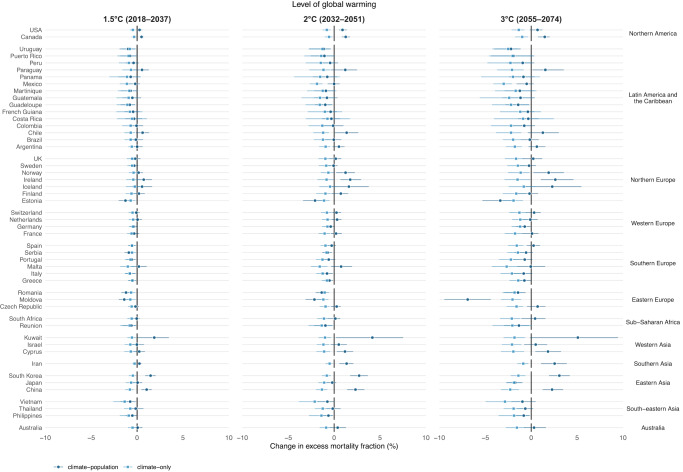
Changes in cold-related excess mortality fractions (%) at different levels of global warming by country/area under the climate-population and climate-only scenarios, compared with the historical period (1995–2014). Estimates (shown as dots) are reported as the ensemble average of 18 general circulation models under the SSP5-8.5 scenario. The whiskers represent the 95% empirical confidence intervals. The future periods in which the 20-year running mean of global mean temperature first reaches the 1.5 °C, 2 °C, and 3 °C of warming above pre-industrial level (1850–1900) are 2018–2037, 2032–2051, and 2055–2074, respectively under SSP5-8.5.

**Fig. 4:**
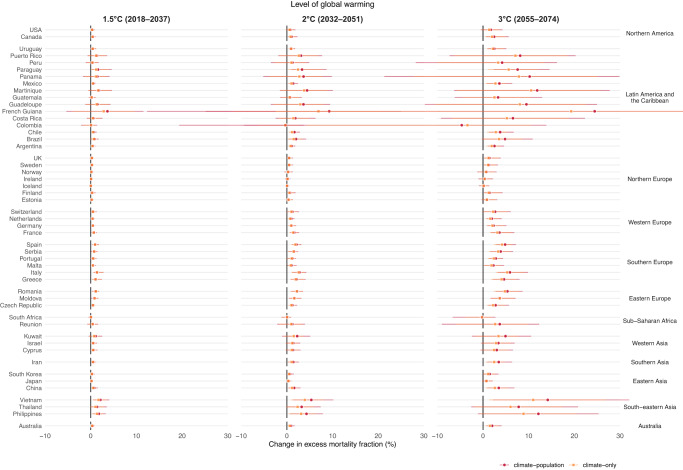
Changes in heat-related excess mortality fractions (%) at different levels of global warming by country/area under the climate-population and climate-only scenarios, compared with the historical period (1995–2014). Estimates (shown as dots) are reported as the ensemble average of 18 general circulation models under the SSP5-8.5 scenario. The whiskers represent the 95% empirical confidence intervals. The future periods in which the 20-year running mean of global mean temperature first reaches the 1.5 °C, 2 °C, and 3 °C of warming above pre-industrial level (1850–1900) are 2018–2037, 2032–2051, and 2055–2074, respectively under SSP5-8.5.

Under the climate-only scenario, heat-related mortality fractions increased but cold-related mortality fractions decreased overall, resulting in a small decrease in mortality burden due to non-optimal temperatures at 1.5 °C warming (−0.2% [empirical confidence interval (eCI) −0.7% to 0.1%)] and 2 °C [−0.3% (95% eCI: −1.1% to 0.5%)] (Table 1; country-specific results are shown in Figs. 3 and 4, and Supplementary Table 4), relative to the historical period. However, under 3 °C of global warming, an overall small increase in temperature-related mortality burden [0.1% (95% eCI: −2.4% to 2.3%)] can be observed due to larger increases in heat-related mortality than the decreases in cold-related mortality.

Conversely, under the climate-population scenario, heat-related mortality fractions increased more substantially along with increasing levels of global warming up to 2.5% (95% eCI: −1.3% to 5.9%) at 3 °C warming (Table 1 and Supplementary Table 5). More importantly, an increase in cold-related mortality fractions from 0.1% (95% eCI: −0.2% to 0.4%) at 1.5 °C warming to 0.2% (95% eCI: −0.7% to 0.8%) at 3 °C scenario was found. This resulted in an overall increase in mortality due to non-optimal temperature at all warming levels up to 2.7% (95% eCI: −1.3% to 5.6%) in the 3 °C scenario.

The largest increase in non-optimal (cold and heat combined) temperature-related mortality is observed in Vietnam [13.2% (95% eCI: 2.4% to 30.4%] and Kuwait [10.0% (95% eCI: 0.1% to 17.7%)], also French Guiana[24.1% (95% eCI: −74.0% to 60.7%), Philippines [11.3% (95% eCI: −1.8%, 24.3%)], and Martinique [10.6% (95% eCI: −7.3% to 26.2%)] despite the highly imprecise estimates (Supplementary Table 6). Among these increases, heat-related mortality played the dominant role in Vietnam (−1.0% for cold and 14.2% for heat), French Guiana (−0.4% for cold and 24.5% for heat), Philippines (−0.8% for cold and 12.1% for heat), and Martinique (−1.2% for cold and 11.8% for heat), whereas heat-related mortality played a smaller role than cold-related mortality in Kuwait (5.1% for cold and 4.9% for heat). The decreases in the non-optimal temperature-related mortality are found in Colombia [−5.4% (95% eCI: −67.9% to 13.3%)], followed by Moldova [−3.2% (95% eCI: −5.9% to −0.3%)], Estonia [−2.5% (95% eCI: −4.4% to −0.8%)], and Japan [-0.9% (95% eCI: −2.2% to 0.1%)]. The decreases in these countries are mainly due to the small increases in heat-related mortality. In Colombia, due to the slightly decreased but non-significant heat-related mortality risks (Supplementary Fig. 3), decreases are observed for both cold- and heat-related mortality [−0.8% (95% eCI: −2.1% to 0.4%) for cold and −4.7% (95% eCI: −66.5% to 13.9%)], albeit both statistically insignificant (Figs. 3 and 4). Results for heat and cold separately are provided in the next section.

### Impact of population aging on future temperature-related mortality

**Fig. 5:**
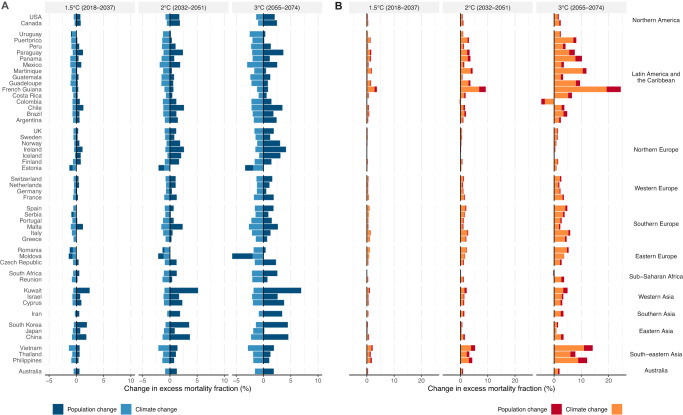
Contributions of climate change and population change to the changes in cold- and heat-related mortality at different levels of global warming. **A** change in cold-related excess mortality fraction (%); **B** change in heat-related excess mortality fraction (%). Country/area-level changes by climate change and population aging are shown at 1.5 °C, 2 °C, and 3 °C of global warming using a 20-year window compared with the historical period 1995–2014 under SSP5-8.5. The future periods in which the 20-year running mean of global mean temperature first reaches the 1.5 °C, 2 °C, and 3 °C of warming above pre-industrial level (1850–1900) are 2018–2037, 2032–2051, and 2055–2074, respectively under SSP5-8.5. The impact of population aging was estimated by subtracting the future changes in temperature-related impacts in the constant population scenario (“climate-only”) from the changes in temperature-related impacts under the SSP5 mortality projection in the “climate-population” scenario. Note the different scales in the x-axis used for heat and cold.

Figure 5 compares the changes in cold- and heat-related excess mortality driven by climate change and population aging at different levels of global warming relative to the historical period (1995–2014) under SSP5-8.5. The climate change component corresponds to the change in temperature- (cold or heat) related mortality fractions in the climate-only scenario, while population aging is calculated from the difference between the change in temperature- (cold or heat) related mortality fractions estimated in climate-population scenario and the climate-only scenario (i.e., a constant population scenario). At 1.5 °C warming, all 50 countries/areas will experience a reduction (−1.4% in Vietnam to −0.3% in Iceland) in cold-related mortality burden driven by climate change alone (Fig. 5A). However, population aging will mostly offset this decrease and result in an increased cold-related mortality burden in 18 out of the 50 countries/areas (0.0% in Argentina to 1.9% in Kuwait). At higher levels of warming, the decline in cold-related mortality burden due to climate change will be further offset by a larger increase due to population aging, leading to 23 and 21 countries/areas having an increase in cold-related mortality burden under 2 and 3 °C warming, respectively.

For heat-related mortality (Fig. 5B), climate change alone will result in increased mortality burden in all countries/areas at 1.5 °C warming (0.02% in Iceland to 2.8% in French Guiana), 49 countries/areas (0.03% in South Africa to 6.9% in French Guiana) except for Colombia (−0.3%) at 2 °C warming, and 48 countries/areas (0.1% in Iceland to 19.3% in French Guiana) except for Colombia [−3.4% (95% eCI: −38.6% to 10.5%)] and South Africa [−0.2% (95% eCI: −3.9% to 2.0%)] at 3 °C warming. The decreases seen in Colombia and South Africa are due to the decreased but non-significant heat-related mortality risks (Supplementary Fig. 3). Compared with climate change, population aging will have a smaller contribution to the changes in heat-related mortality, leading to an increase of 0.1–0.7% at 1.5–3 °C warming across 50 countries/areas. This translates into 1 in 5 to 1 in 4 deaths in the 0.5–2.5% increase in heat-related deaths attributable to aging (Table 1).

**Fig. 6:**
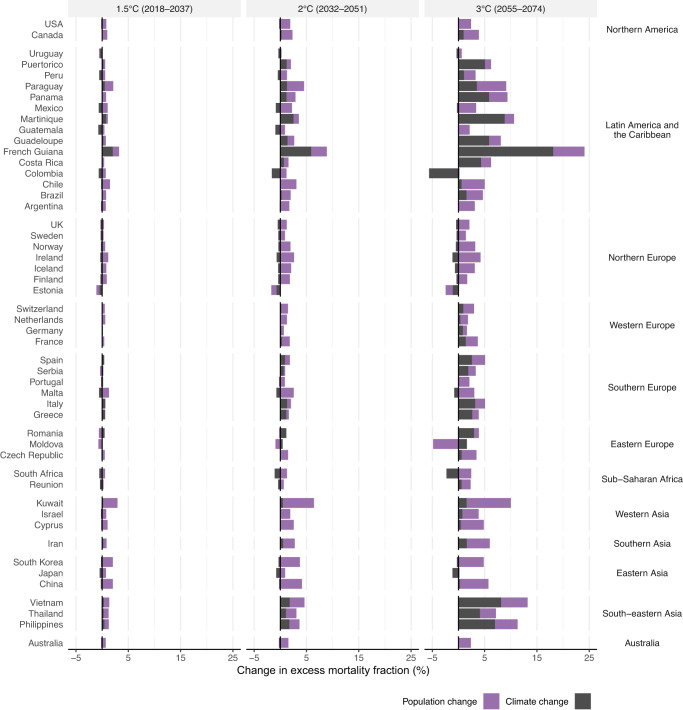
Contributions of climate change and population change to the changes in non-optimal temperature-related (heat and cold combined) mortality at different levels of global warming. Country/area-level changes by climate change and population aging are shown at 1.5 °C, 2 °C, and 3 °C of global warming using 20-year window compared with the historical period 1995-2014 under SSP5-8.5. The future periods in which the 20-year running mean of global mean temperature first reaches the 1.5 °C, 2 °C, and 3 °C of warming above pre-industrial level (1850–1900) are 2018–2037, 2032–2051, and 2055–2074, respectively under SSP5-8.5. The impact of population aging was estimated by subtracting the future changes in temperature-related impacts in the constant population scenario (climate-only) from the changes in temperature-related impacts under SSP5 mortality projection in the climate-population scenario.

For the non-optimal (cold and heat) temperature-related mortality, population aging alone results in an average of 0.8% (95% eCI: 0.6% to 0.9%), 1.7% (1.2% to 2.1%), and 2.6% (0.9% to 3.5%) increases in future temperature-related excess mortality when global warming reaches 1.5 °C, 2 °C, and 3 °C (Fig. 6 and Supplementary Table 7). In comparison, climate alone accounted for 3.0% (i.e., 0.08%/2.67%) of the net changes in non-optimal temperature-related mortality under the highest level (3 °C) of warming. Significantly positive estimations on mortality burden due to population aging were observed in about two-thirds (36 under all warming levels) of the countries/areas, whereas significantly negative contributions due to population aging were observed in very few countries/areas (5, 3, and 2 at 1.5 °C, 2 °C, and 3 °C, respectively). The larger role of population aging in future non-optimal temperature-related mortality may outweigh the differences in temperature rises observed between the SSP5-8.5 and SSP3-7.0 scenarios under the same global warming level, culminating in similar results across these scenarios.

## Discussion

In this study, we projected the future temperature-related mortality burden and quantified the contribution of population aging in 50 countries/areas at different global warming levels (1.5 °C, 2 °C, and 3 °C). Our analyses revealed an increasing temperature-related mortality burden at all warming levels studied when accounting for changes in population demographics. Population aging substantially amplifies future heat- and cold-related mortality burdens. Compared to climate change, population aging plays a dominant role in future cold-related mortality burden and would lead to a net increase in future cold-related mortality for all study locations combined.

Most of the temperature-related mortality burden can be attributable to cold rather than heat exposure, as shown in previous studies^6,7^. Thus, our findings strongly underline and support the need to account for a significant shift in the number of older individuals who will die from either cold or heat globally, regardless of whether we see large or small changes in climate. Without acknowledging the shifting population and the increasing number of people exposed to non-optimal temperatures (both heat and cold), the ability to address the health impacts of temperature extremes will be hindered. As observed in many previous studies^8–10^, in some locations, population exposed to warmer climates will lead to a reduction in cold-related mortality burden that could outnumber the expected increase in heat-related mortality burden. However, our study shows that accounting for population aging leads to an attenuation of this reduction or even an increase in cold-related mortality burden, resulting in a substantial increase in temperature-related mortality. Consistent with our analysis, the few previous studies considering population aging projected net increases in future temperature-related mortality burden under the Special Report on Emissions Scenarios (SRES)^11,12^ and Representative Concentration Pathway (RCP)^13,14^, global warming scenarios that were used in the previous IPCC Fourth and Fifth Assessment Reports.

Our findings demonstrate that accounting for population aging reveals a globally consistent increase in overall temperature-related mortality burden for any increase in global warming. Due to the dominant role of population aging, the net increases in temperature-related mortality were evident in 45 countries/areas at the 2 °C global warming and in 40 countries/areas at the 1.5 °C global warming (Fig. 5 and Supplementary Table 6). On average, increases in heat-related mortality fractions are larger than those in cold-related mortality fractions at the 1.5 °C and 2 °C warming. The comparison between the impact of 1.5 °C and 2 °C on overall temperature-related health under climate change alone could be highly uncertain and vary geographically^15^. This poorly understood difference between the health impact of 1.5 °C and 2 °C was recognized in the IPCC Special Report on Global Warming of 1.5 °C as a key knowledge gap in climate change and health research^16^. After taking population aging into account, the projected temperature-related mortality burden in most (47/50) studied countries/areas is consistently larger at 2 °C than at 1.5 °C of global warming (Fig. 5 and Supplementary Table 6). Our study thus helps fill this knowledge gap in understanding the difference between the projected health risks at 1.5 °C and 2 °C.

To the best of our knowledge, this is the first global study to specifically account for population aging in projection of future temperature-related mortality at global warming levels below and above the goals set by the Paris Agreement. This is also one of the few studies utilizing a larger ensemble of temperature projections from the latest generation of GCMs participating in the most recent climate modeling protocol (CMIP6). Our study is also the first study to quantify the contribution of population aging through both age group-specific temperature-mortality associations and projected baseline age group-specific mortality under the SSP emission and population scenarios. Our estimates are based on an extensive dataset with more than 83 million deaths from 50 countries/areas across five continents, enabling us to assess the vulnerability of each studied population through localized temperature-mortality associations. Other strengths include a state-of-the-art epidemiological analytical method, statistical downscaling techniques, population aging projections, and an advanced approach to account for uncertainty in both temperature-mortality relationships and variability across GCMs^9^.

Our study has several limitations. First, we did not consider potential population adaptation to heat^17^ given difficulties in defining and quantifying valid adaptation assumptions and mechanisms related to population-level adaptation to heat^18^. However, not accounting for adaptation allowed us to better disentangle the contribution of population aging and changes in the climate. Another limitation could be the unavailability of sub-national projections for population aging in all of our studied countries/areas. Moreover, our estimates may not necessarily represent country-specific average effects since most locations in the MCC database are urban areas and, in some cases, only one or two cities are available for a given country. Finally, due to the lack of or insufficient coverage of data, we were unable to obtain estimates of projected temperature-related mortality burden in most parts of South Asia and Africa, which will have the largest contribution of population change during the 21^st^ century^19,20^. Thus, our results of the population aging impact can be interpreted as conservative as we might expect larger impacts of population aging in those regions. Future international collaborations with researchers from these regions will help extend the MCC Network and address the existing data gaps.

As a major risk factor for many chronic diseases and conditions, population aging poses another threat to public health in addition to the looming climate crisis. The current global efforts to address climate change are by far insufficient to limit global warming to 1.5 °C. As of September 2021, the new and updated unconditional nationally determined contributions commitments and pledge announcements are estimated to limit global warming to 2.7 °C, inadequate to achieve the Paris Agreement’s goals^21^. Even full implementation of the newly announced national net-zero emission targets could only lower global warming to 2.0–2.4 °C by 2100^22^, leaving the 1.5 °C target beyond reach. We found that population aging will play a dominant role in determining the future temperature-related mortality burden, the higher the levels (1.5 °C, 2 °C, or 3 °C) of global warming, the greater the aging-induced temperature-related mortality burden. Our findings underscore the need for ambitious and drastic climate mitigation actions to keep 1.5 °C warming within reach and targeted and efficient climate adaptation measures to prevent temperature-related health impacts under the dual threats from climate change and population aging.

## Methods

### Daily meteorological and mortality data

Through the MCC Collaborative Research Network, we collected observed daily meteorological and mortality data from local weather stations and health authorities in each country, respectively (Supplementary Table 1). This dataset has been applied in previous publications on the association between temperature and daily mortality and future projections of temperature-related mortality under different Representative Concentration Pathways^9,15^. Daily counts of deaths due to non-external causes (International Classification of Diseases, 9th Revision codes 0-799 or 10th Revision codes A00-R99) or all-cause deaths were used as mortality data. Although we focus here on the non-external or all-cause deaths, previous studies have shown that the majority of mortality burden attributable to heat and cold are from cardiovascular and respiratory deaths (e.g., 68.3% in Jiangsu, China^23^). Supplementary Table 1 provides a detailed description of the data collection.

### Temperature projections and global warming levels

We obtained daily mean near-surface air temperature simulations for the period 1980 to 2100 from 18 general circulation models (GCMs) in the Copernicus Climate Change Service (C3S) Climate Data Store (CDS) based on the output of the Coupled Model Inter-comparison Project 6 (CMIP6)^2^. Eighteen GCMs (ACCES-CM2, AWI-CM-1-1-MR, BCC-CSM2-MR, CESM2, CNRM-CM6-1, CNRM-CM6-1-HR, CNRM-ESM-2-1, GFDL-ESM4, IITEM-ESM, INM-CM4-8, INM-CM5-0, IPSL-CM6A-LR, MIROC6, MIROC-ES2L, MPI-ESM1-2-LR, MRI-ESM2-0, NORESM2-MM, and UKESM1-0-LL) were selected because they have both historical simulations and future projections in the CDS under four climate scenarios that cover the range of possible future greenhouse gas emissions (i.e., SSP1-2.6, SSP2-4.5, SSP3-7.0, and SSP5-8.5), which are good representatives of the climate sensitivity of the whole CMIP6 ensemble. Temperature simulations from 18 GCMs were bias-corrected and statistically downscaled for each MCC location using the observed temperature data (included in the MCC database) and a recently proposed trend-preserving approach^3,4^. We used the daily time-series of temperatures simulations under the historical (1995–2014) and the SSP5-based and SSP3-based scenarios that achieves forcing levels of 8.5 W m^−2^ (SSP5-8.5) and 7.0 W m^−2^ (SSP3-7.0)^24^, respectively by the end of this century. Consistent with the estimates in the IPCC Sixth Assessment Report^25^, we determined the future periods in which the 20-year running mean of global mean temperature first reaches the 1.5 °C, 2 °C, and 3 °C of warming above pre-industrial level (1850–1900) as 2018–2037, 2032–2051, and 2055–2074, respectively under SSP5-8.5; and 2021–2040, 2037–2056, 2066–2085, respectively under SSP3-7.0. To compare with the future 20-year time window, we used the period 1995–2014 as our historical period in this analysis.

### Population aging and baseline mortality projections

We obtained country-level projections of population size and mortality rates for three age groups (0–64, 65–74, and ≥75 years) under the SSP5 (Fossil-fueled development) and the SSP3 (Regional rivalry) scenarios^26^ from the SSP Database - Version 2.0 (https://secure.iiasa.ac.at/web-apps/ene/SspDb/). This data was provided in 5-year bands from the year 2015 to the year 2100. For each SSP, we combined the country-age-specific mortality rate and population in each 5-year band and interpolated it into annual mortality by fitting a smoothing function (i.e., natural spline with 1 df every 10 years). We then transformed the country-age-specific mortality to the location-age-specific mortality by applying two factors: 1) an “age factor” using the projected mortality in 2015, since the MCC dataset does not include age-specific deaths, which corresponds to the share of deaths in each age category (0–64, 65–74, and ≥75 years) over the total, 2) “country-to-location” factor corresponding to the share of total deaths in each location vs. the country total. This second factor was estimated using the average annual mortality in each location using the available data between 1994–2015 and the country-specific mortality from 2015. In this way, we considered the mortality in 2015 to be held constant across the historical period between 1995–2014. In the last step, we retrieve the daily mortality from the annual series by applying a third factor “seasonal factor” corresponding to the average share of mortality by day of the year over the total annual mortality in the observed series in each location. By applying this factor, we preserve the seasonal cycle of mortality with a higher number of deaths during the winter season compared to the summer season. To disentangle the impact of population aging from climate change, we also computed the future baseline mortality without considering any population change by replicating the daily series of mortality counts in 1995–2014 along the century until 2100.

### Temperature-mortality associations

We used a two-stage time-series analysis, the state-of-the-art methodology in multi-location assessments, to estimate the overall association between daily temperature and mortality in each location, i.e., the exposure-response function (ERF). In the first stage, we applied a generalized linear model with quasi-Poisson family and distributed lag non-linear models (DLNM) to estimate location-specific temperature-mortality associations using observed data included in the MCC database. Consistent with previous studies^6,17^, the time-series model included a natural cubic spline with eight degrees of freedom (df) per year to control the long-term and seasonal time trend, and indicator variables for the day of the week. We modeled the complex non-linear and delayed relationship between temperature and mortality following the DLNM methodology^9,27^. Specifically, the time-series model equation is as follows:

1 $${Log}\left(E\left({Y}_{i,t}\right)\right]=	\alpha+{cb}\left({{temperature}}_{i,t},{lag}=21\right) \\ 	+{ns}\left({{date}}_{t},{df}=\frac{8}{{year}}\times {Nyears}\right)+\beta {DOW}$$

where Y_i,t_ is the daily counts of deaths on day *t* in location *i*; α is the intercept; *cb()* is the cross-basis function for temperature with a natural cubic spline with three internal knots placed at the 10^th^, 75^th^, and 90^th^ percentiles of the temperature distribution, and lag-response relationship with a natural cubic spline with three internal knots at equally-spaced log-scale values over 21 days of lag^9^; *ns()* is the natural cubic spline for *date*_*t*_ with eight df per year (a total of *Nyears* years) to control for both long-term trends and seasonality; and *DOW*_*i,t*_ represents the day of the week.

In the second stage, we pooled city-specific estimates using a novel two-level hierarchical random-effects meta-regression model^28^. The model accounted for location-specific fixed-effects meta-predictors including average temperature, temperature interquartile range, and country-level gross domestic product, and applied two-level random effects with cities nested within country-specific climate zones^29^ to account for heterogeneity across both. We then estimated the best linear unbiased predictions (BLUPs) for each location, which represents a trade-off between location-specific association from the first stage and the pooled association from the meta-regression^30^. Based on BLUPs, we calculated the minimum mortality temperature (MMT) for each location, which is the temperature corresponding to the lowest risk of mortality. We restricted MMT to be within the 2^nd^-98^th^ percentile range to avoid imprecisely estimated tails of the ERF^31^. We then retrieve age-specific relationships using the output of a dose-response multivariate meta-analytical model derived in a recent work of the consortium. The factors obtained from this meta-analytical model allowed us to predict the temperature-mortality associations for the 0–64, 65–74, and ≥75 years age groups, respectively (see Supplementary Text).

### Health impact assessment

We estimated the number of deaths and the corresponding heat-related and cold-related excess mortality fraction in each location for the historical and future 20-year periods using the standard methodology for quantification of health impacts^32^. Briefly, we applied the location-age-specific ERFs and the modeled daily series of temperature projections under SSP5-8.5 or SSP3-7.0 and baseline daily mortality projections to calculate the daily number of temperature-related excess deaths. For each 20-year period (historical and future decades corresponding to each warming level), we computed the total number of heat-related and cold-related excess deaths (EN) by summing the contributions from all the days of the series when the mean daily temperature was above or below the location-specific MMT, respectively. We then calculated the corresponding excess mortality fraction (EF - %) by dividing the estimated EN by the total number of deaths in each location. We also obtained mortality estimates aggregated by country- and overall across the 800 locations.

We then computed the future changes in EF as the difference between the EF estimated in each future period (corresponding to global warming targets (1.5 °C, 2 °C, and 3 °C)) and the historical period for each GCM and location. Additionally, we calculated EF in each future 20-year period assuming no changes in population growth and demographic structure (i.e. assuming the daily baseline mortality in the historical period) as done in Gasparrini et al.^9^. We finally estimated the impact of population aging by subtracting the future changes in temperature-related impacts in the constant population scenario (climate-only) from the change in EF derived under SSP5 or SSP3 mortality projections in the climate-population scenario. In other words, the climate-only scenario holds all populations constant at the baseline year 2015’s size, demographic structure, and age-specific mortality rate, whereas the climate-population scenario assumes changes in population size, demographic structure, and age-specific mortality rate in the future periods under SSP5 or SSP3. We estimated the empirical confidence intervals (eCI) using a set of 1000 simulated coefficients defining the ERF derived from Monte Carlo simulations. For each GCM scenario, we estimated the corresponding distribution of impacts and then derived the lower and upper bound of the ECI as the 2.5th and 97.5th percentiles of the ensemble distribution. In this way, we account for the uncertainty in ERFs and the variability across the 18 GCMs^9^.

### Reporting summary

Further information on research design is available in the Nature Portfolio Reporting Summary linked to this article.

### Supplementary information

Supplementary Information

Reporting Summary

## Data Availability

All data needed to evaluate the conclusions in the paper are present in the paper and/or the Supplementary Materials. Data were collected within the MCC Collaborative Research Network under a data sharing agreement and cannot be made publicly available.

## Code availability

A sample of the analysis code is available from https://github.com/CHENlab-Yale/MCC_ProjAging_Temp.

## References

[1] de Schrijver, E. et al. Nationwide projections of heat- and cold-related mortality impacts under various climate change and population development scenarios in Switzerland. *Environ. Res. Lett*. **18**, 12 (2023). 10.1088/1748-9326/ace7e1 PMC7616072 38854588

[2] Eyring V (2016). Overview of the Coupled Model Intercomparison Project Phase 6 (CMIP6) experimental design and organization. Geosci. Model Dev..

[3] Vicedo-Cabrera AM, Sera F, Gasparrini A (2019). Hands-on tutorial on a modeling framework for projections of climate change impacts on health. Epidemiology.

[4] Lange S (2019). Trend-preserving bias adjustment and statistical downscaling with ISIMIP3BASD (v1.0). Geosci. Model Dev..

[5] Samir K, Lutz W (2017). The human core of the shared socioeconomic pathways: Population scenarios by age, sex and level of education for all countries to 2100. Glob. Environ. Change.

[6] Gasparrini A (2015). Mortality risk attributable to high and low ambient temperature: a multicountry observational study. Lancet.

[7] Burkart KG (2021). Estimating the cause-specific relative risks of non-optimal temperature on daily mortality: a two-part modelling approach applied to the Global Burden of Disease Study. Lancet.

[8] Weinberger KR (2017). Projected temperature-related deaths in ten large U.S. metropolitan areas under different climate change scenarios. Environ. Int.

[9] Gasparrini A (2017). Projections of temperature-related excess mortality under climate change scenarios. Lancet Planet Health.

[10] Gu S (2020). Projections of temperature-related cause-specific mortality under climate change scenarios in a coastal city of China. Environ. Int.

[11] Hajat S, Vardoulakis S, Heaviside C, Eggen B (2014). Climate change effects on human health: projections of temperature-related mortality for the UK during the 2020s, 2050s and 2080s. J. Epidemiol. Community Health.

[12] Vardoulakis S (2014). Comparative assessment of the effects of climate change on heat- and cold-related mortality in the United Kingdom and Australia. Environ. Health Perspect..

[13] Lee JY, Kim H (2016). Projection of future temperature-related mortality due to climate and demographic changes. Environ. Int..

[14] Rai M (2019). Impact of climate and population change on temperature-related mortality burden in Bavaria, Germany. Environ. Res Lett..

[15] Vicedo-Cabrera AM (2018). Temperature-related mortality impacts under and beyond Paris Agreement climate change scenarios. Clim. Change.

[16] Ebi, K., Campbell-Lendrum, D. & Wyns, A. *The 1.5 Health Report: Synthesis on Health & Climate Science In the IPCC SR1* 5 (IPCC, 2018).

[17] Vicedo-Cabrera AM (2018). A multi-country analysis on potential adaptive mechanisms to cold and heat in a changing climate. Environ. Int.

[18] Gosling SN (2017). Adaptation to climate change: A comparative analysis of modeling methods for heat-related mortality. Environ. Health Perspect..

[19] Population Division of the Department of Economic and Social Affairs. *2019 Revision of World Population Prospects* (Population Division of the Department of Economic and Social Affairs, 2019).

[20] Scovronick N (2017). Impact of population growth and population ethics on climate change mitigation policy. Proc. Natl Acad. Sci. USA.

[21] United Nations Environment Programme. *Emissions Gap Report 2021: The Heat Is On – A World of Climate Promises Not Yet Delivered* (Nairobi, 2021).

[22] Höhne N (2021). Wave of net zero emission targets opens window to meeting the Paris Agreement. Nat. Clim. Chang.

[23] Ma Y, Zhou L, Chen K (2020). Burden of cause-specific mortality attributable to heat and cold: A multicity time-series study in Jiangsu Province, China. Environ. Int..

[24] O’Neill BC (2016). The scenario model intercomparison project (ScenarioMIP) for CMIP6. Geosci. Model Dev..

[25] IPCC. *Climate Change 2021: The Physical Science Basis Contribution of Working Group I to the Sixth Assessment Report of the Intergovernmental Panel on Climate Change* (Cambridge University Press, 2021).

[26] Jiang L, O’Neill BC (2017). Global urbanization projections for the Shared Socioeconomic Pathways. Glob. Environ. Change.

[27] Gasparrini A, Armstrong B, Kenward MG (2010). Distributed lag non‐linear models. Stat. Med..

[28] Sera F, Armstrong B, Blangiardo M, Gasparrini A (2019). An extended mixed‐effects framework for meta‐analysis. Stat. Med..

[29] Kottek M, Grieser J, Beck C, Rudolf B, Rubel F (2006). World map of the Köppen-Geiger climate classification updated. Meteorologische Z..

[30] Gasparrini A, Armstrong B, Kenward MG (2012). Multivariate meta-analysis for non-linear and other multi-parameter associations. Stat. Med..

[31] Tobías A, Armstrong B, Gasparrini A (2017). Brief Report: Investigating uncertainty in the minimum mortality temperature: Methods and application to 52 Spanish cities. Epidemiology.

[32] Gasparrini A, Leone M (2014). Attributable risk from distributed lag models. BMC Med. Res. Methodol..

