# Supplementary material for: Impact of population aging on future temperature-related mortality at different global warming levels
Source: Nat Commun. 2024 Feb 27;15:1796. doi: 10.1038/s41467-024-45901-z (PMC10899213; doi:10.1038/s41467-024-45901-z)
Supplement: Supplementary file 1 — Supplementary Information [file 41467_2024_45901_MOESM1_ESM.pdf]

# Supplementary Information

## Impact of population aging on future temperature-related mortality at different global warming levels

Kai Chen, Evan de Schrijver, Sidharth Sivaraj, Francesco Sera, Noah Scovronick, Leiwen Jiang, Dominic Roye, Eric Lavigne, Jan Kyselý, Aleš Urban, Alexandra Schneider, Veronika Huber, Joana Madureira, Malcolm N. Mistry, Ivana Cvijanovic, MCC Collaborative Research Network; Antonio Gasparrini, and Ana M. Vicedo-Cabrera

*Corresponding author. Email: kai.chen@yale.edu

**This PDF file includes:**

Supplementary Methods
Supplementary Figures
Supplementary Tables

## Supplementary Methods

Age group-specific temperature-mortality relationships

To obtain the age group-specific relationships, we first quantified the location-specific relationship between daily mean temperature and mortality using observed data and a state-of-the-art two-stage approach coupled with distributed lag nonlinear models [1] and multivariate dose-response meta-regression [2,3]. We then applied age-specific exposure-response function (ERF) predicted from a meta-analytical model derived by Dr. Noah Scovronick. In detail, it was not possible to derive pooled ERF for specific age categories from the time series analysis (1st stage) as MCC mortality data by age are not available for all locations for the same age bands. Thus, Scovronick et al. applied a novel methodology consistent with a multivariate dose-response meta-analysis that allows for continuously modelling age as an effect modifier of the temperature-mortality association. Using the derived meta-regression model, we predicted the ERF at 50, 70, and 83.5 years old, corresponding to the average age at death for each interval, which represented the temperature-mortality association at the 0-64, 65-74, and ≥75 years age groups, respectively. Using the youngest age group (0-64 years) as the reference, we then calculated a set of ERF conversion factors for two older groups through dividing the ERFs at age 70 and 83.5 years by the ERF at age 50 years, respectively. To keep the non-linear shape of the overall ERF and to take into account the larger temperature range for cold than for heat (Figure S3), here we used the $10^{th}$ percentile of temperature for cold and the $99^{th}$ percentile of temperature for heat when calculating the ERF convention factors for cold and heat, respectively. These age group-specific ERF conversion factors were then applied to estimate the location-specific age-specific temperature-mortality associations based on the overall ERF derived from the two-stage analysis. Although this approach allows for the use of continuous risk estimates across the full temperature range, it relies on the strong assumption that difference in mortality risk between age categories remains the same across locations.

Compared with the 0-64 age group, the 65-74 and ≥75 years age groups have 1.2 and 1.8 times of heat-related mortality risks and 1.9 and 2.4 times of cold-related mortality risks. Applying these conversion factors to the location-specific total ERF, we obtained the age group-specific ERFs in each MCC location. Figure S3 reports the total and age group-specific exposure-response curves in 800 studied locations.

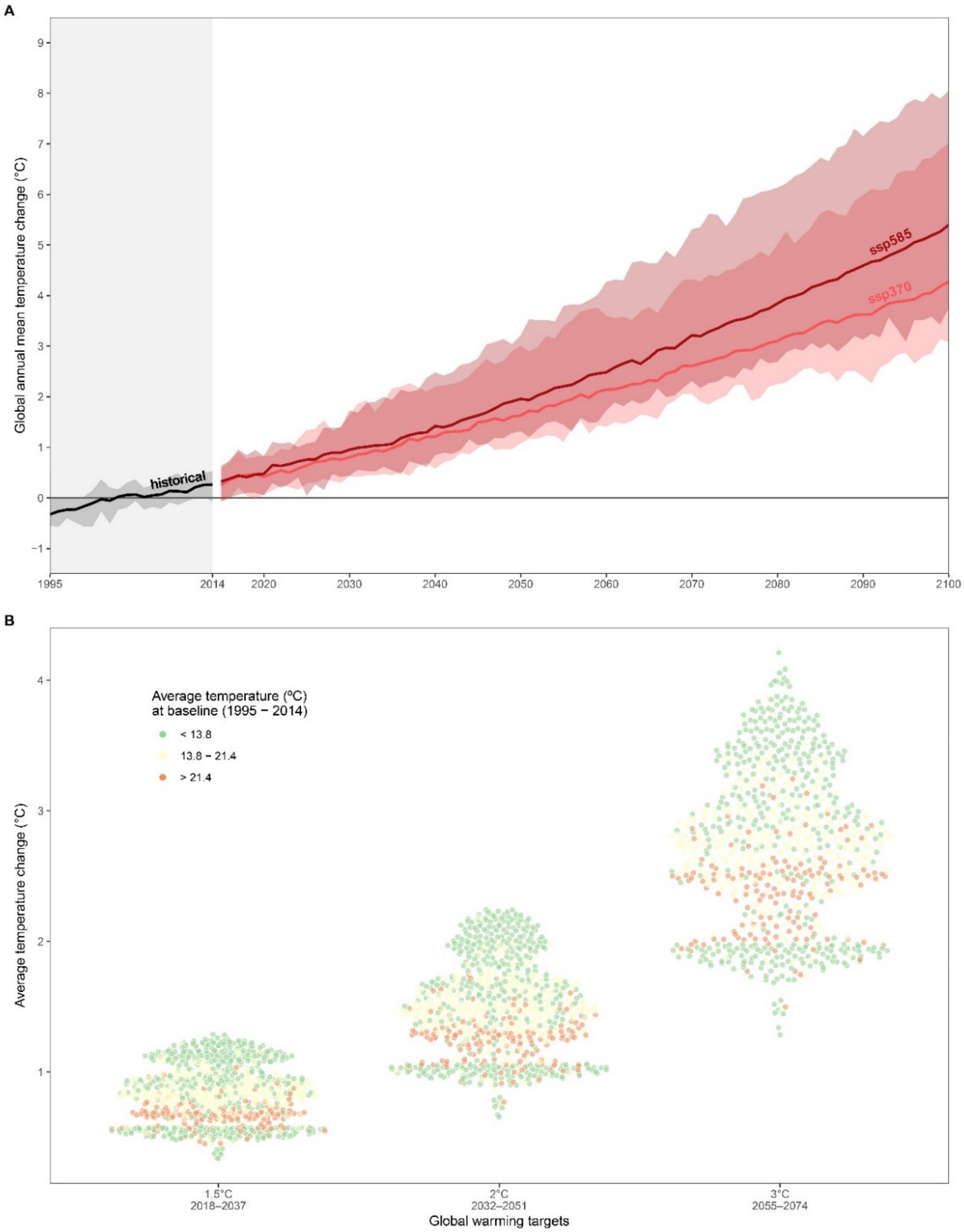


**Supplementary Figure 1. Projections of average daily mean temperature changes under global warming levels. (A) Global annual average temperature changes** relative to 1995-2014 (historical period, gray shaded area) under SSP5-8.5 and SSP3-7.0. Solid colored lines represent the multi-model ensemble average, and colored shades show the range of simulations from 18 global circulation models (GCMs). (**B**) **Location-specific average temperature changes** at different levels of global warming stratified by local average temperature at historical period (1995-2014).

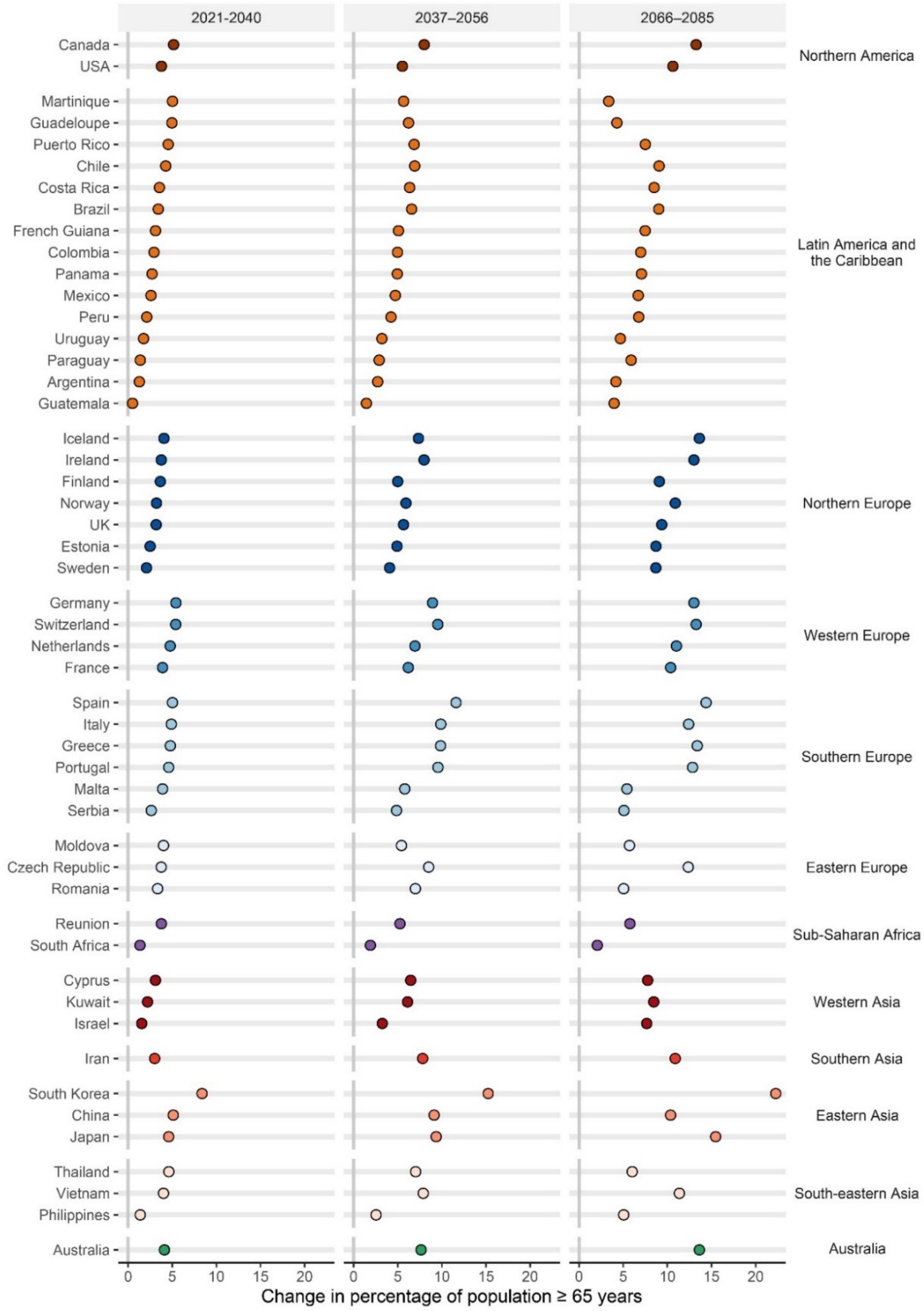


**Supplementary Figure 2. Population aging at periods corresponding to different levels (1.5 °C, 2 °C, and 3 °C – SSP3-7.5) of global warming by country/area.** Country-specific age-group population projections are derived from the SSP3 scenario in the first 20-year periods reaching 1.5 °C (2021-2040), 2 °C (2037–2056), and 3 °C (2066–2085) of warming, respectively.

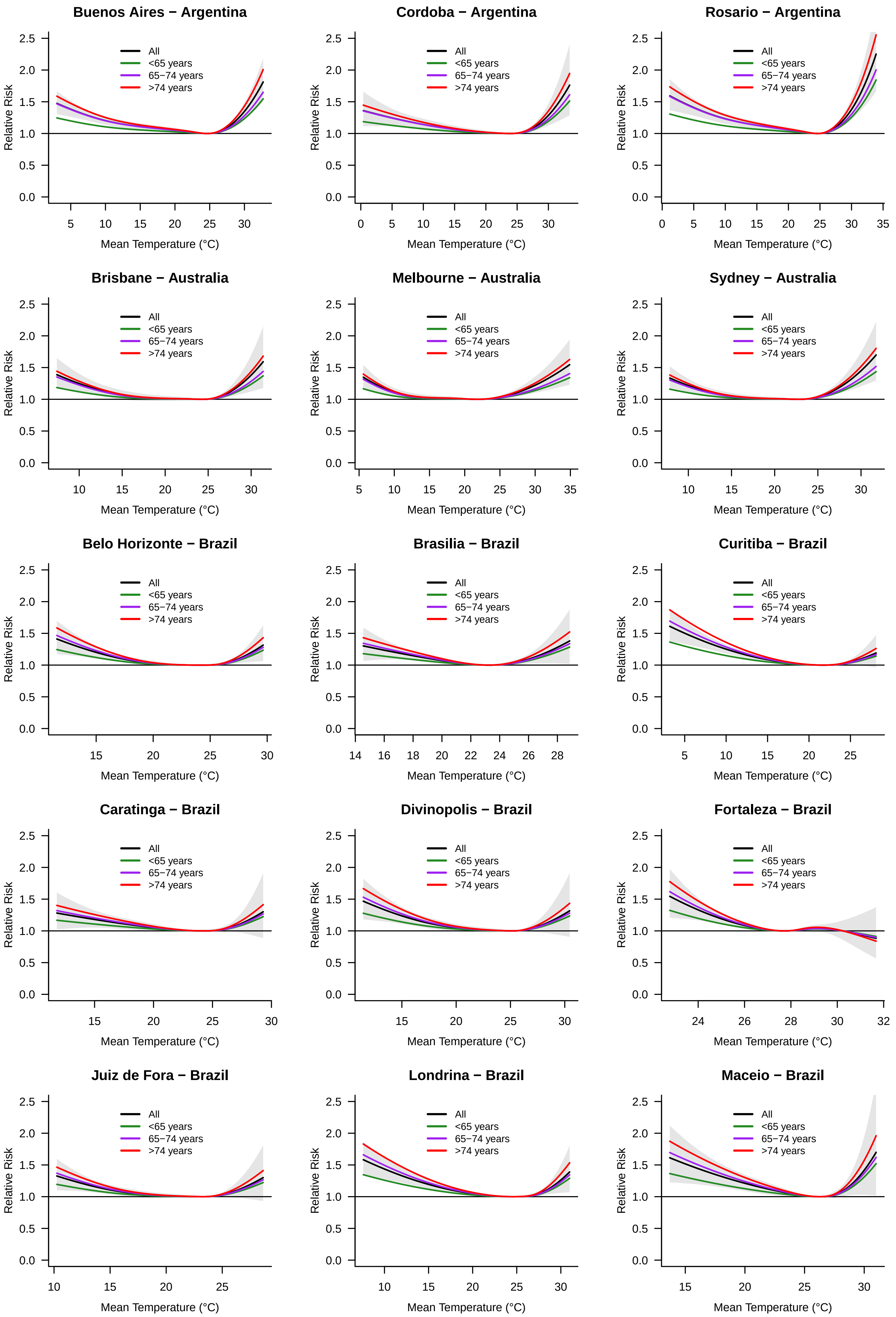

Buenos Aires − Argentina
Cordoba − Argentina
Rosario − Argentina
Brisbane − Australia
Melbourne − Australia
Sydney − Australia
Belo Horizonte − Brazil
Brasilia − Brazil
Curitiba − Brazil
Caratinga − Brazil
Divinopolis − Brazil
Fortaleza − Brazil
Juiz de Fora − Brazil
Londrina − Brazil
Maceio − Brazil
All
<65 years
65−74 years
>74 years
Relative Risk
Mean Temperature (°C)

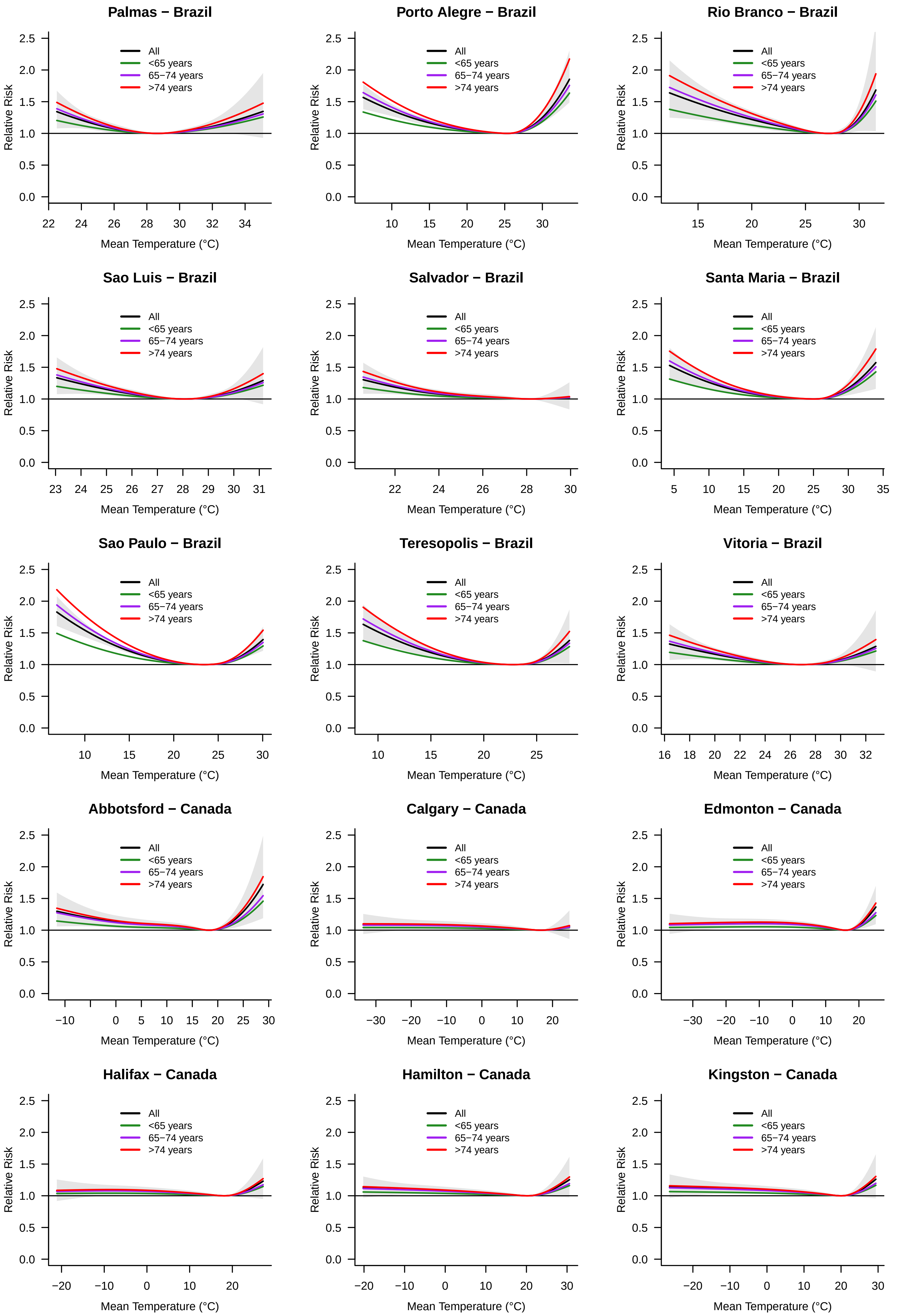
Palmas − Brazil
Porto Alegre − Brazil
Rio Branco − Brazil
Sao Luis − Brazil
Salvador − Brazil
Santa Maria − Brazil
Sao Paulo − Brazil
Teresopolis − Brazil
Vitoria − Brazil
Abbotsford − Canada
Calgary − Canada
Edmonton − Canada
Halifax − Canada
Hamilton − Canada
Kingston − Canada
All
<65 years
65−74 years
>74 years
Relative Risk
Mean Temperature (°C)

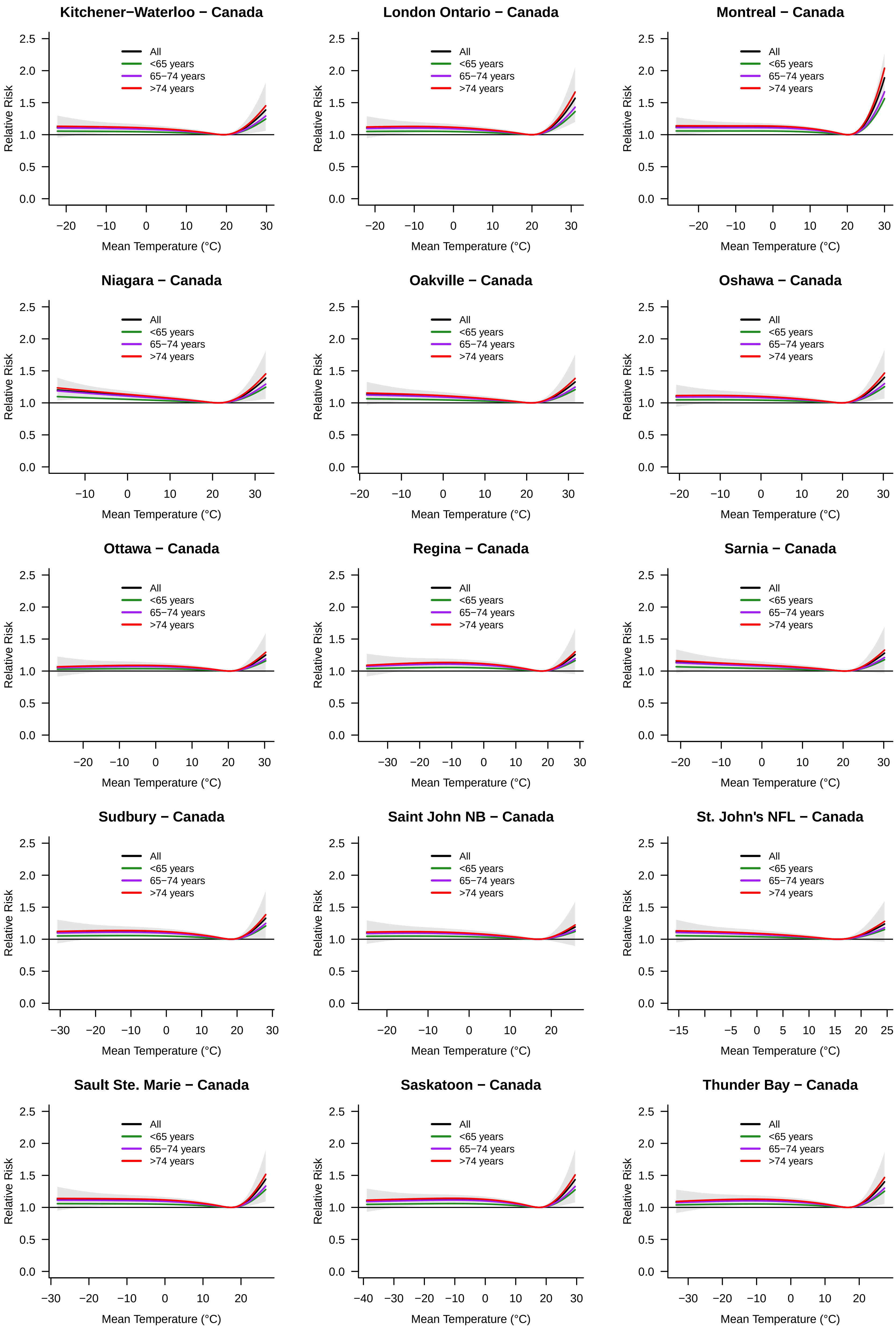
Kitchener−Waterloo − Canada
London Ontario − Canada
Montreal − Canada
Niagara − Canada
Oakville − Canada
Oshawa − Canada
Ottawa − Canada
Regina − Canada
Sarnia − Canada
Sudbury − Canada
Saint John NB − Canada
St. John's NFL − Canada
Sault Ste. Marie − Canada
Saskatoon − Canada
Thunder Bay − Canada
All
<65 years
65−74 years
>74 years
Relative Risk
0.0
0.5
1.0
1.5
2.0
2.5
Mean Temperature (°C)

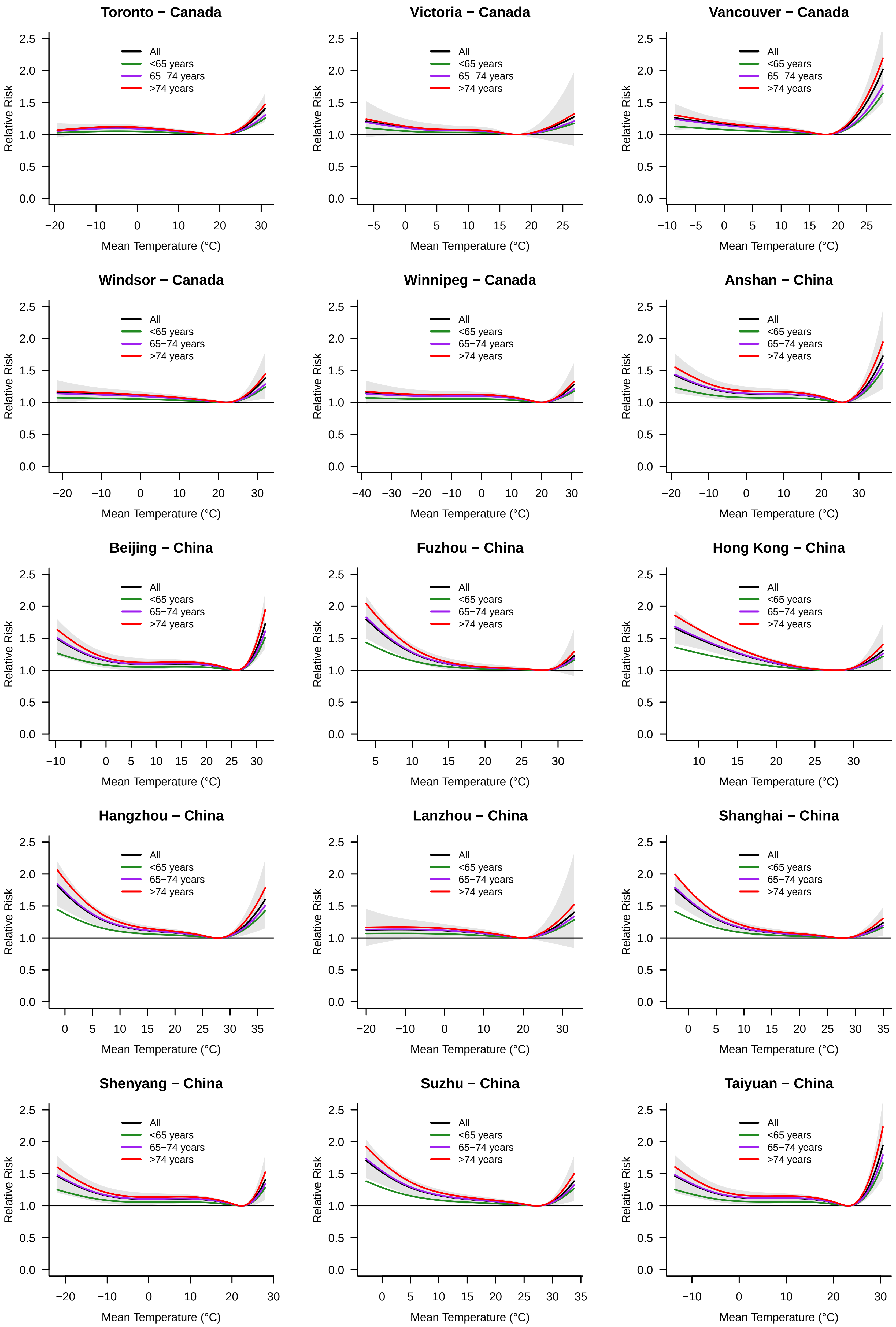

Toronto − Canada
Victoria − Canada
Vancouver − Canada
Windsor − Canada
Winnipeg − Canada
Anshan − China
Beijing − China
Fuzhou − China
Hong Kong − China
Hangzhou − China
Lanzhou − China
Shanghai − China
Shenyang − China
Suzhu − China
Taiyuan − China
All
<65 years
65−74 years
>74 years
Relative Risk
Mean Temperature (°C)

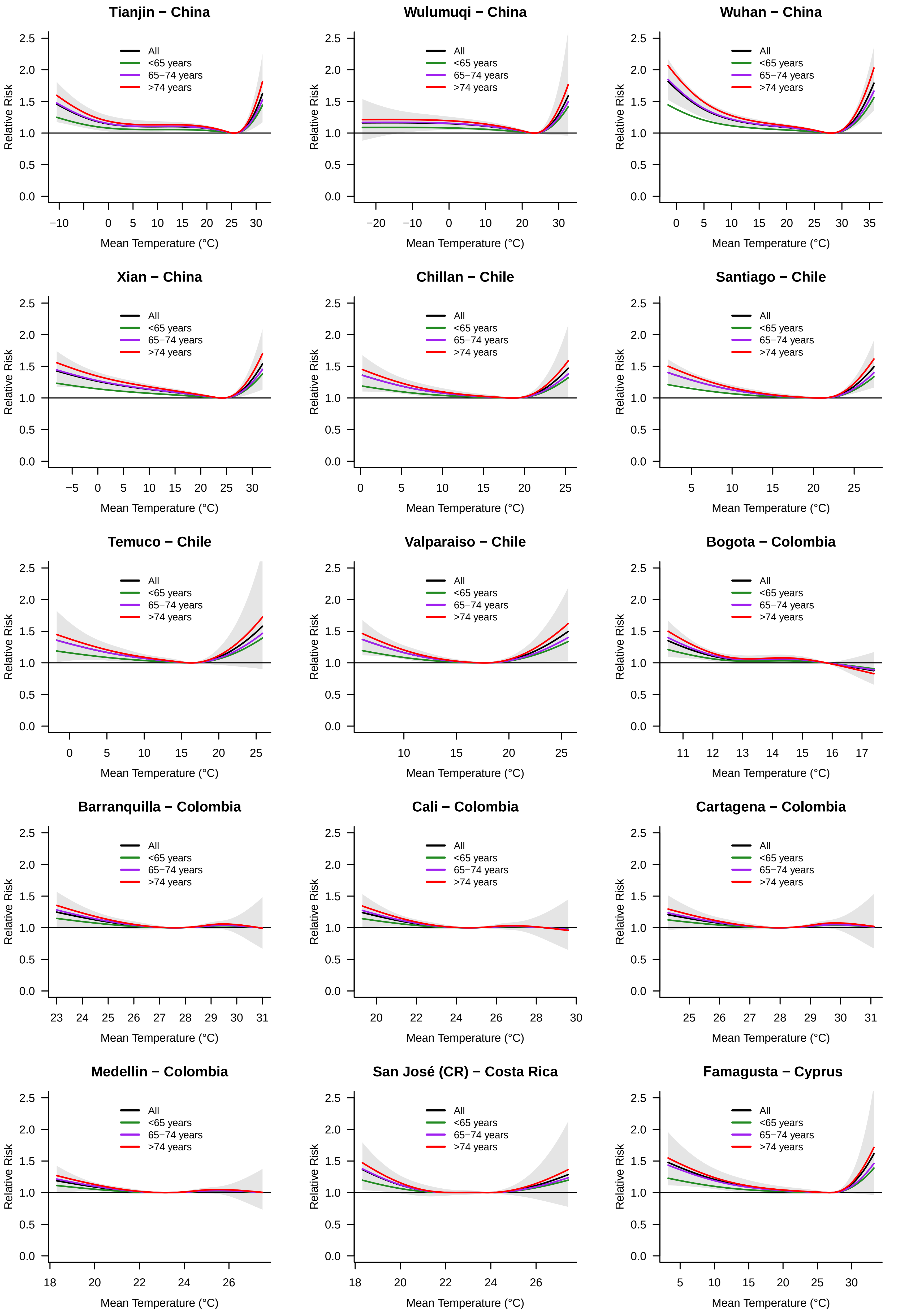
Tianjin − China
Wulumuqi − China
Wuhan − China
Xian − China
Chillan − Chile
Santiago − Chile
Temuco − Chile
Valparaiso − Chile
Bogota − Colombia
Barranquilla − Colombia
Cali − Colombia
Cartagena − Colombia
Medellin − Colombia
San José (CR) − Costa Rica
Famagusta − Cyprus
All
<65 years
65−74 years
>74 years
Relative Risk
Mean Temperature (°C)

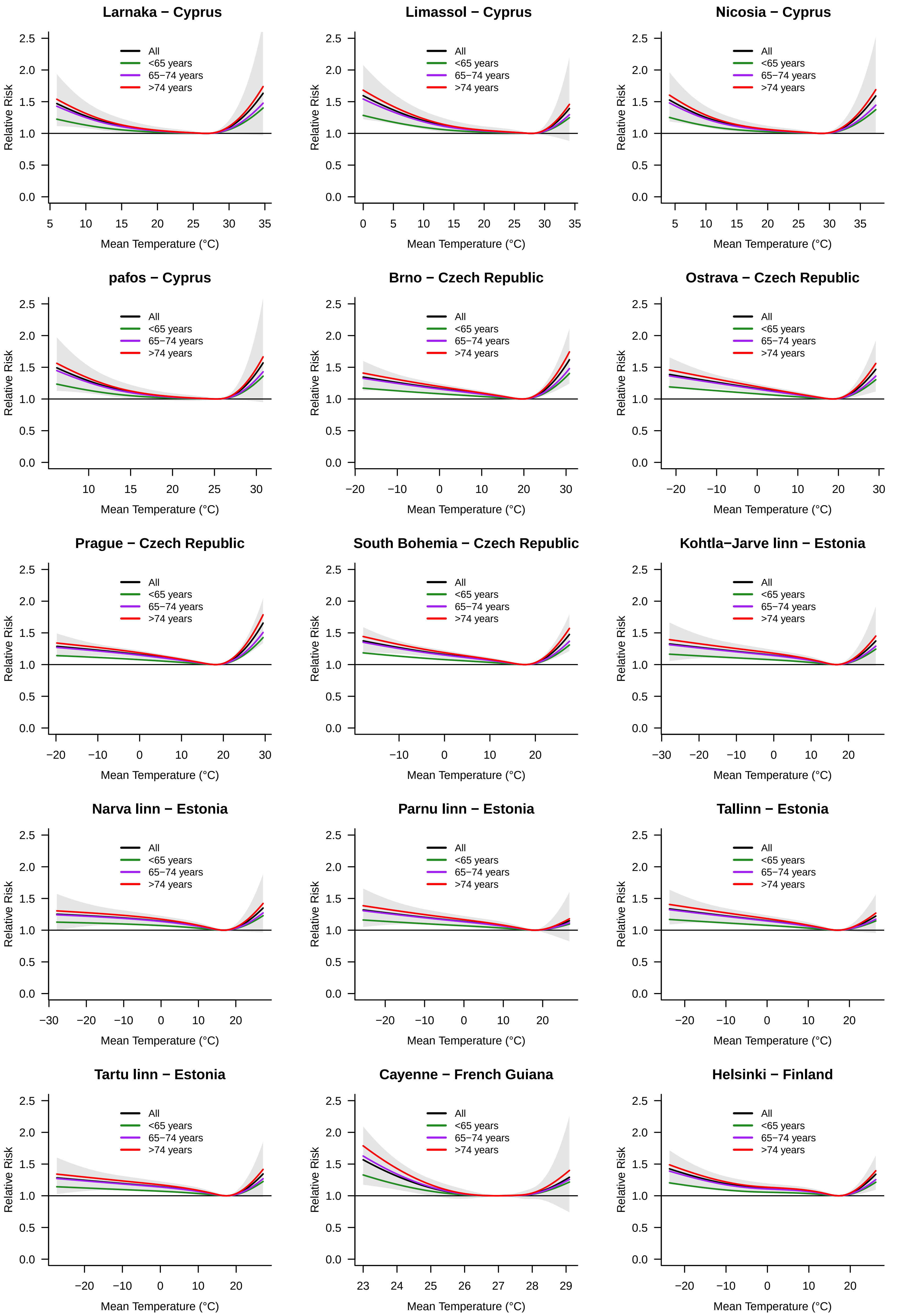
Larnaka − Cyprus
Limassol − Cyprus
Nicosia − Cyprus
pafos − Cyprus
Brno − Czech Republic
Ostrava − Czech Republic
Prague − Czech Republic
South Bohemia − Czech Republic
Kohtla−Jarve linn − Estonia
Narva linn − Estonia
Parnu linn − Estonia
Tallinn − Estonia
Tartu linn − Estonia
Cayenne − French Guiana
Helsinki − Finland
All
<65 years
65−74 years
>74 years
Relative Risk
Mean Temperature (°C)

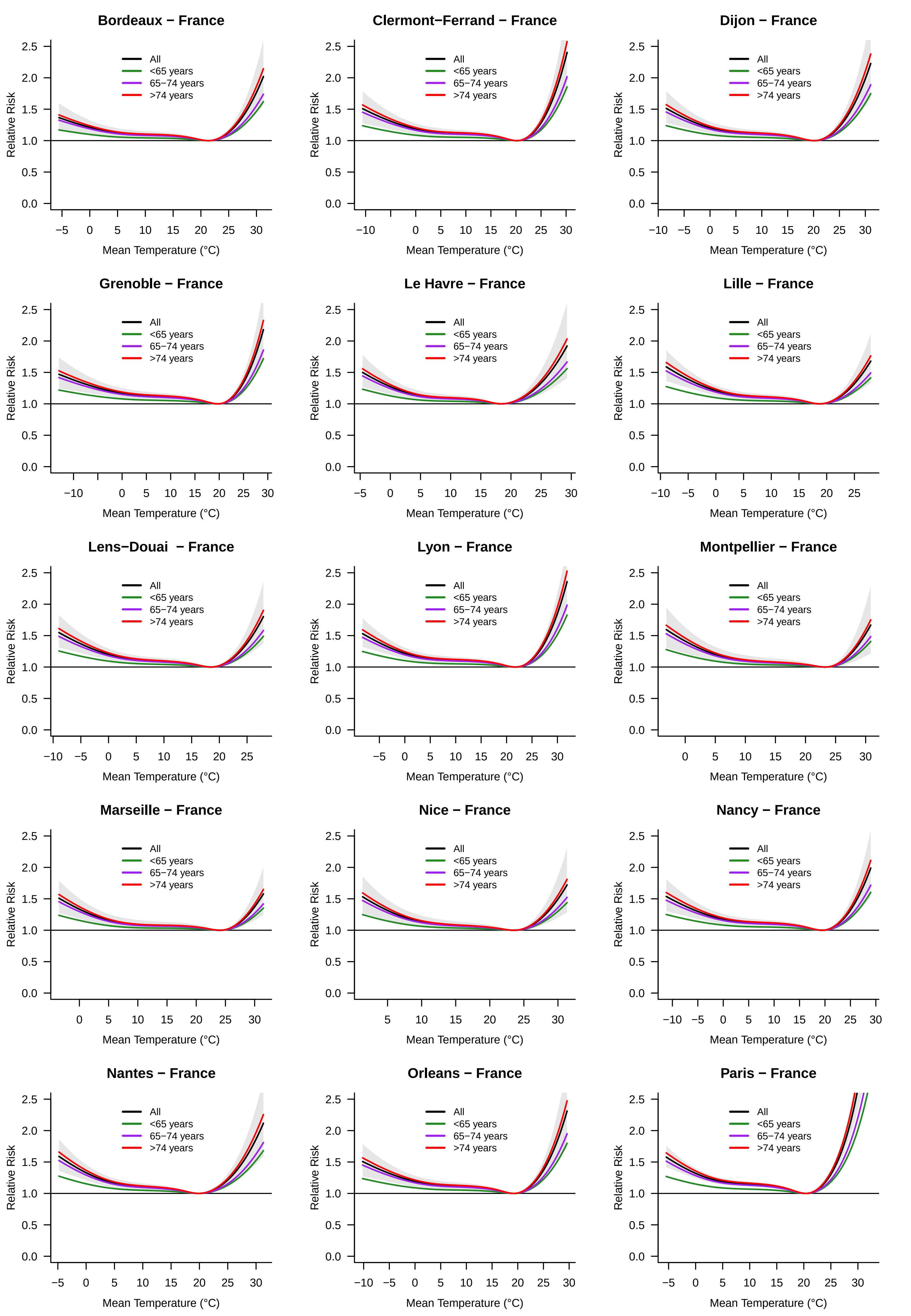
Bordeaux − France
Clermont−Ferrand − France
Dijon − France
Grenoble − France
Le Havre − France
Lille − France
Lens−Douai − France
Lyon − France
Montpellier − France
Marseille − France
Nice − France
Nancy − France
Nantes − France
Orleans − France
Paris − France
All
<65 years
65−74 years
>74 years
Relative Risk
Mean Temperature (°C)

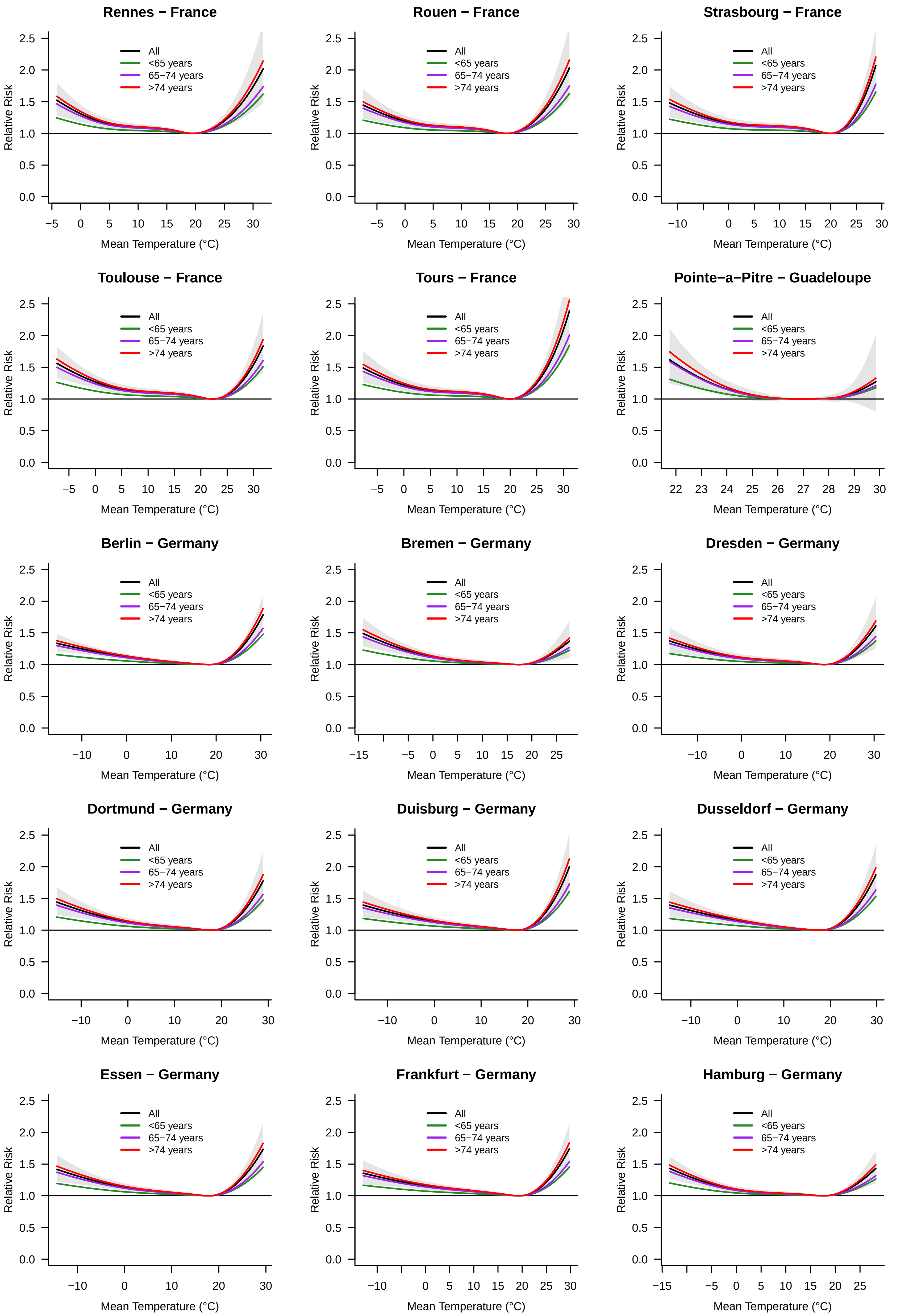

Rennes − France
Rouen − France
Strasbourg − France
Toulouse − France
Tours − France
Pointe−a−Pitre − Guadeloupe
Berlin − Germany
Bremen − Germany
Dresden − Germany
Dortmund − Germany
Duisburg − Germany
Dusseldorf − Germany
Essen − Germany
Frankfurt − Germany
Hamburg − Germany
All
<65 years
65−74 years
>74 years
Relative Risk
Mean Temperature (°C)

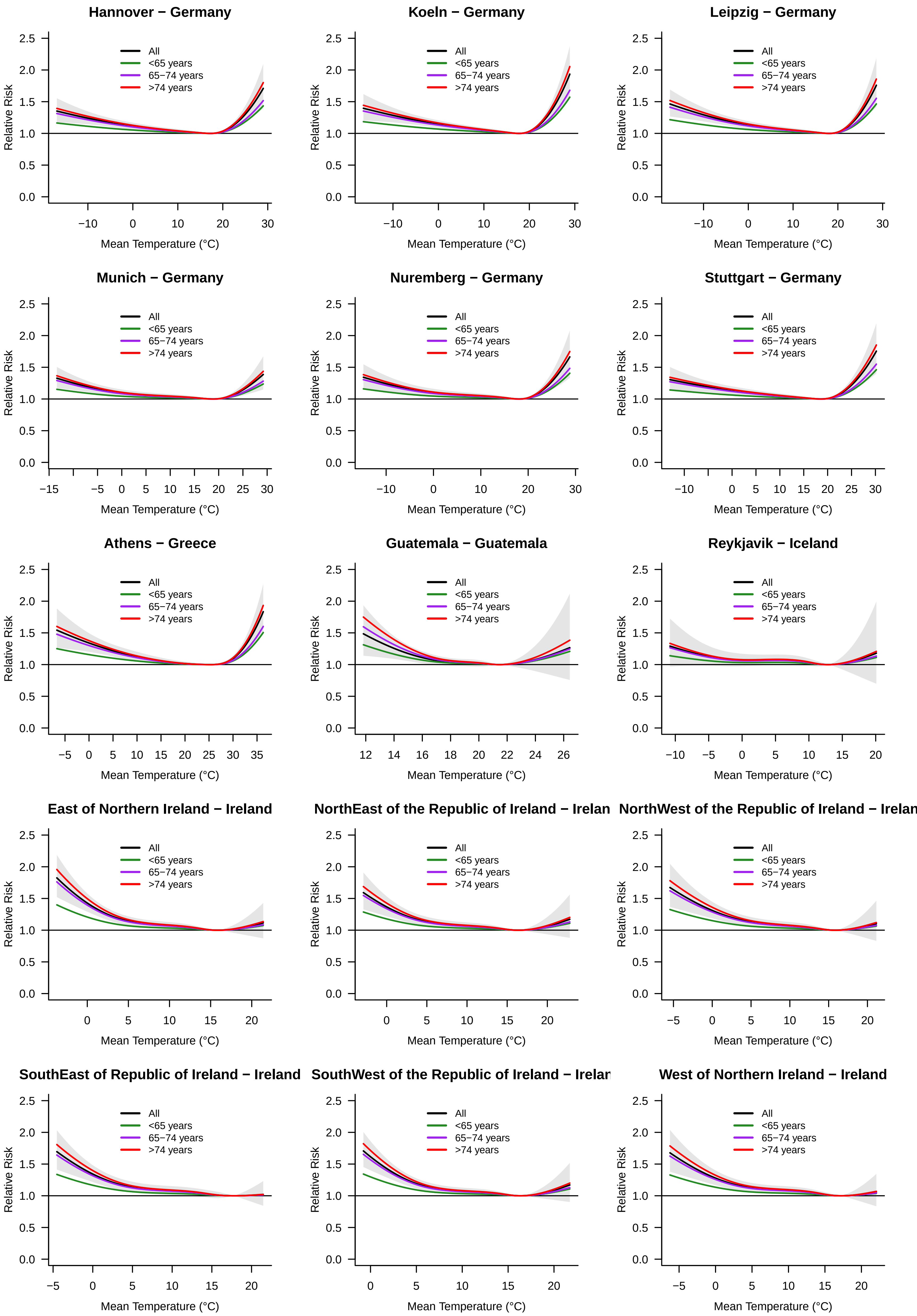
Hannover − Germany
Koeln − Germany
Leipzig − Germany
Munich − Germany
Nuremberg − Germany
Stuttgart − Germany
Athens − Greece
Guatemala − Guatemala
Reykjavik − Iceland
East of Northern Ireland − Ireland
NorthEast of the Republic of Ireland − Irelan
NorthWest of the Republic of Ireland − Irelan
SouthEast of Republic of Ireland − Ireland
SouthWest of the Republic of Ireland − Irelar
West of Northern Ireland − Ireland
All
<65 years
65−74 years
>74 years
Relative Risk
Mean Temperature (°C)

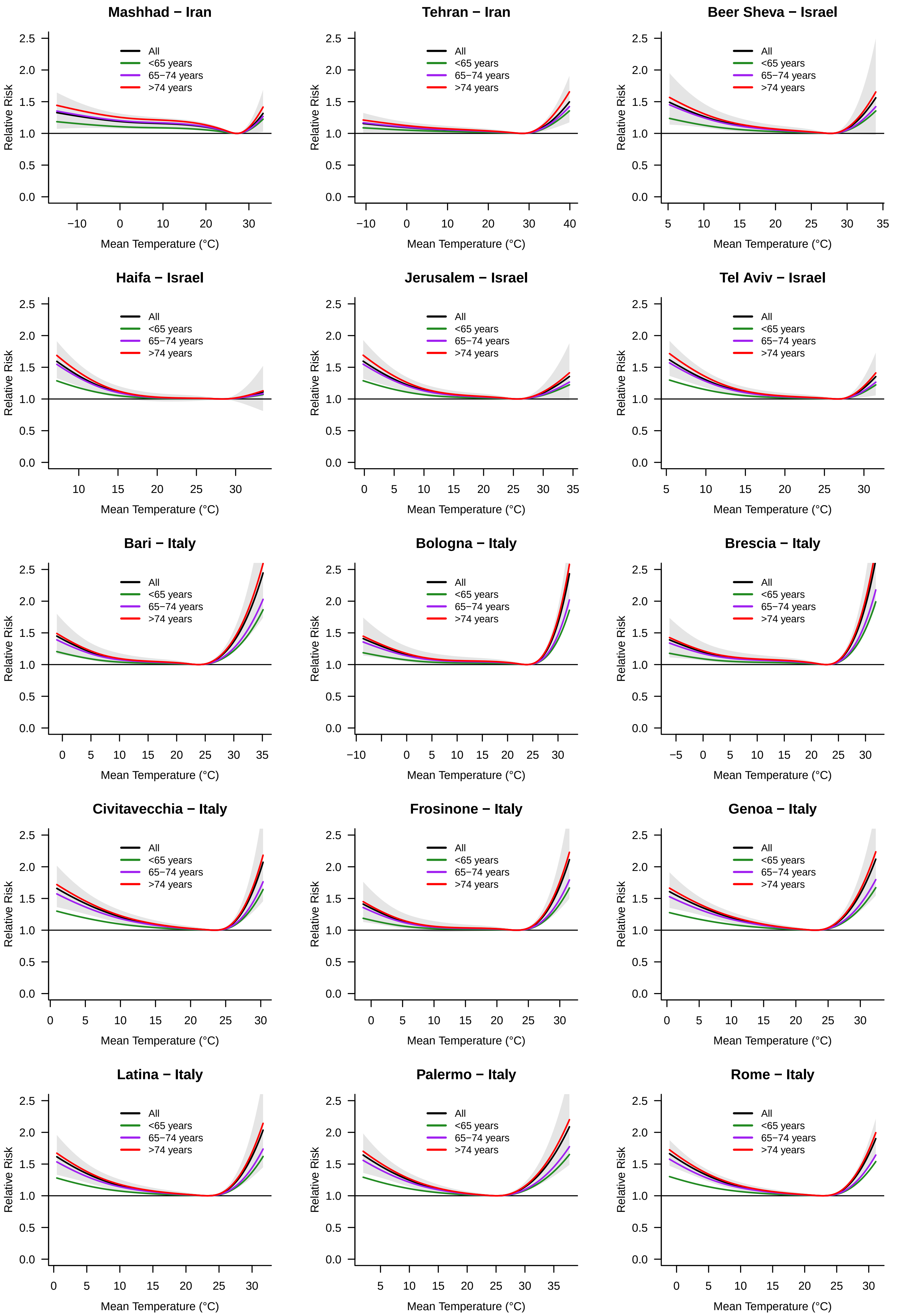
Mashhad − Iran
Tehran − Iran
Beer Sheva − Israel
Haifa − Israel
Jerusalem − Israel
Tel Aviv − Israel
Bari − Italy
Bologna − Italy
Brescia − Italy
Civitavecchia − Italy
Frosinone − Italy
Genoa − Italy
Latina − Italy
Palermo − Italy
Rome − Italy
All
<65 years
65−74 years
>74 years
Relative Risk
Mean Temperature (°C)

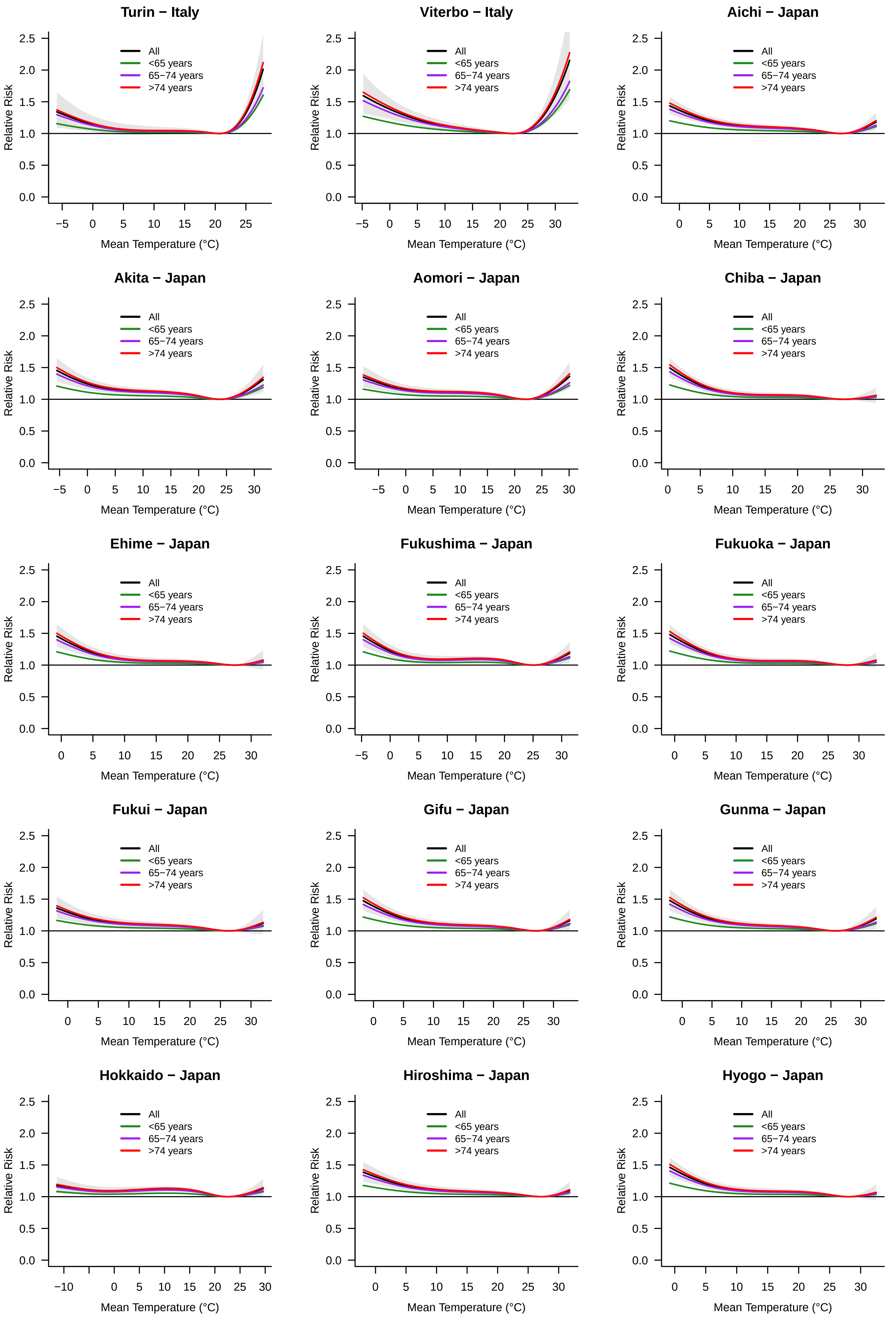
Turin − Italy
Viterbo − Italy
Aichi − Japan
Akita − Japan
Aomori − Japan
Chiba − Japan
Ehime − Japan
Fukushima − Japan
Fukuoka − Japan
Fukui − Japan
Gifu − Japan
Gunma − Japan
Hokkaido − Japan
Hiroshima − Japan
Hyogo − Japan
All
<65 years
65−74 years
>74 years
Relative Risk
Mean Temperature (°C)

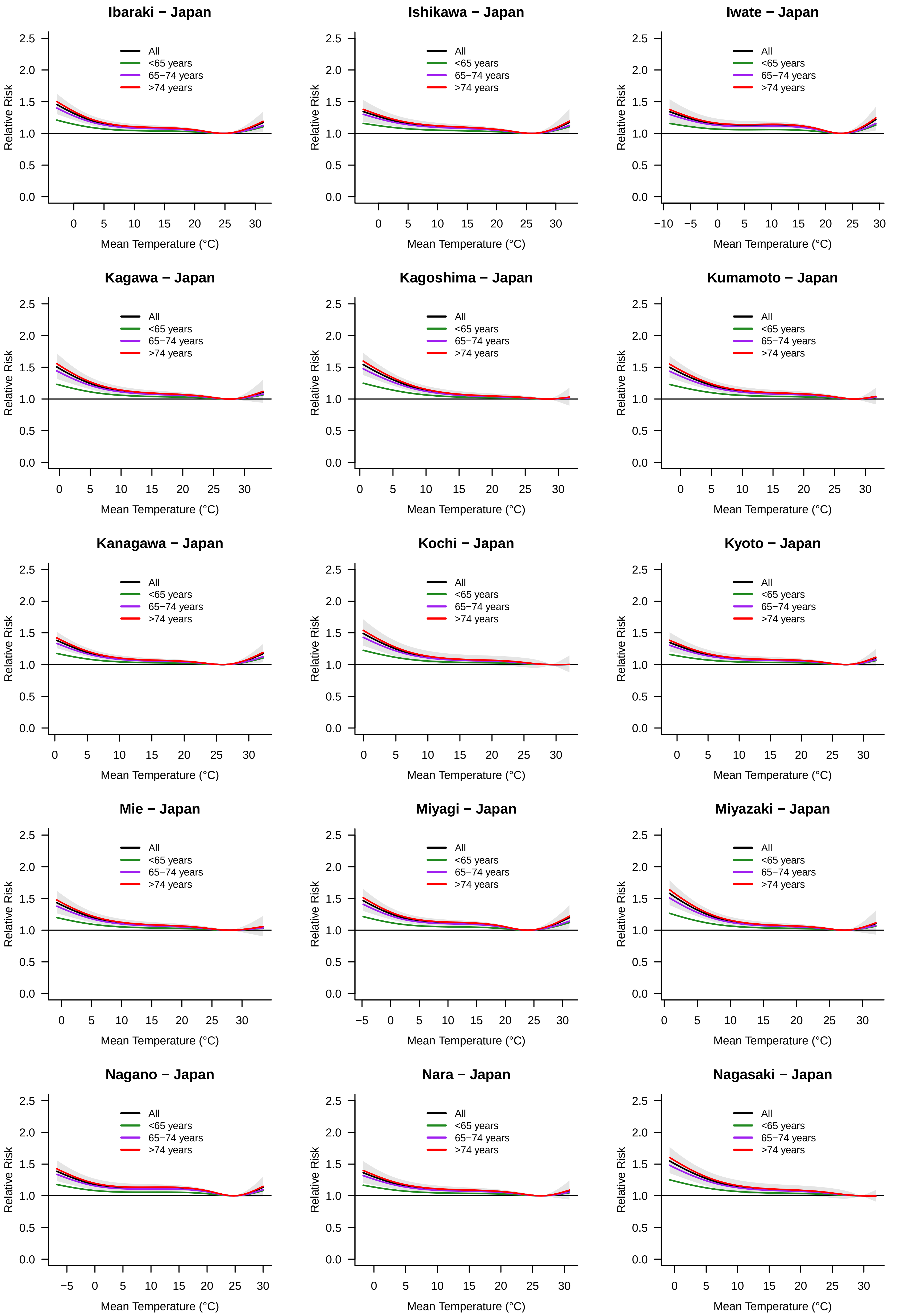
Ibaraki − Japan
Ishikawa − Japan
Iwate − Japan
Kagawa − Japan
Kagoshima − Japan
Kumamoto − Japan
Kanagawa − Japan
Kochi − Japan
Kyoto − Japan
Mie − Japan
Miyagi − Japan
Miyazaki − Japan
Nagano − Japan
Nara − Japan
Nagasaki − Japan
All
<65 years
65−74 years
>74 years
Relative Risk
Mean Temperature (°C)

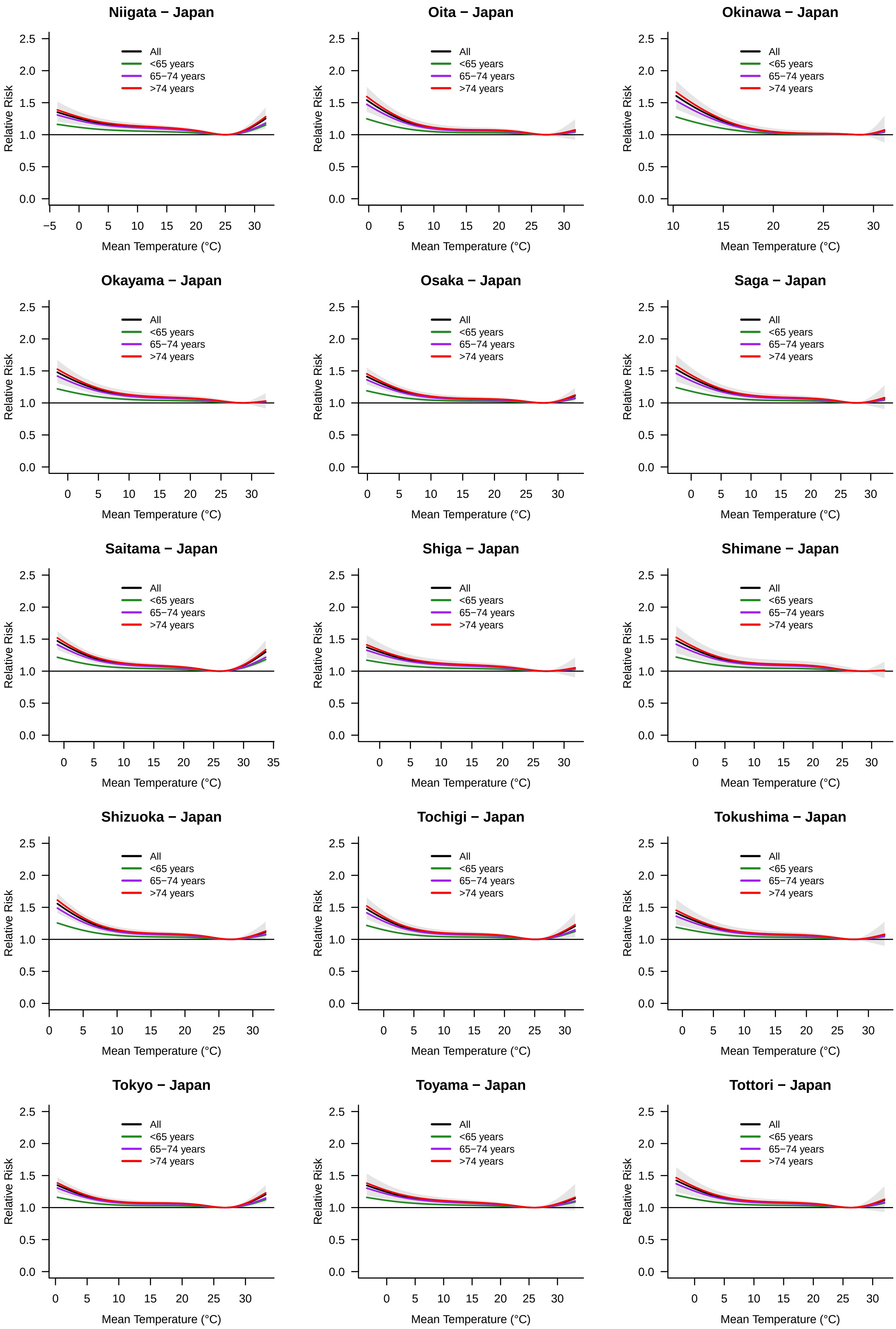
Niigata − Japan
Oita − Japan
Okinawa − Japan
Okayama − Japan
Osaka − Japan
Saga − Japan
Saitama − Japan
Shiga − Japan
Shimane − Japan
Shizuoka − Japan
Tochigi − Japan
Tokushima − Japan
Tokyo − Japan
Toyama − Japan
Tottori − Japan
All
<65 years
65−74 years
>74 years
Relative Risk
0.0
0.5
1.0
1.5
2.0
2.5
Mean Temperature (°C)
−5
0
5
10
15
20
25
30
35

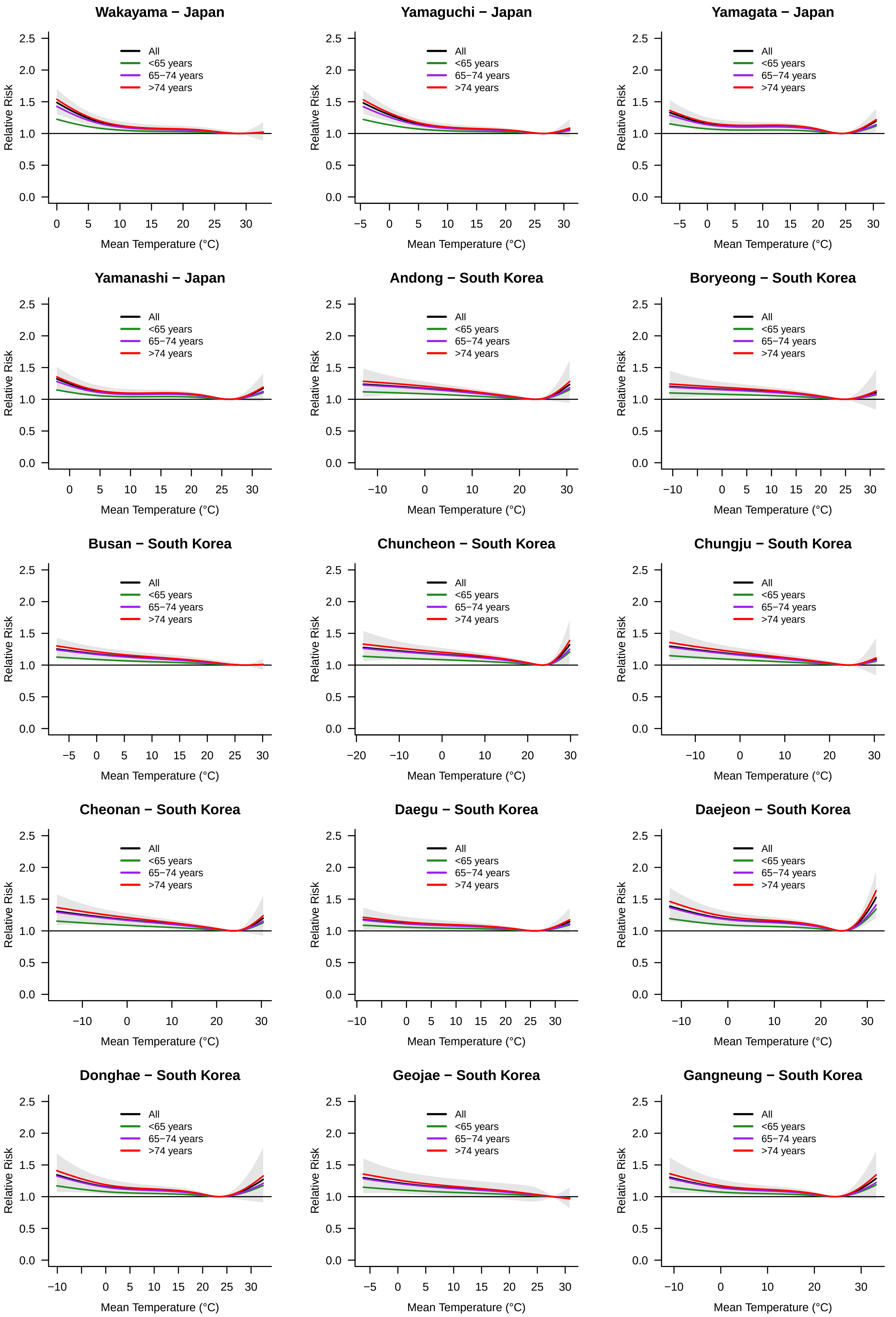
Wakayama − Japan
Yamaguchi − Japan
Yamagata − Japan
Yamanashi − Japan
Andong − South Korea
Boryeong − South Korea
Busan − South Korea
Chuncheon − South Korea
Chungju − South Korea
Cheonan − South Korea
Daegu − South Korea
Daejeon − South Korea
Donghae − South Korea
Geojae − South Korea
Gangneung − South Korea
All
<65 years
65−74 years
>74 years
Relative Risk
Mean Temperature (°C)

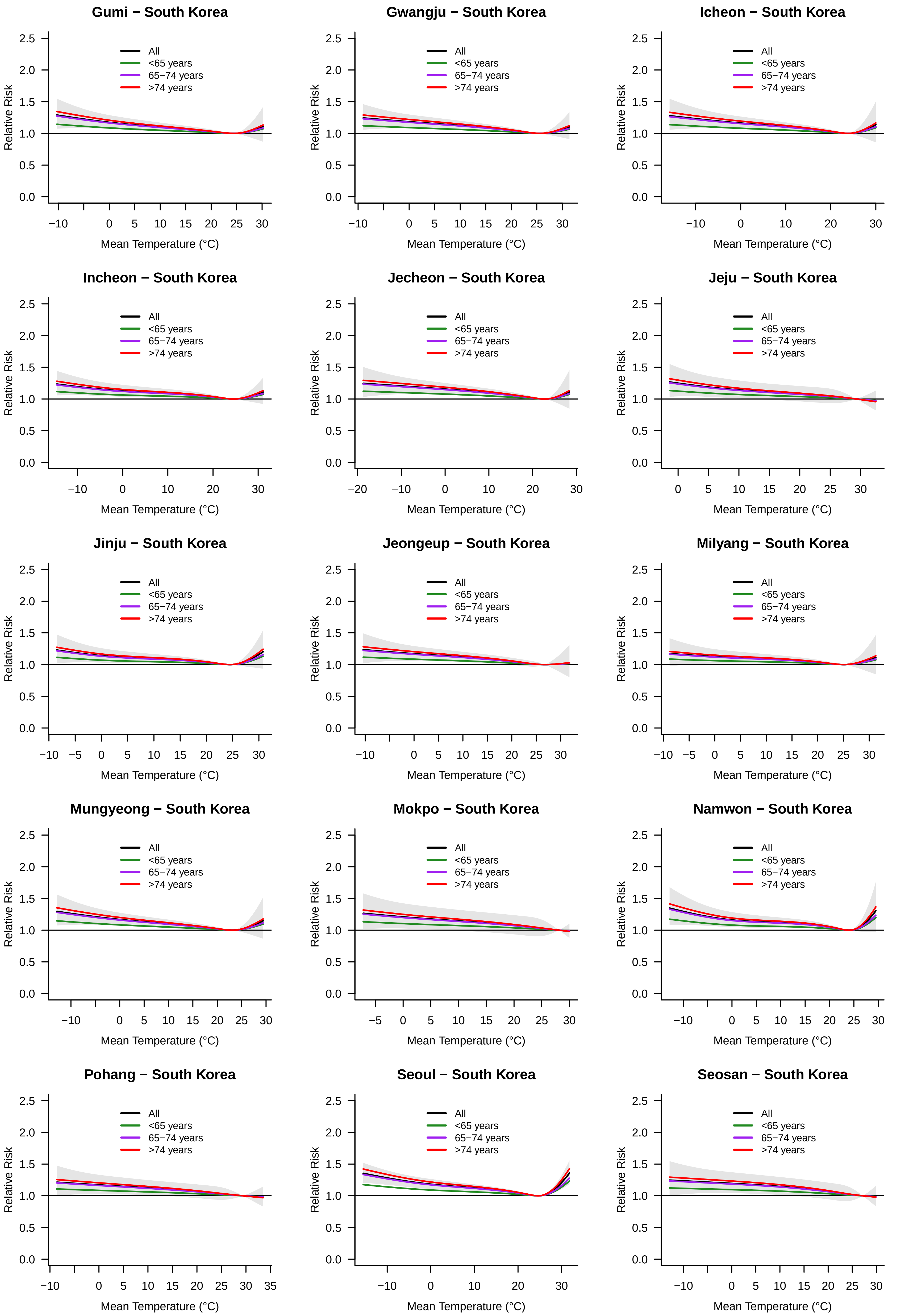
Gumi − South Korea
All
<65 years
65−74 years
>74 years
Relative Risk
0.0 0.5 1.0 1.5 2.0 2.5
−10 0 5 10 15 20 25 30
Mean Temperature (°C)
Gwangju − South Korea
All
<65 years
65−74 years
>74 years
Relative Risk
0.0 0.5 1.0 1.5 2.0 2.5
−10 0 5 10 15 20 25 30
Mean Temperature (°C)
Icheon − South Korea
All
<65 years
65−74 years
>74 years
Relative Risk
0.0 0.5 1.0 1.5 2.0 2.5
−10 0 10 20 30
Mean Temperature (°C)
Incheon − South Korea
All
<65 years
65−74 years
>74 years
Relative Risk
0.0 0.5 1.0 1.5 2.0 2.5
−10 0 10 20 30
Mean Temperature (°C)
Jecheon − South Korea
All
<65 years
65−74 years
>74 years
Relative Risk
0.0 0.5 1.0 1.5 2.0 2.5
−20 −10 0 10 20 30
Mean Temperature (°C)
Jeju − South Korea
All
<65 years
65−74 years
>74 years
Relative Risk
0.0 0.5 1.0 1.5 2.0 2.5
0 5 10 15 20 25 30
Mean Temperature (°C)
Jinju − South Korea
All
<65 years
65−74 years
>74 years
Relative Risk
0.0 0.5 1.0 1.5 2.0 2.5
−10 −5 0 5 10 15 20 25 30
Mean Temperature (°C)
Jeongeup − South Korea
All
<65 years
65−74 years
>74 years
Relative Risk
0.0 0.5 1.0 1.5 2.0 2.5
−10 0 5 10 15 20 25 30
Mean Temperature (°C)
Milyang − South Korea
All
<65 years
65−74 years
>74 years
Relative Risk
0.0 0.5 1.0 1.5 2.0 2.5
−10 −5 0 5 10 15 20 25 30
Mean Temperature (°C)
Mungyeong − South Korea
All
<65 years
65−74 years
>74 years
Relative Risk
0.0 0.5 1.0 1.5 2.0 2.5
−10 0 5 10 15 20 25 30
Mean Temperature (°C)
Mokpo − South Korea
All
<65 years
65−74 years
>74 years
Relative Risk
0.0 0.5 1.0 1.5 2.0 2.5
−5 0 5 10 15 20 25 30
Mean Temperature (°C)
Namwon − South Korea
All
<65 years
65−74 years
>74 years
Relative Risk
0.0 0.5 1.0 1.5 2.0 2.5
−10 0 5 10 15 20 25 30
Mean Temperature (°C)
Pohang − South Korea
All
<65 years
65−74 years
>74 years
Relative Risk
0.0 0.5 1.0 1.5 2.0 2.5
−10 0 5 10 15 20 25 30 35
Mean Temperature (°C)
Seoul − South Korea
All
<65 years
65−74 years
>74 years
Relative Risk
0.0 0.5 1.0 1.5 2.0 2.5
−10 0 10 20 30
Mean Temperature (°C)
Seosan − South Korea
All
<65 years
65−74 years
>74 years
Relative Risk
0.0 0.5 1.0 1.5 2.0 2.5
−10 0 5 10 15 20 25 30
Mean Temperature (°C)

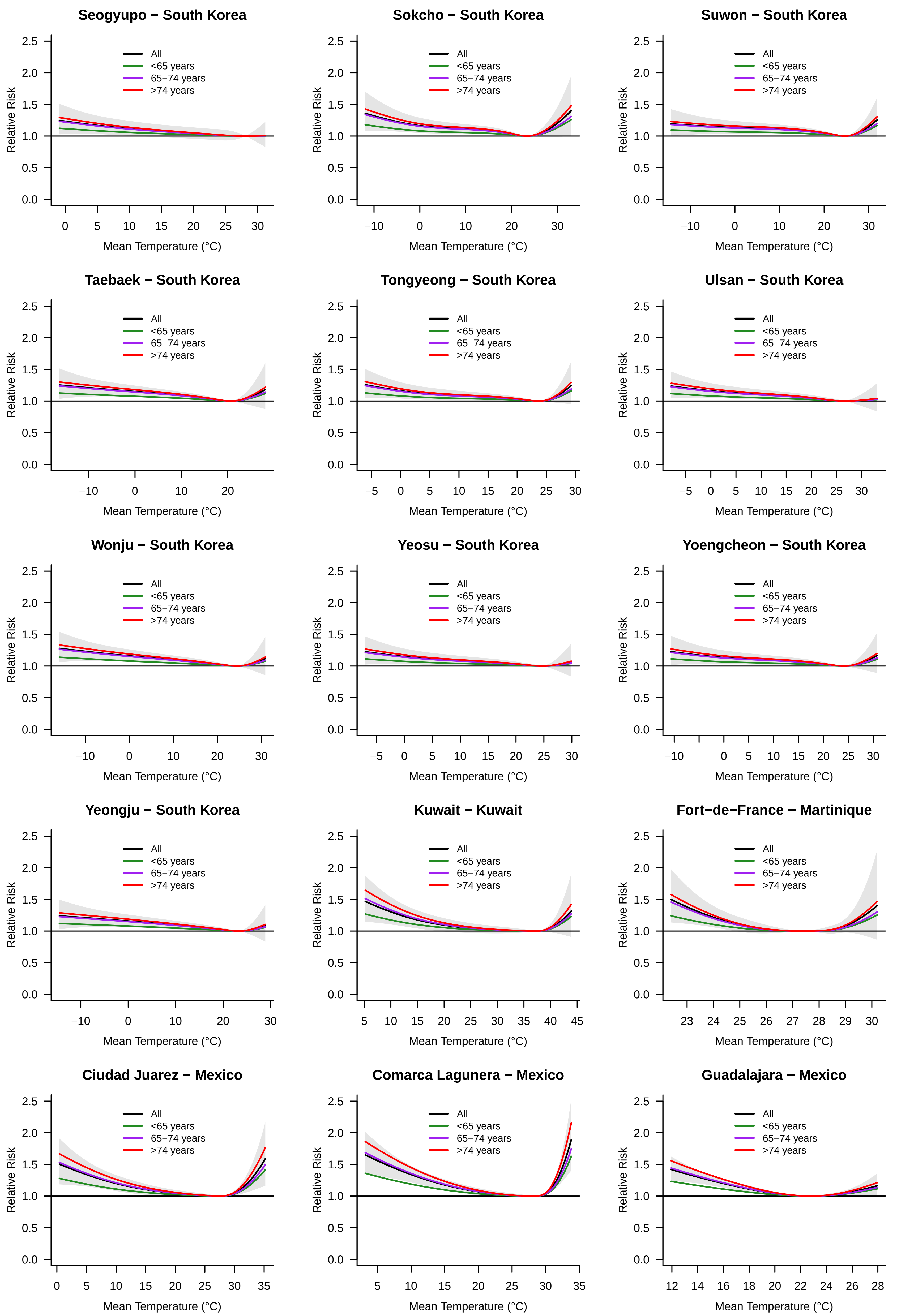
Seogyupo − South Korea
Sokcho − South Korea
Suwon − South Korea
Taebaek − South Korea
Tongyeong − South Korea
Ulsan − South Korea
Wonju − South Korea
Yeosu − South Korea
Yoengcheon − South Korea
Yeongju − South Korea
Kuwait − Kuwait
Fort−de−France − Martinique
Ciudad Juarez − Mexico
Comarca Lagunera − Mexico
Guadalajara − Mexico
All
<65 years
65−74 years
>74 years
Relative Risk
0.0
0.5
1.0
1.5
2.0
2.5
Mean Temperature (°C)
0 5 10 15 20 25 30
−10 0 10 20 30
−10 0 10 20 30
−10 0 10 20
−5 0 5 10 15 20 25 30
−5 0 5 10 15 20 25 30
−10 0 10 20 30
−5 0 5 10 15 20 25 30
−10 0 5 10 15 20 25 30
−10 0 10 20 30
5 10 15 20 25 30 35 40 45
23 24 25 26 27 28 29 30
0 5 10 15 20 25 30 35
5 10 15 20 25 30 35
12 14 16 18 20 22 24 26 28

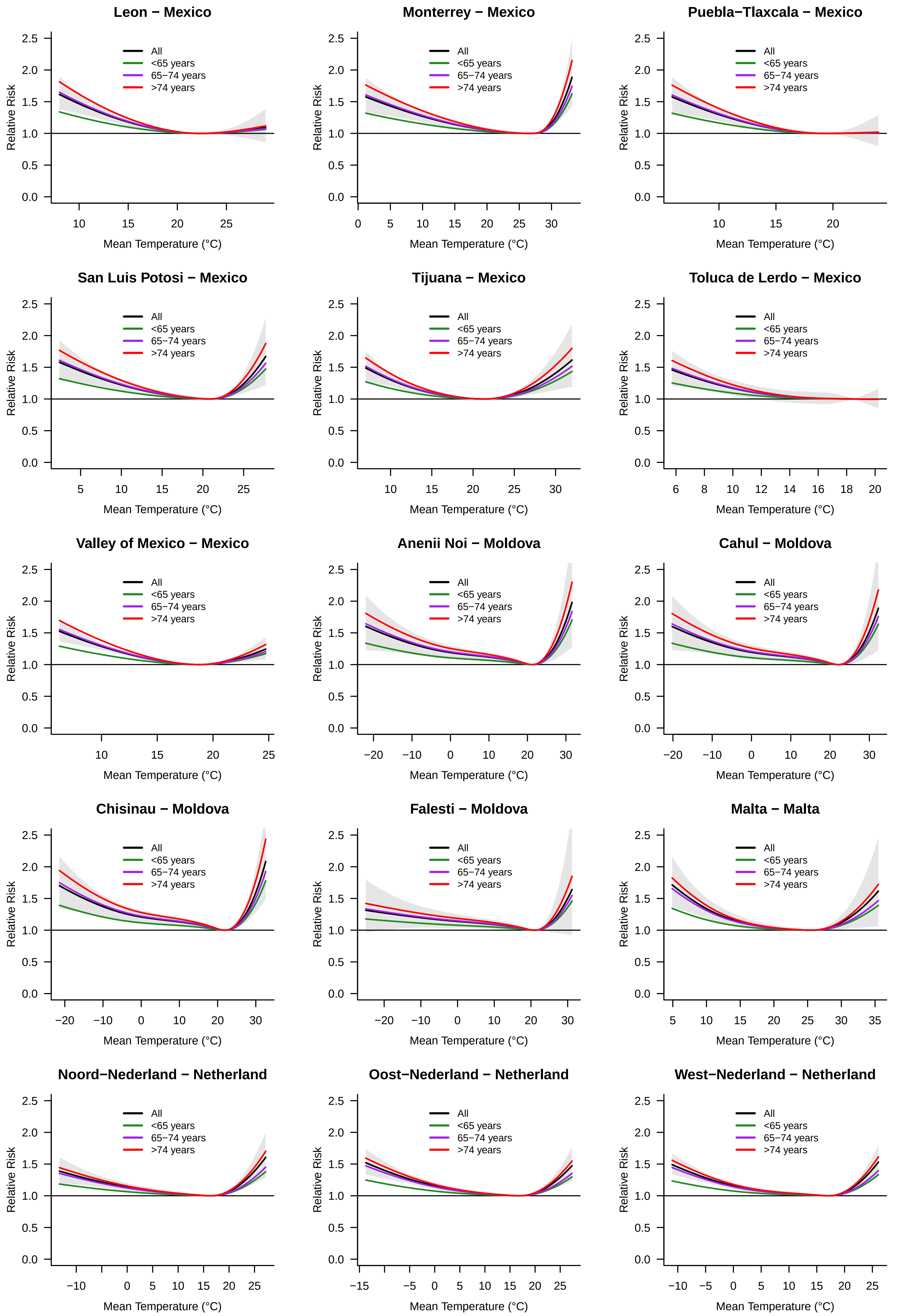
Leon − Mexico
Monterrey − Mexico
Puebla−Tlaxcala − Mexico
San Luis Potosi − Mexico
Tijuana − Mexico
Toluca de Lerdo − Mexico
Valley of Mexico − Mexico
Anenii Noi − Moldova
Cahul − Moldova
Chisinau − Moldova
Falesti − Moldova
Malta − Malta
Noord−Nederland − Netherland
Oost−Nederland − Netherland
West−Nederland − Netherland
All
<65 years
65−74 years
>74 years
Relative Risk
Mean Temperature (°C)
0.0
0.5
1.0
1.5
2.0
2.5

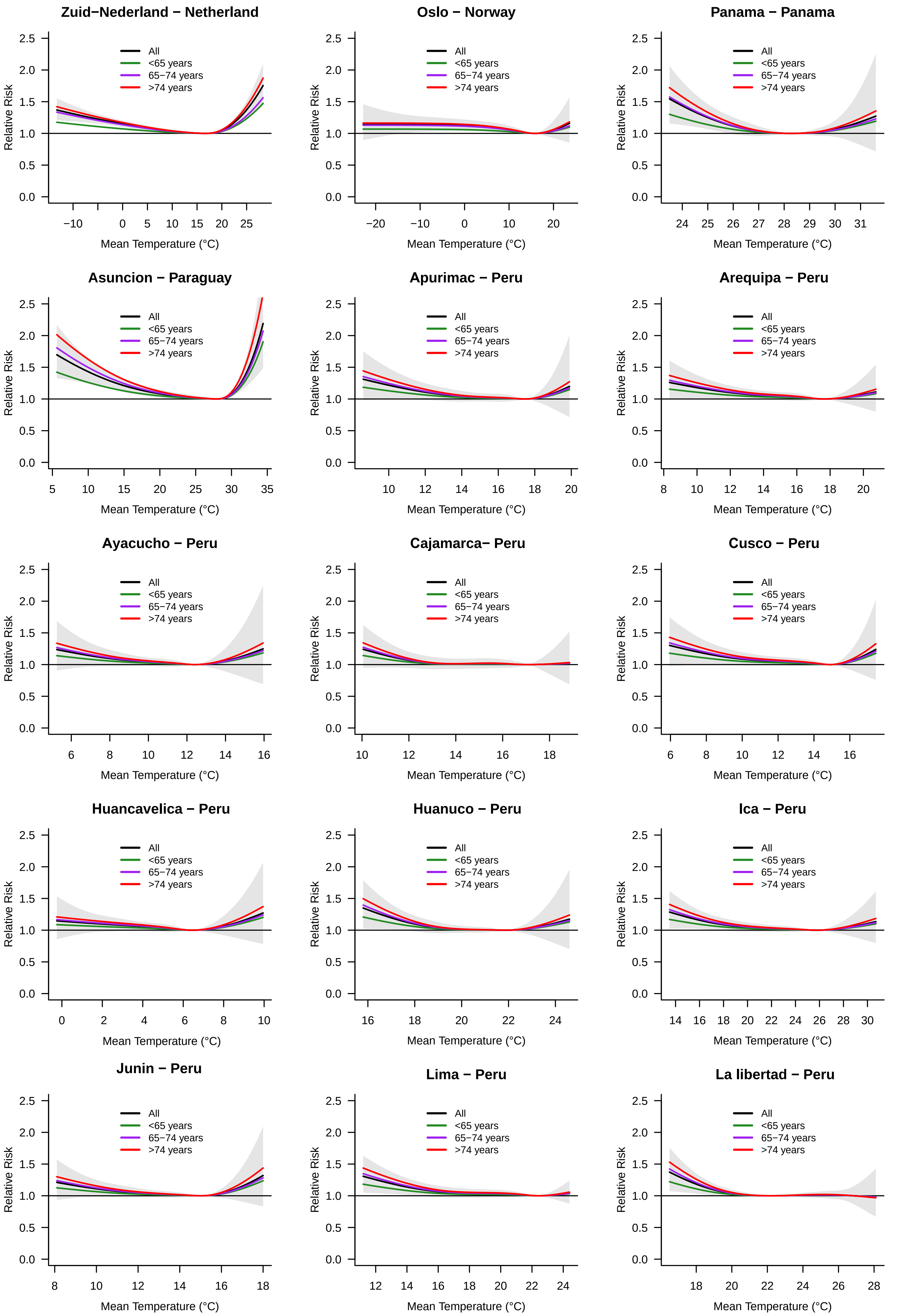
Zuid−Nederland − Netherland
Oslo − Norway
Panama − Panama
Asuncion − Paraguay
Apurimac − Peru
Arequipa − Peru
Ayacucho − Peru
Cajamarca− Peru
Cusco − Peru
Huancavelica − Peru
Huanuco − Peru
Ica − Peru
Junin − Peru
Lima − Peru
La libertad − Peru
All
<65 years
65−74 years
>74 years
Relative Risk
Mean Temperature (°C)

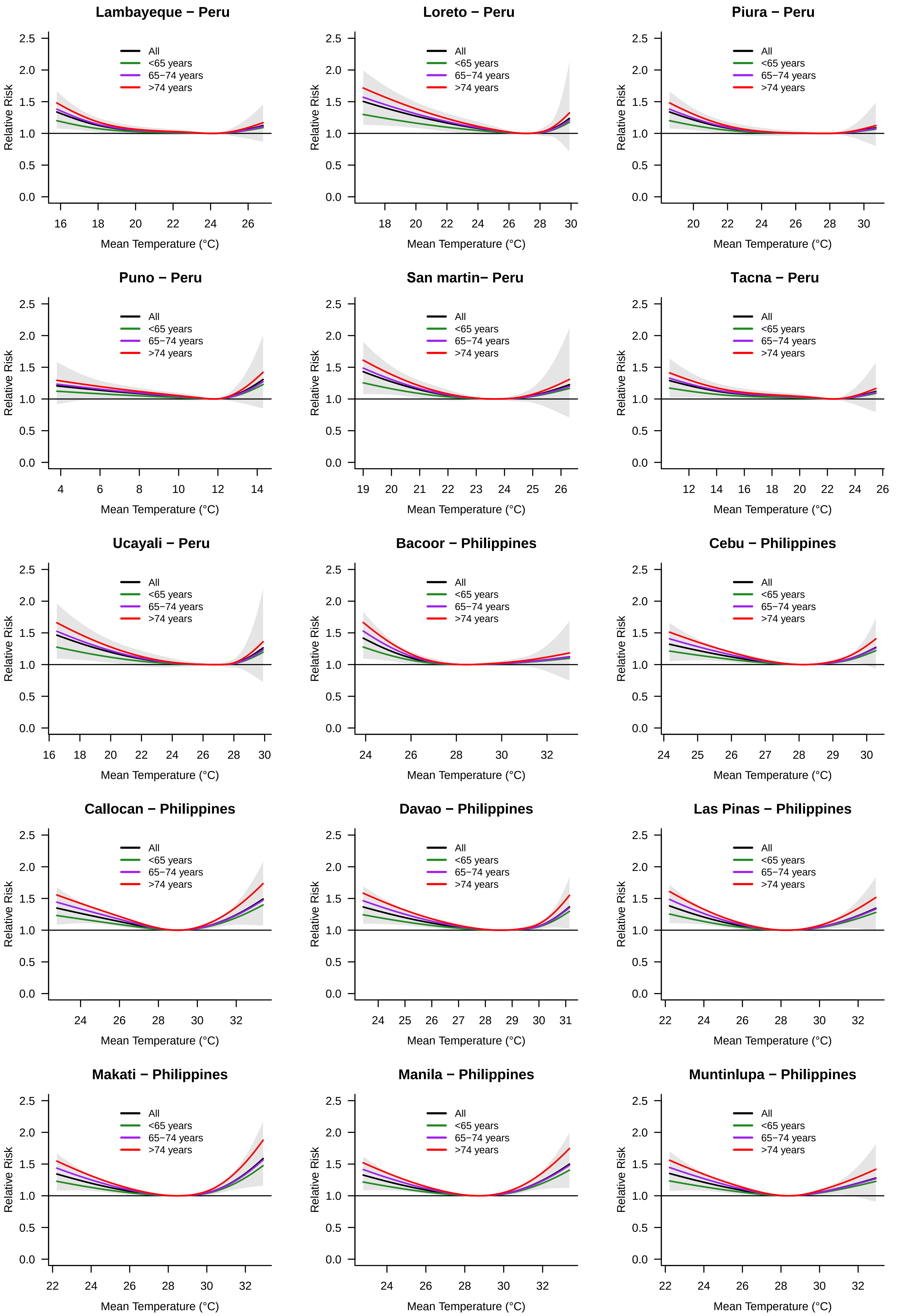
Lambayeque − Peru
Loreto − Peru
Piura − Peru
Puno − Peru
San martin− Peru
Tacna − Peru
Ucayali − Peru
Bacoor − Philippines
Cebu − Philippines
Callocan − Philippines
Davao − Philippines
Las Pinas − Philippines
Makati − Philippines
Manila − Philippines
Muntinlupa − Philippines
All
<65 years
65−74 years
>74 years
Relative Risk
Mean Temperature (°C)

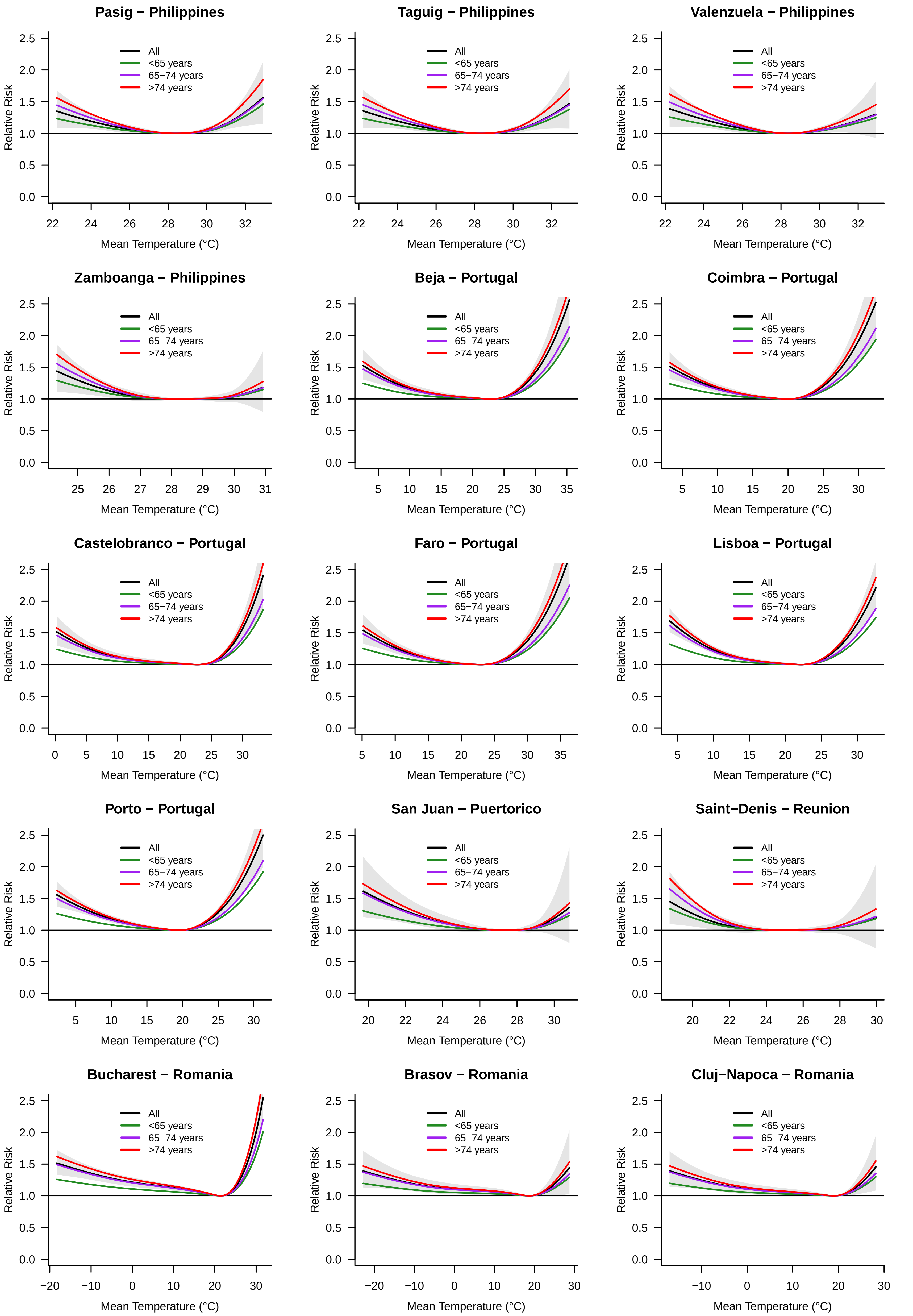
Pasig − Philippines
Taguig − Philippines
Valenzuela − Philippines
Zamboanga − Philippines
Beja − Portugal
Coimbra − Portugal
Castelobranco − Portugal
Faro − Portugal
Lisboa − Portugal
Porto − Portugal
San Juan − Puertorico
Saint−Denis − Reunion
Bucharest − Romania
Brasov − Romania
Cluj−Napoca − Romania
All
<65 years
65−74 years
>74 years
Relative Risk
Mean Temperature (°C)

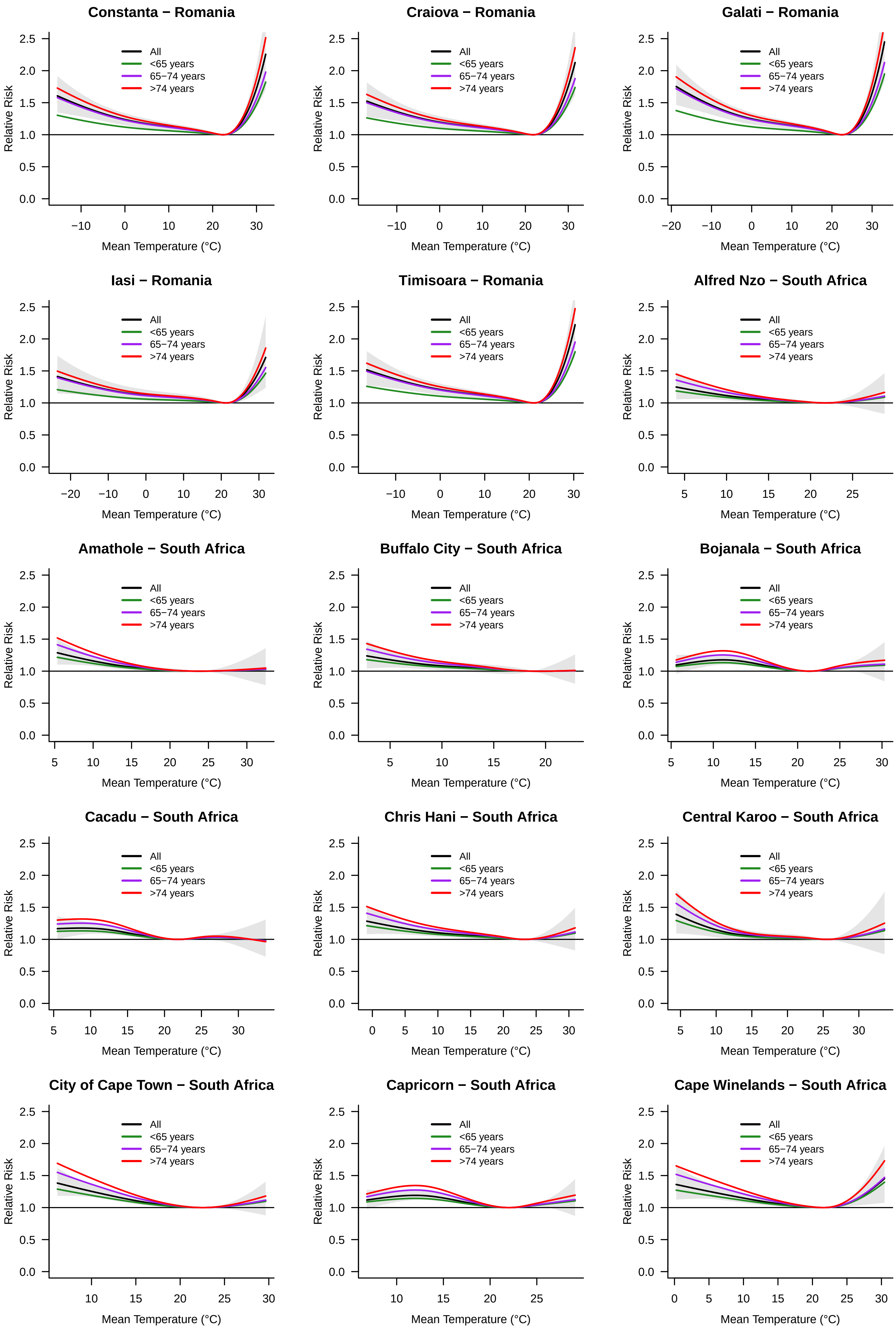

Constanta − Romania
Craiova − Romania
Galati − Romania
Iasi − Romania
Timisoara − Romania
Alfred Nzo − South Africa
Amathole − South Africa
Buffalo City − South Africa
Bojanala − South Africa
Cacadu − South Africa
Chris Hani − South Africa
Central Karoo − South Africa
City of Cape Town − South Africa
Capricorn − South Africa
Cape Winelands − South Africa
All
<65 years
65−74 years
>74 years
Relative Risk
Mean Temperature (°C)

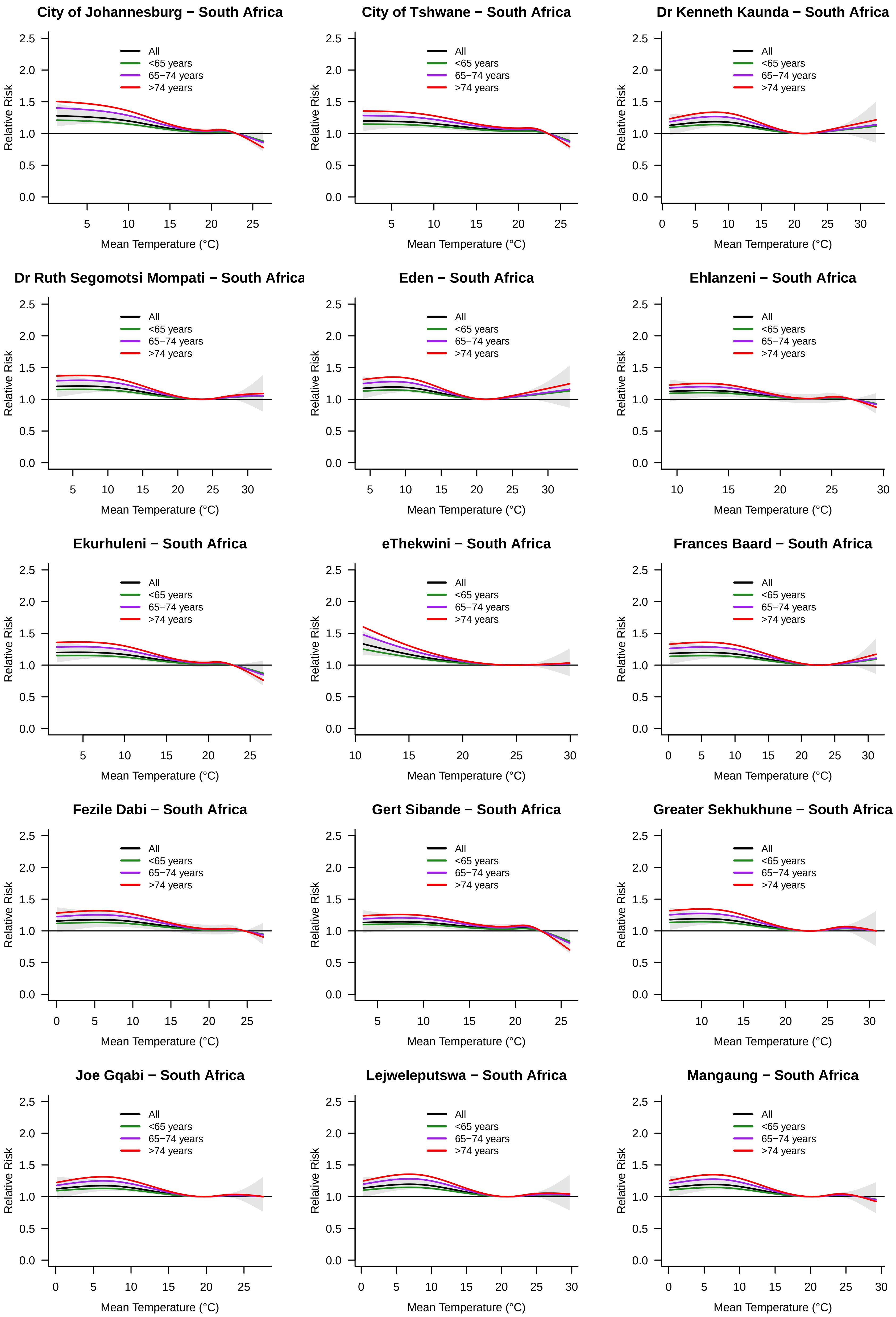
City of Johannesburg − South Africa
City of Tshwane − South Africa
Dr Kenneth Kaunda − South Africa
Dr Ruth Segomotsi Mompati − South Africa
Eden − South Africa
Ehlanzeni − South Africa
Ekurhuleni − South Africa
eThekwini − South Africa
Frances Baard − South Africa
Fezile Dabi − South Africa
Gert Sibande − South Africa
Greater Sekhukhune − South Africa
Joe Gqabi − South Africa
Lejweleputswa − South Africa
Mangaung − South Africa
All
<65 years
65−74 years
>74 years
Relative Risk
Mean Temperature (°C)

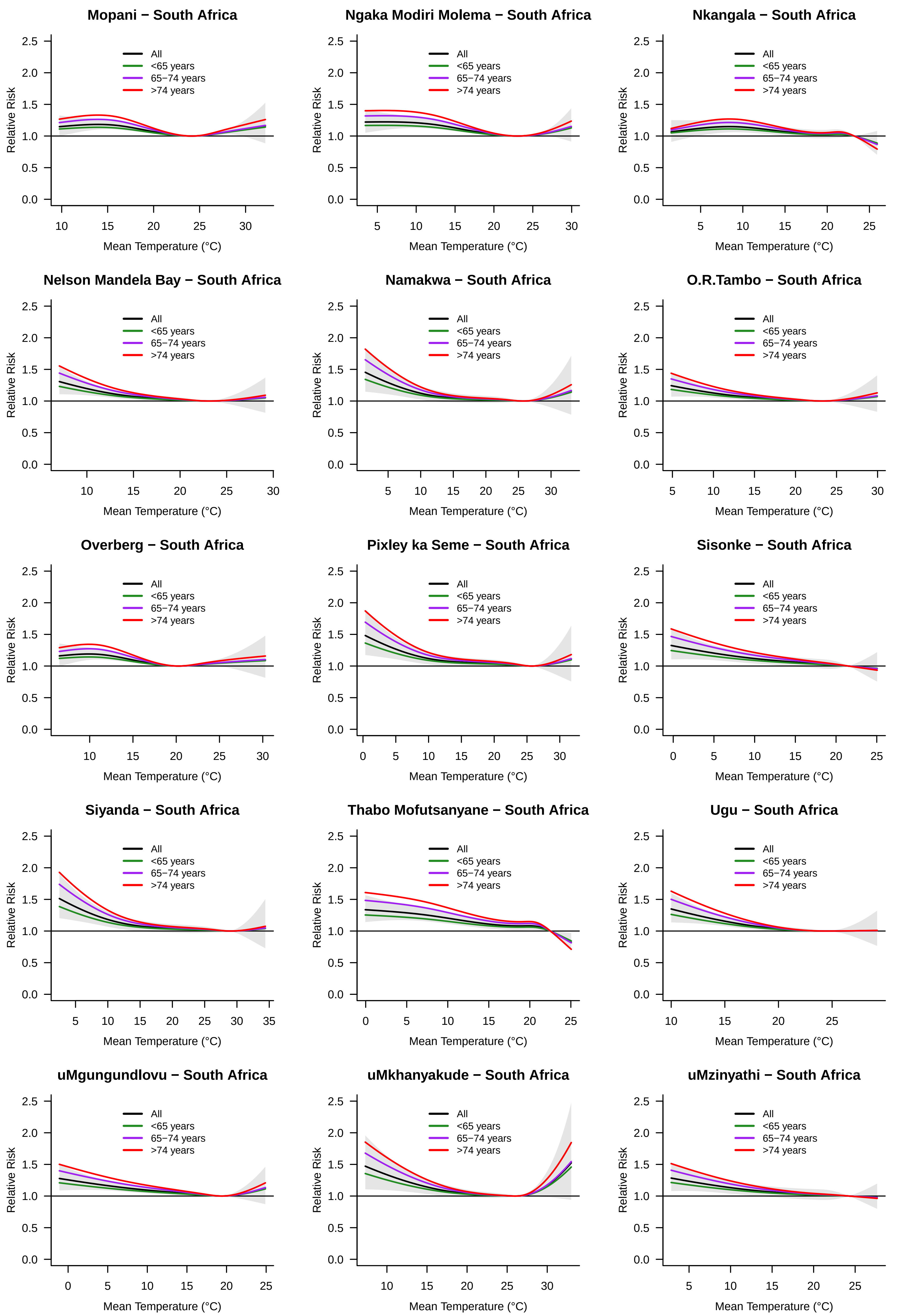
Mopani − South Africa
Ngaka Modiri Molema − South Africa
Nkangala − South Africa
Nelson Mandela Bay − South Africa
Namakwa − South Africa
O.R.Tambo − South Africa
Overberg − South Africa
Pixley ka Seme − South Africa
Sisonke − South Africa
Siyanda − South Africa
Thabo Mofutsanyane − South Africa
Ugu − South Africa
uMgungundlovu − South Africa
uMkhanyakude − South Africa
uMzinyathi − South Africa
All
<65 years
65−74 years
>74 years
Relative Risk
Mean Temperature (°C)

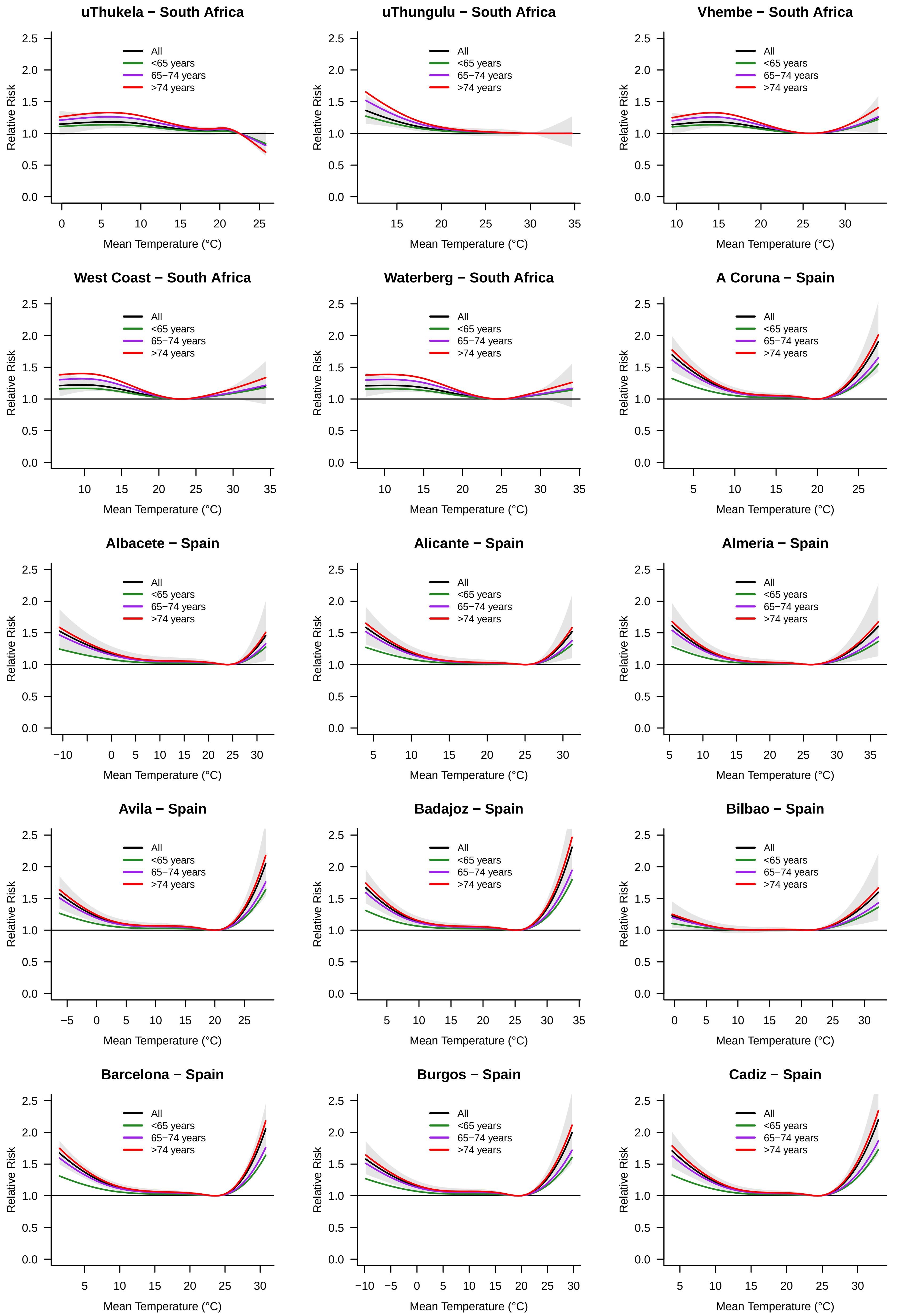
uThukela − South Africa
uThungulu − South Africa
Vhembe − South Africa
West Coast − South Africa
Waterberg − South Africa
A Coruna − Spain
Albacete − Spain
Alicante − Spain
Almeria − Spain
Avila − Spain
Badajoz − Spain
Bilbao − Spain
Barcelona − Spain
Burgos − Spain
Cadiz − Spain
All
<65 years
65−74 years
>74 years
Relative Risk
0.0
0.5
1.0
1.5
2.0
2.5
Mean Temperature (°C)
0 5 10 15 20 25
15 20 25 30 35
10 15 20 25 30
10 15 20 25 30 35
10 15 20 25 30 35
5 10 15 20 25
−10 0 5 10 15 20 25 30
5 10 15 20 25 30
5 10 15 20 25 30 35
−5 0 5 10 15 20 25
5 10 15 20 25 30 35
0 5 10 15 20 25 30
5 10 15 20 25 30
−10 −5 0 5 10 15 20 25 30
5 10 15 20 25 30

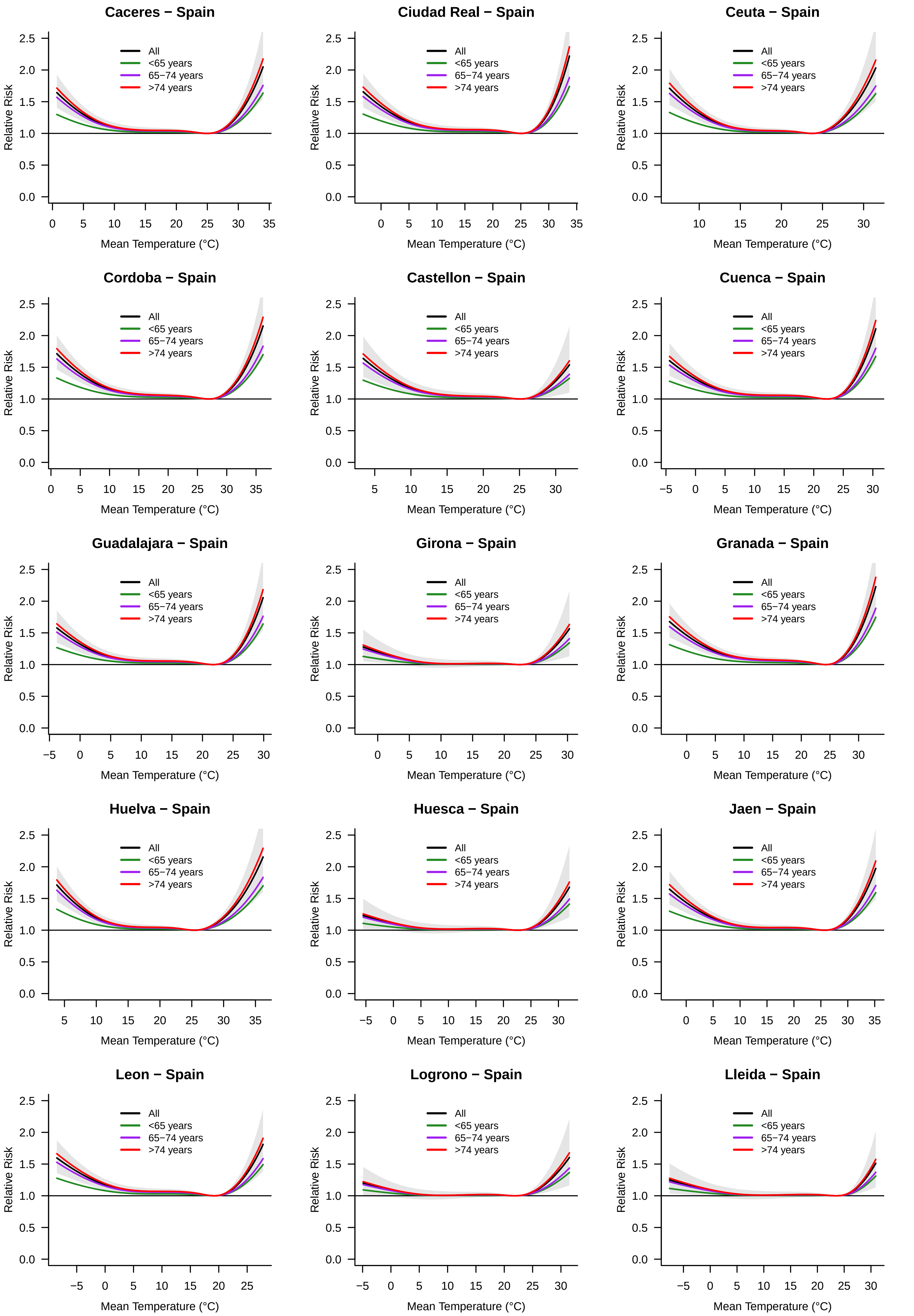
Caceres − Spain
Ciudad Real − Spain
Ceuta − Spain
Cordoba − Spain
Castellon − Spain
Cuenca − Spain
Guadalajara − Spain
Girona − Spain
Granada − Spain
Huelva − Spain
Huesca − Spain
Jaen − Spain
Leon − Spain
Logrono − Spain
Lleida − Spain
All
<65 years
65−74 years
>74 years
Relative Risk
Mean Temperature (°C)

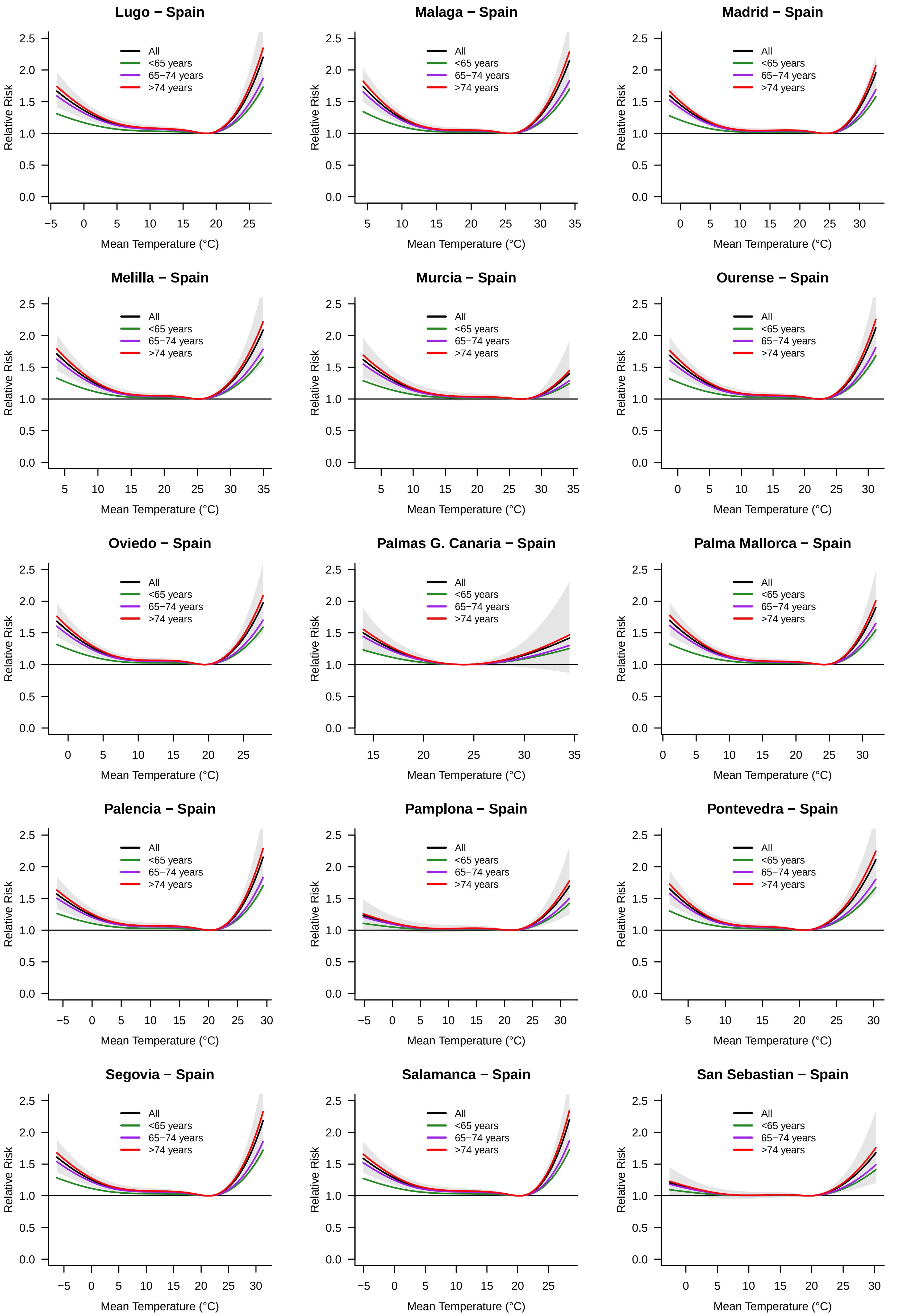

Lugo − Spain
Malaga − Spain
Madrid − Spain
Melilla − Spain
Murcia − Spain
Ourense − Spain
Oviedo − Spain
Palmas G. Canaria − Spain
Palma Mallorca − Spain
Palencia − Spain
Pamplona − Spain
Pontevedra − Spain
Segovia − Spain
Salamanca − Spain
San Sebastian − Spain
All
<65 years
65−74 years
>74 years
Relative Risk
Mean Temperature (°C)

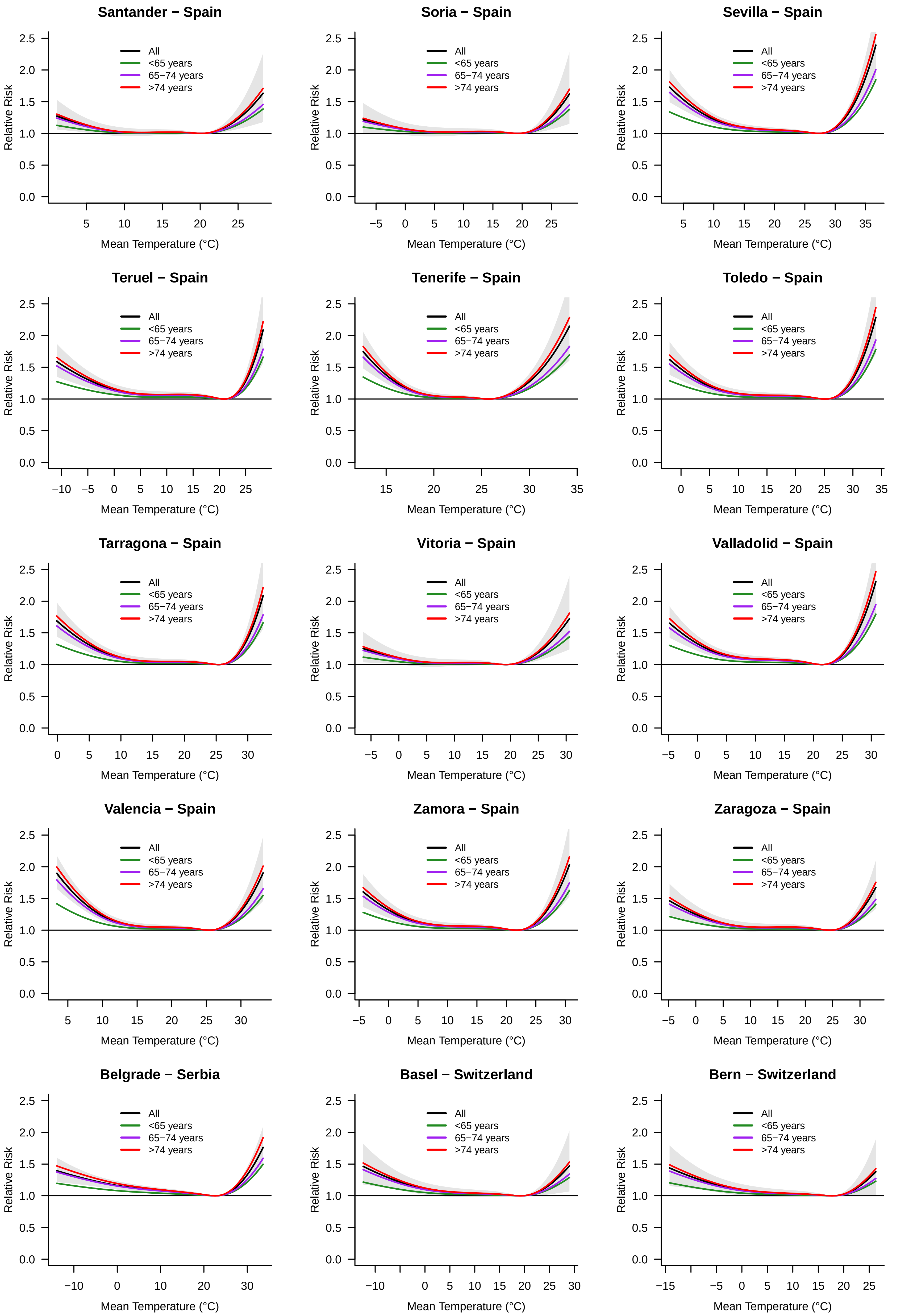
Santander − Spain
Soria − Spain
Sevilla − Spain
Teruel − Spain
Tenerife − Spain
Toledo − Spain
Tarragona − Spain
Vitoria − Spain
Valladolid − Spain
Valencia − Spain
Zamora − Spain
Zaragoza − Spain
Belgrade − Serbia
Basel − Switzerland
Bern − Switzerland
All
<65 years
65−74 years
>74 years
Relative Risk
Mean Temperature (°C)

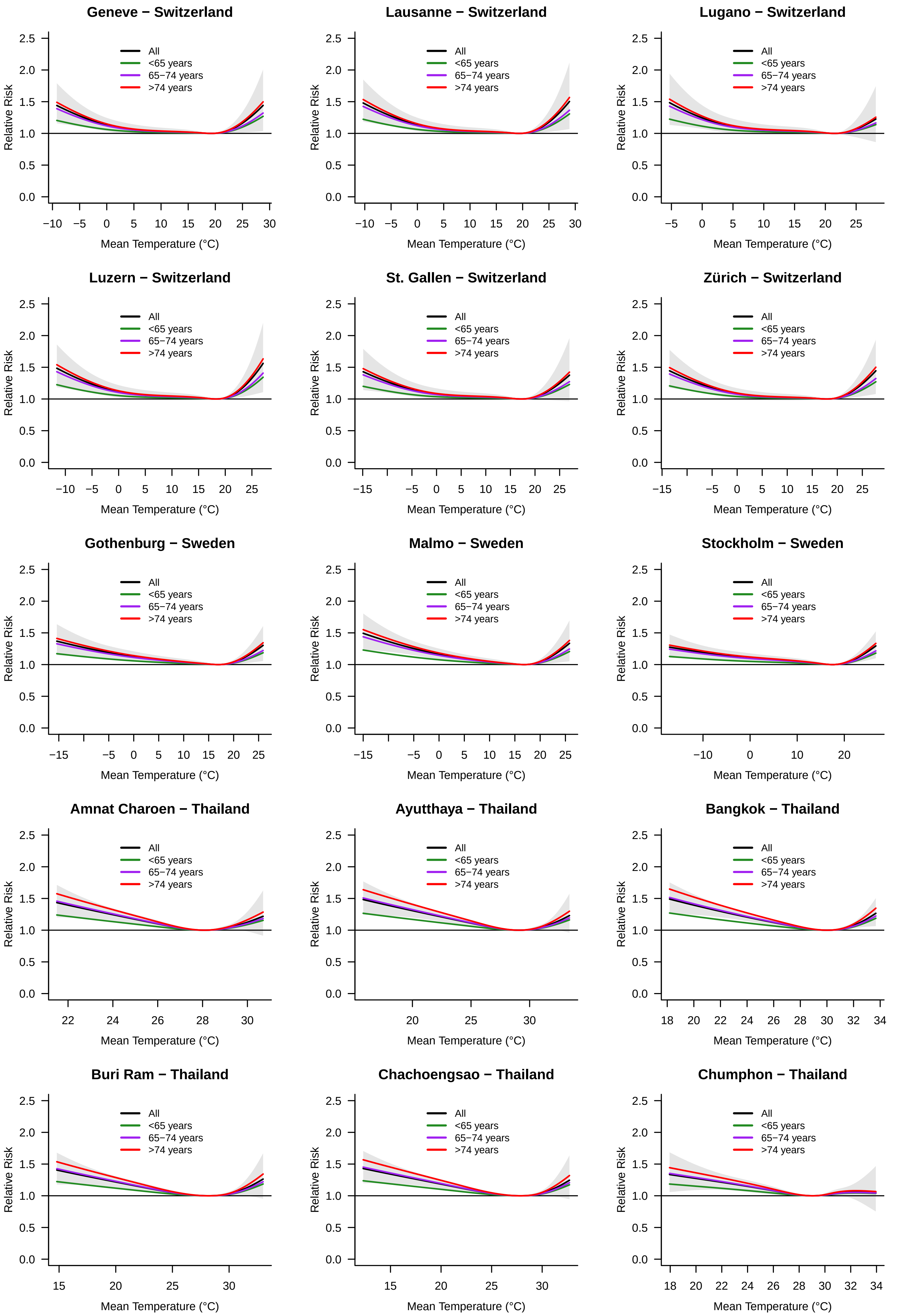
Geneve − Switzerland
All
<65 years
65−74 years
>74 years
Relative Risk
0.0 0.5 1.0 1.5 2.0 2.5
−10 −5 0 5 10 15 20 25 30
Mean Temperature (°C)
Lausanne − Switzerland
All
<65 years
65−74 years
>74 years
Relative Risk
0.0 0.5 1.0 1.5 2.0 2.5
−10 −5 0 5 10 15 20 25 30
Mean Temperature (°C)
Lugano − Switzerland
All
<65 years
65−74 years
>74 years
Relative Risk
0.0 0.5 1.0 1.5 2.0 2.5
−5 0 5 10 15 20 25
Mean Temperature (°C)
Luzern − Switzerland
All
<65 years
65−74 years
>74 years
Relative Risk
0.0 0.5 1.0 1.5 2.0 2.5
−10 −5 0 5 10 15 20 25
Mean Temperature (°C)
St. Gallen − Switzerland
All
<65 years
65−74 years
>74 years
Relative Risk
0.0 0.5 1.0 1.5 2.0 2.5
−15 −5 0 5 10 15 20 25
Mean Temperature (°C)
Zürich − Switzerland
All
<65 years
65−74 years
>74 years
Relative Risk
0.0 0.5 1.0 1.5 2.0 2.5
−15 −5 0 5 10 15 20 25
Mean Temperature (°C)
Gothenburg − Sweden
All
<65 years
65−74 years
>74 years
Relative Risk
0.0 0.5 1.0 1.5 2.0 2.5
−15 −5 0 5 10 15 20 25
Mean Temperature (°C)
Malmo − Sweden
All
<65 years
65−74 years
>74 years
Relative Risk
0.0 0.5 1.0 1.5 2.0 2.5
−15 −5 0 5 10 15 20 25
Mean Temperature (°C)
Stockholm − Sweden
All
<65 years
65−74 years
>74 years
Relative Risk
0.0 0.5 1.0 1.5 2.0 2.5
−10 0 10 20
Mean Temperature (°C)
Amnat Charoen − Thailand
All
<65 years
65−74 years
>74 years
Relative Risk
0.0 0.5 1.0 1.5 2.0 2.5
22 24 26 28 30
Mean Temperature (°C)
Ayutthaya − Thailand
All
<65 years
65−74 years
>74 years
Relative Risk
0.0 0.5 1.0 1.5 2.0 2.5
20 25 30
Mean Temperature (°C)
Bangkok − Thailand
All
<65 years
65−74 years
>74 years
Relative Risk
0.0 0.5 1.0 1.5 2.0 2.5
18 20 22 24 26 28 30 32 34
Mean Temperature (°C)
Buri Ram − Thailand
All
<65 years
65−74 years
>74 years
Relative Risk
0.0 0.5 1.0 1.5 2.0 2.5
15 20 25 30
Mean Temperature (°C)
Chachoengsao − Thailand
All
<65 years
65−74 years
>74 years
Relative Risk
0.0 0.5 1.0 1.5 2.0 2.5
15 20 25 30
Mean Temperature (°C)
Chumphon − Thailand
All
<65 years
65−74 years
>74 years
Relative Risk
0.0 0.5 1.0 1.5 2.0 2.5
18 20 22 24 26 28 30 32 34
Mean Temperature (°C)

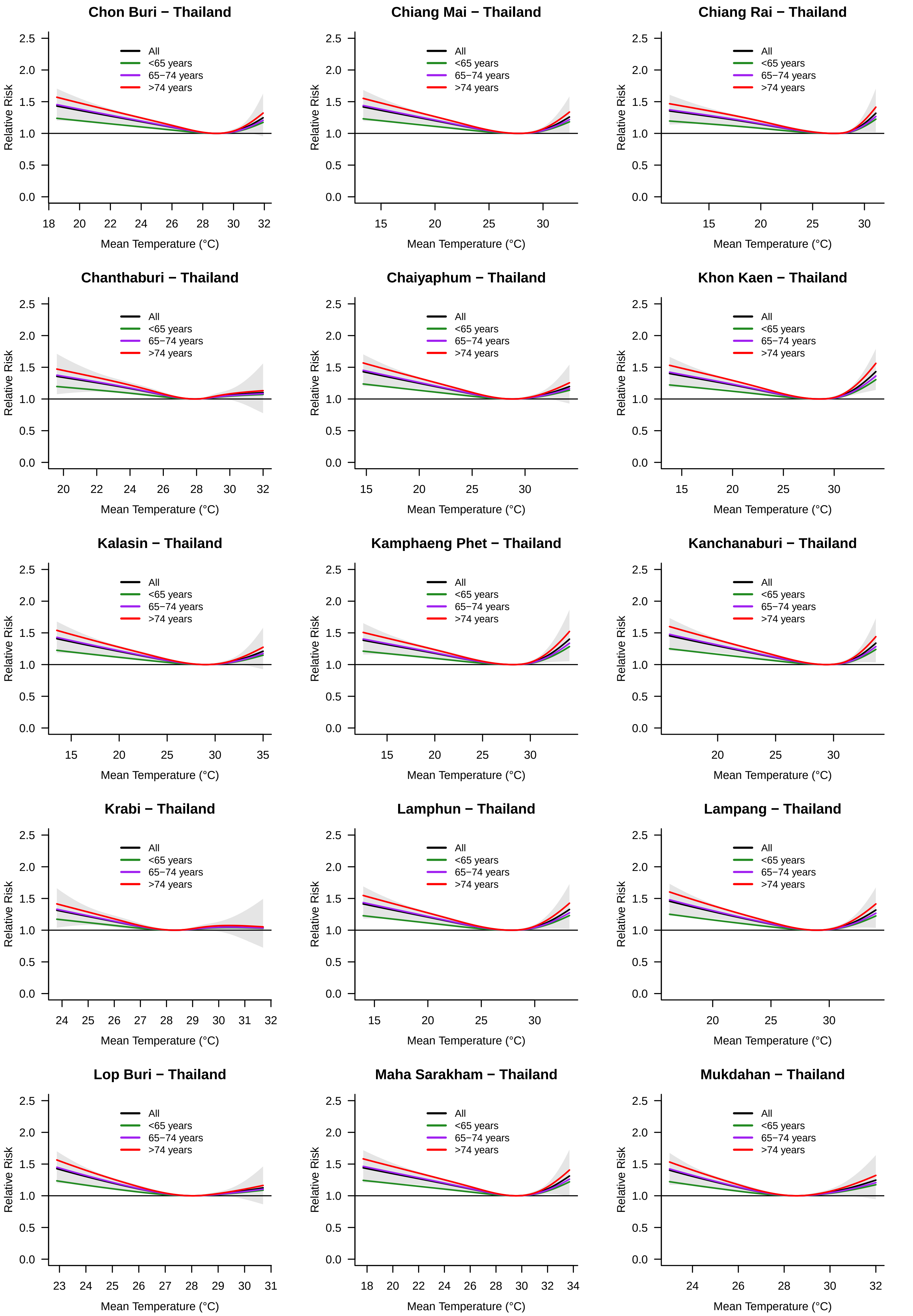
Chon Buri − Thailand
Chiang Mai − Thailand
Chiang Rai − Thailand
Chanthaburi − Thailand
Chaiyaphum − Thailand
Khon Kaen − Thailand
Kalasin − Thailand
Kamphaeng Phet − Thailand
Kanchanaburi − Thailand
Krabi − Thailand
Lamphun − Thailand
Lampang − Thailand
Lop Buri − Thailand
Maha Sarakham − Thailand
Mukdahan − Thailand
All
<65 years
65−74 years
>74 years
Relative Risk
0.0
0.5
1.0
1.5
2.0
2.5
Mean Temperature (°C)
18 20 22 24 26 28 30 32
15 20 25 30
15 20 25 30
20 22 24 26 28 30 32
15 20 25 30
15 20 25 30
15 20 25 30 35
15 20 25 30
20 25 30
24 25 26 27 28 29 30 31 32
15 20 25 30
20 25 30
23 24 25 26 27 28 29 30 31
18 20 22 24 26 28 30 32 34
24 26 28 30 32

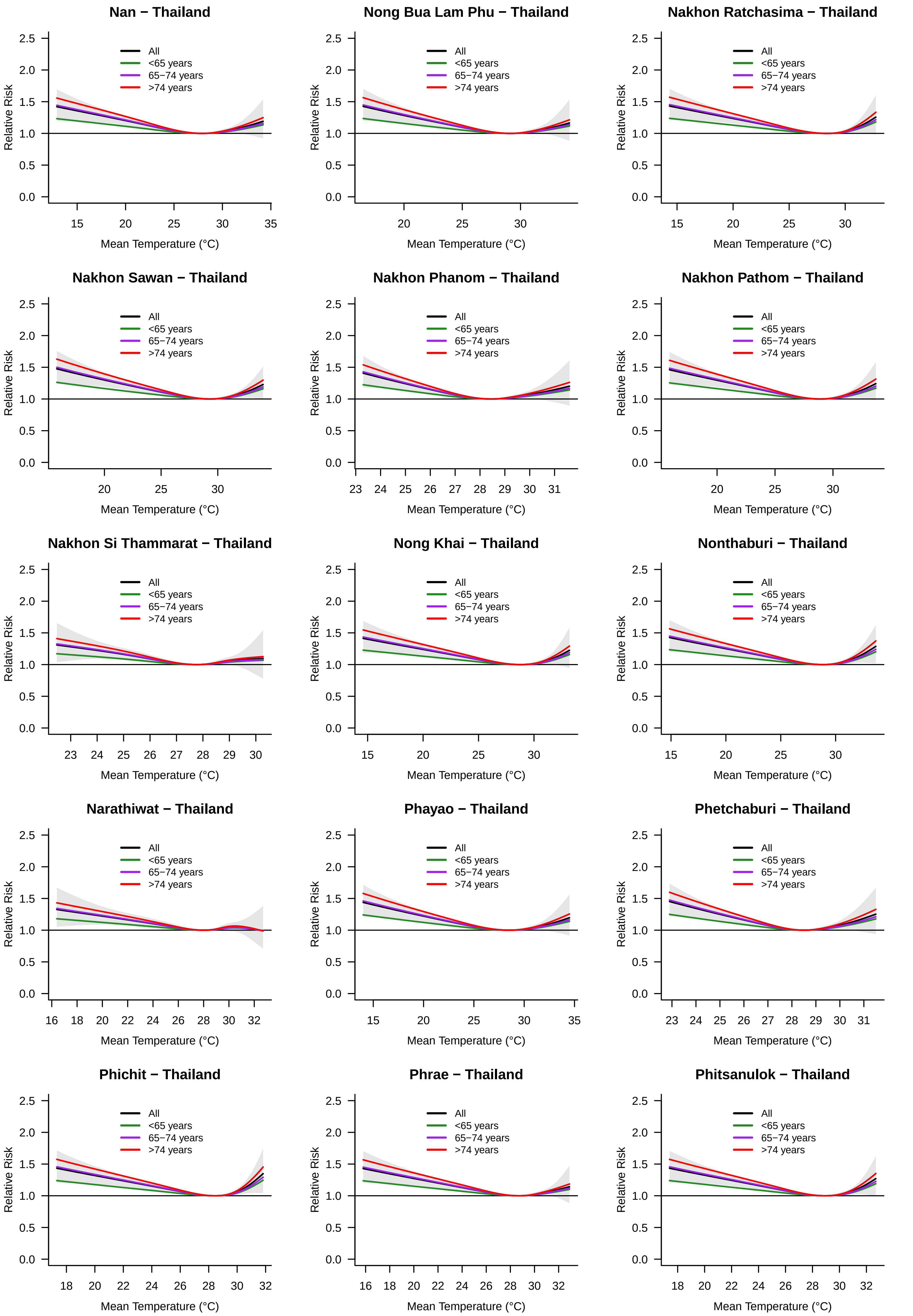
Nan − Thailand
Nong Bua Lam Phu − Thailand
Nakhon Ratchasima − Thailand
Nakhon Sawan − Thailand
Nakhon Phanom − Thailand
Nakhon Pathom − Thailand
Nakhon Si Thammarat − Thailand
Nong Khai − Thailand
Nonthaburi − Thailand
Narathiwat − Thailand
Phayao − Thailand
Phetchaburi − Thailand
Phichit − Thailand
Phrae − Thailand
Phitsanulok − Thailand
All
<65 years
65−74 years
>74 years
Relative Risk
0.0
0.5
1.0
1.5
2.0
2.5
Mean Temperature (°C)

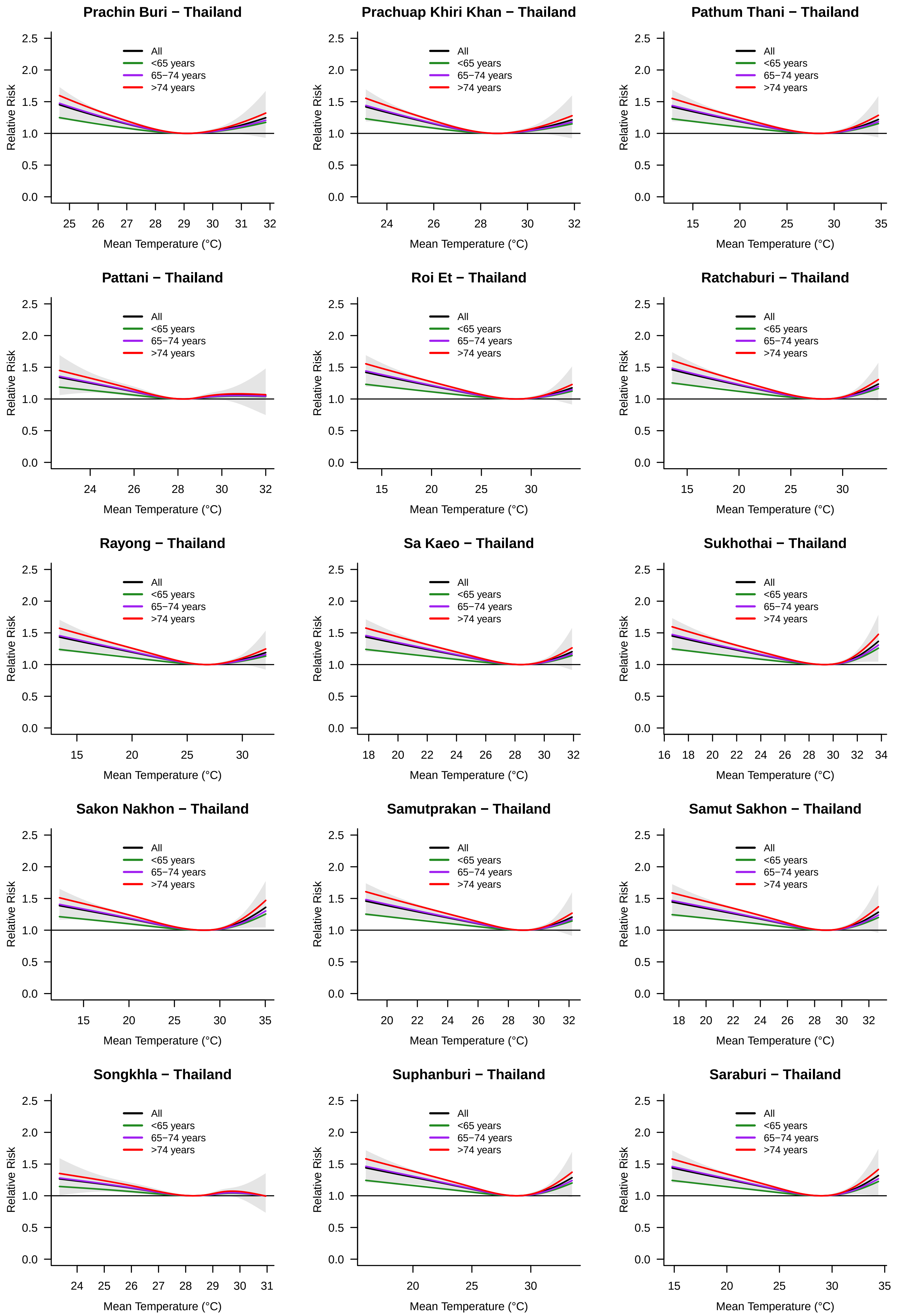
Prachin Buri − Thailand
Prachuap Khiri Khan − Thailand
Pathum Thani − Thailand
Pattani − Thailand
Roi Et − Thailand
Ratchaburi − Thailand
Rayong − Thailand
Sa Kaeo − Thailand
Sukhothai − Thailand
Sakon Nakhon − Thailand
Samutprakan − Thailand
Samut Sakhon − Thailand
Songkhla − Thailand
Suphanburi − Thailand
Saraburi − Thailand
All
<65 years
65−74 years
>74 years
Relative Risk
0.0 0.5 1.0 1.5 2.0 2.5
Mean Temperature (°C)
25 26 27 28 29 30 31 32
24 26 28 30 32
15 20 25 30 35
24 26 28 30 32
15 20 25 30
15 20 25 30
15 20 25 30
18 20 22 24 26 28 30 32
16 18 20 22 24 26 28 30 32 34
15 20 25 30 35
20 22 24 26 28 30 32
18 20 22 24 26 28 30 32
24 25 26 27 28 29 30 31
20 25 30
15 20 25 30 35

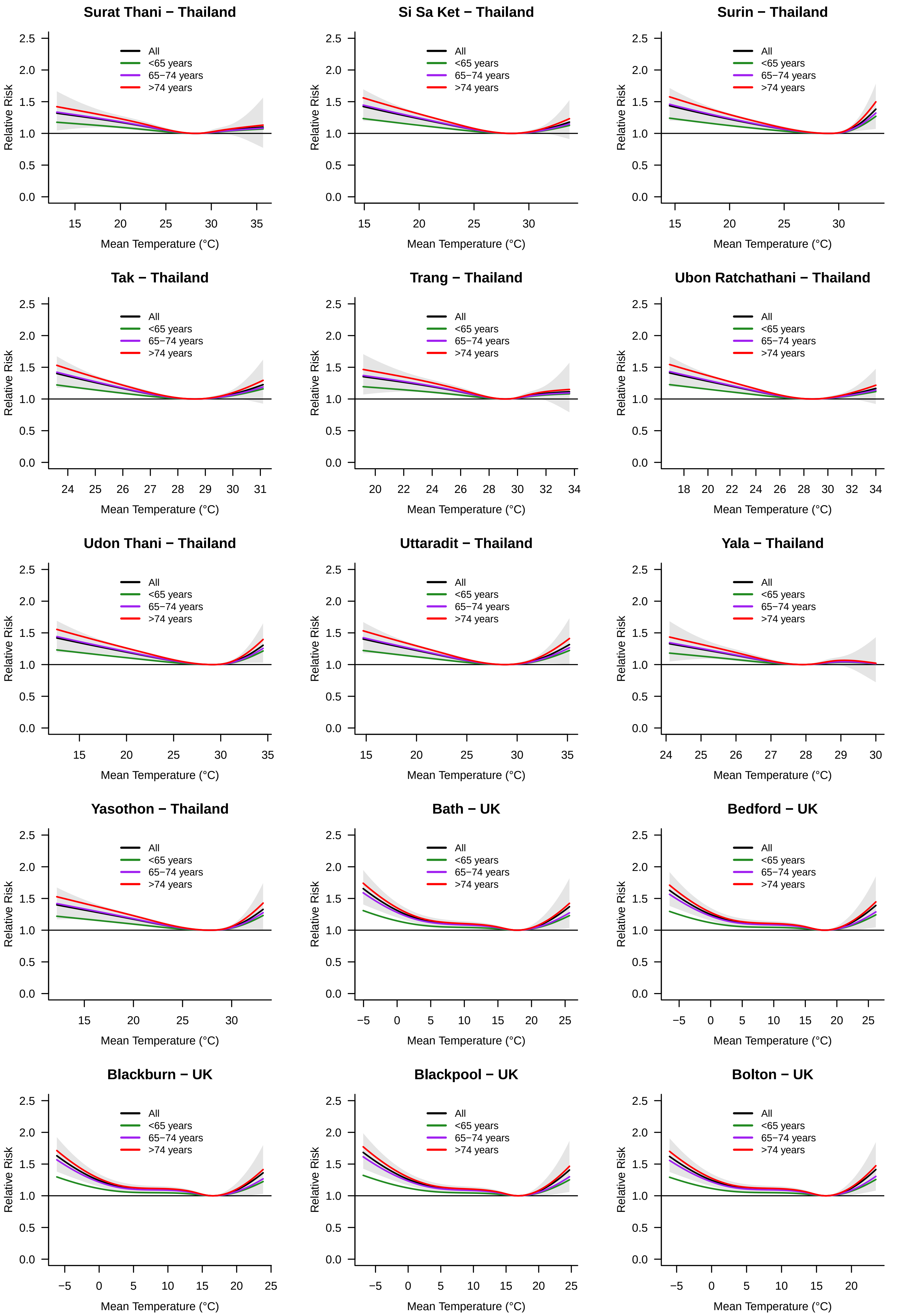

Surat Thani − Thailand
Si Sa Ket − Thailand
Surin − Thailand
Tak − Thailand
Trang − Thailand
Ubon Ratchathani − Thailand
Udon Thani − Thailand
Uttaradit − Thailand
Yala − Thailand
Yasothon − Thailand
Bath − UK
Bedford − UK
Blackburn − UK
Blackpool − UK
Bolton − UK
All
<65 years
65−74 years
>74 years
Relative Risk
Mean Temperature (°C)

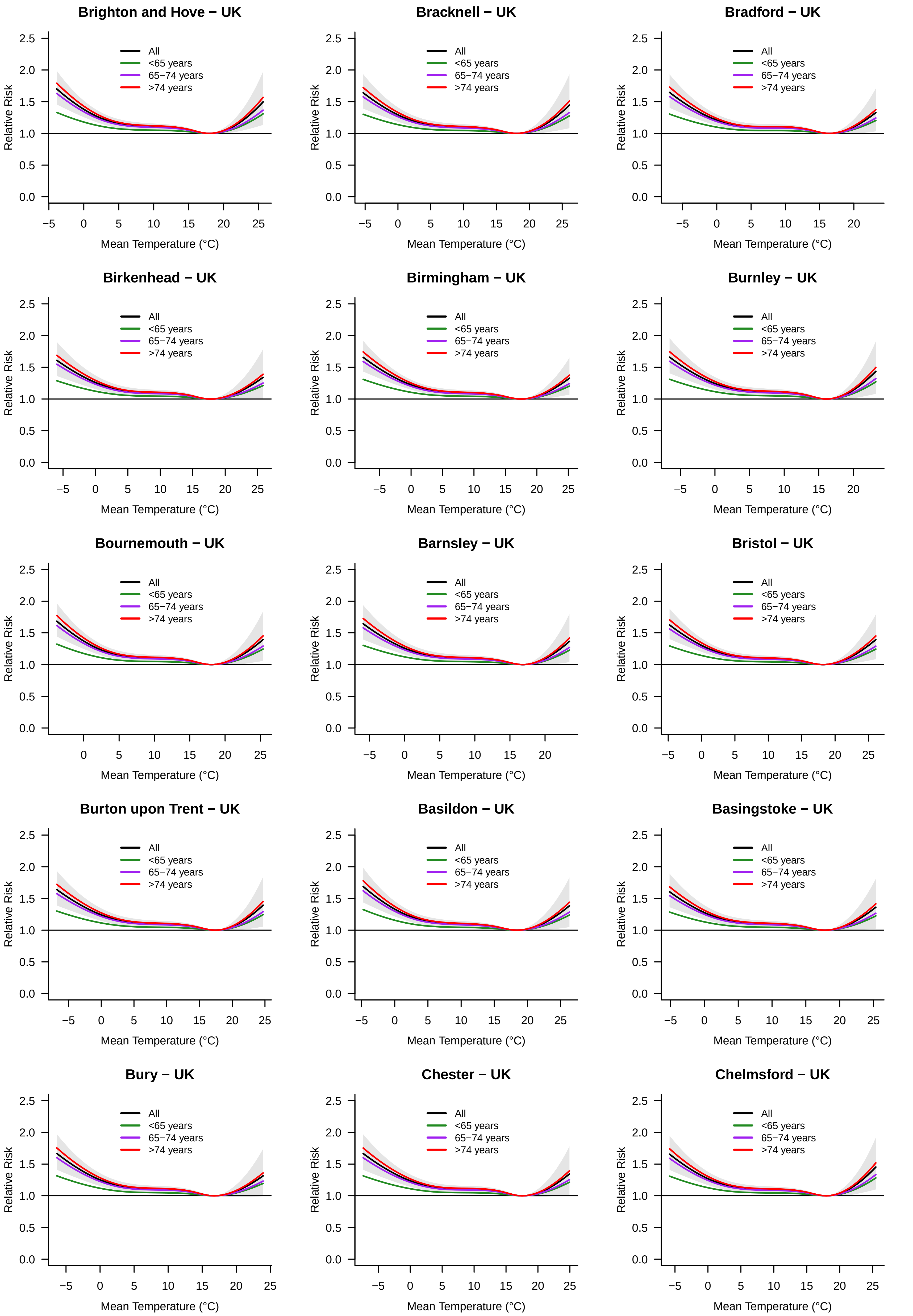
Brighton and Hove − UK
Bracknell − UK
Bradford − UK
Birkenhead − UK
Birmingham − UK
Burnley − UK
Bournemouth − UK
Barnsley − UK
Bristol − UK
Burton upon Trent − UK
Basildon − UK
Basingstoke − UK
Bury − UK
Chester − UK
Chelmsford − UK
All
<65 years
65−74 years
>74 years
Relative Risk
Mean Temperature (°C)

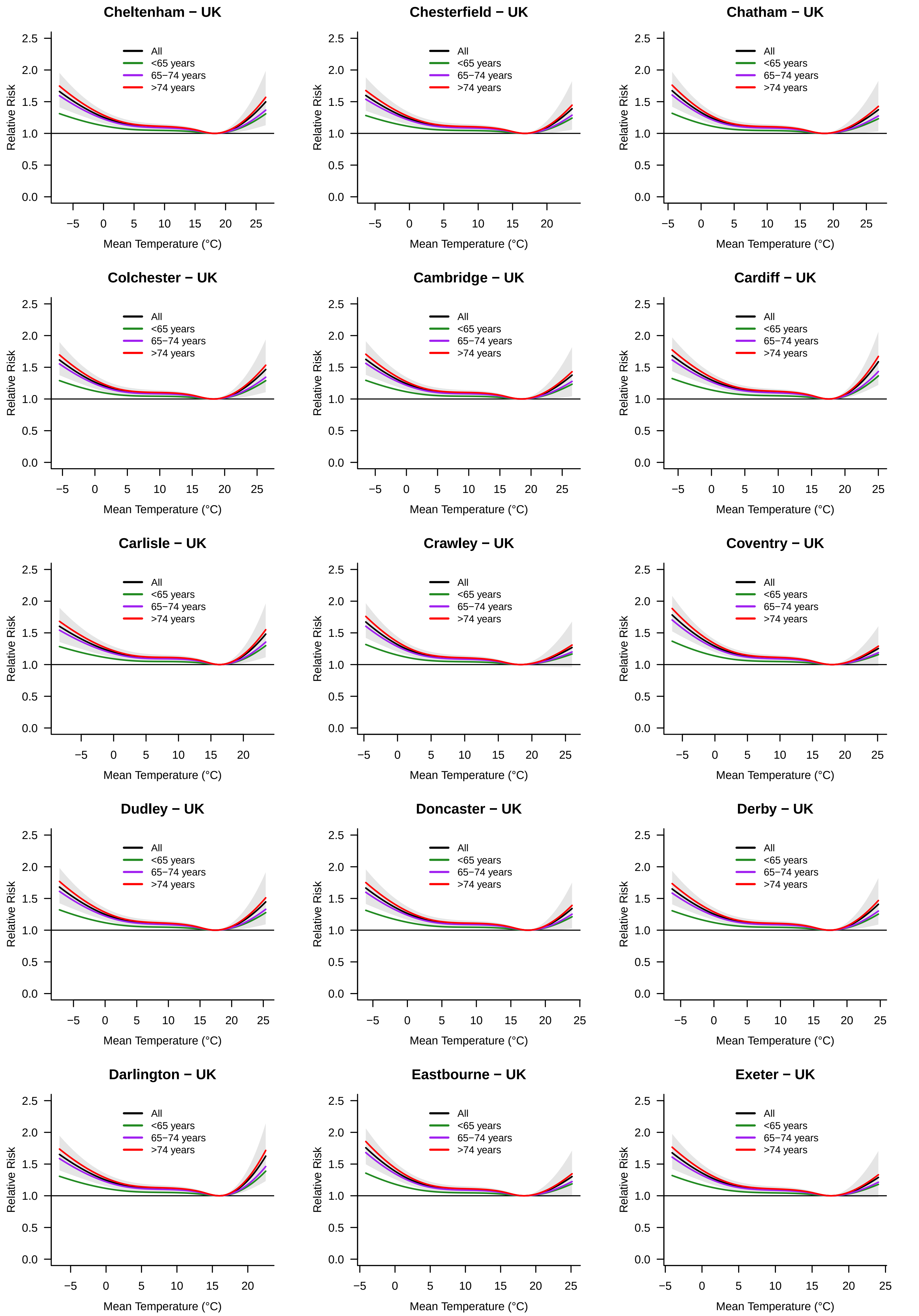

Cheltenham − UK
Chesterfield − UK
Chatham − UK
Colchester − UK
Cambridge − UK
Cardiff − UK
Carlisle − UK
Crawley − UK
Coventry − UK
Dudley − UK
Doncaster − UK
Derby − UK
Darlington − UK
Eastbourne − UK
Exeter − UK
All
<65 years
65−74 years
>74 years
Relative Risk
Mean Temperature (°C)

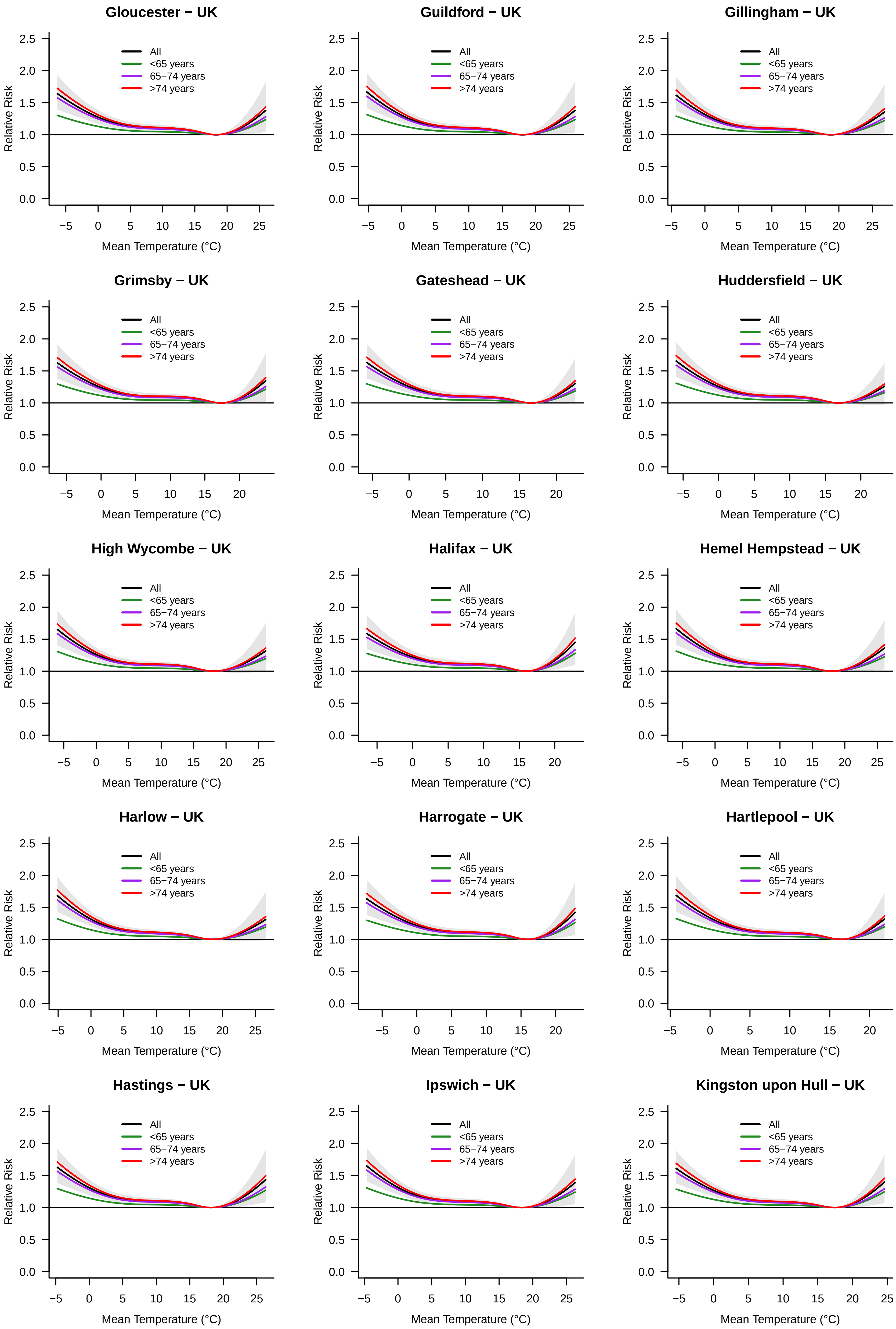
Gloucester − UK
Guildford − UK
Gillingham − UK
Grimsby − UK
Gateshead − UK
Huddersfield − UK
High Wycombe − UK
Halifax − UK
Hemel Hempstead − UK
Harlow − UK
Harrogate − UK
Hartlepool − UK
Hastings − UK
Ipswich − UK
Kingston upon Hull − UK
All
<65 years
65−74 years
>74 years
Relative Risk
Mean Temperature (°C)

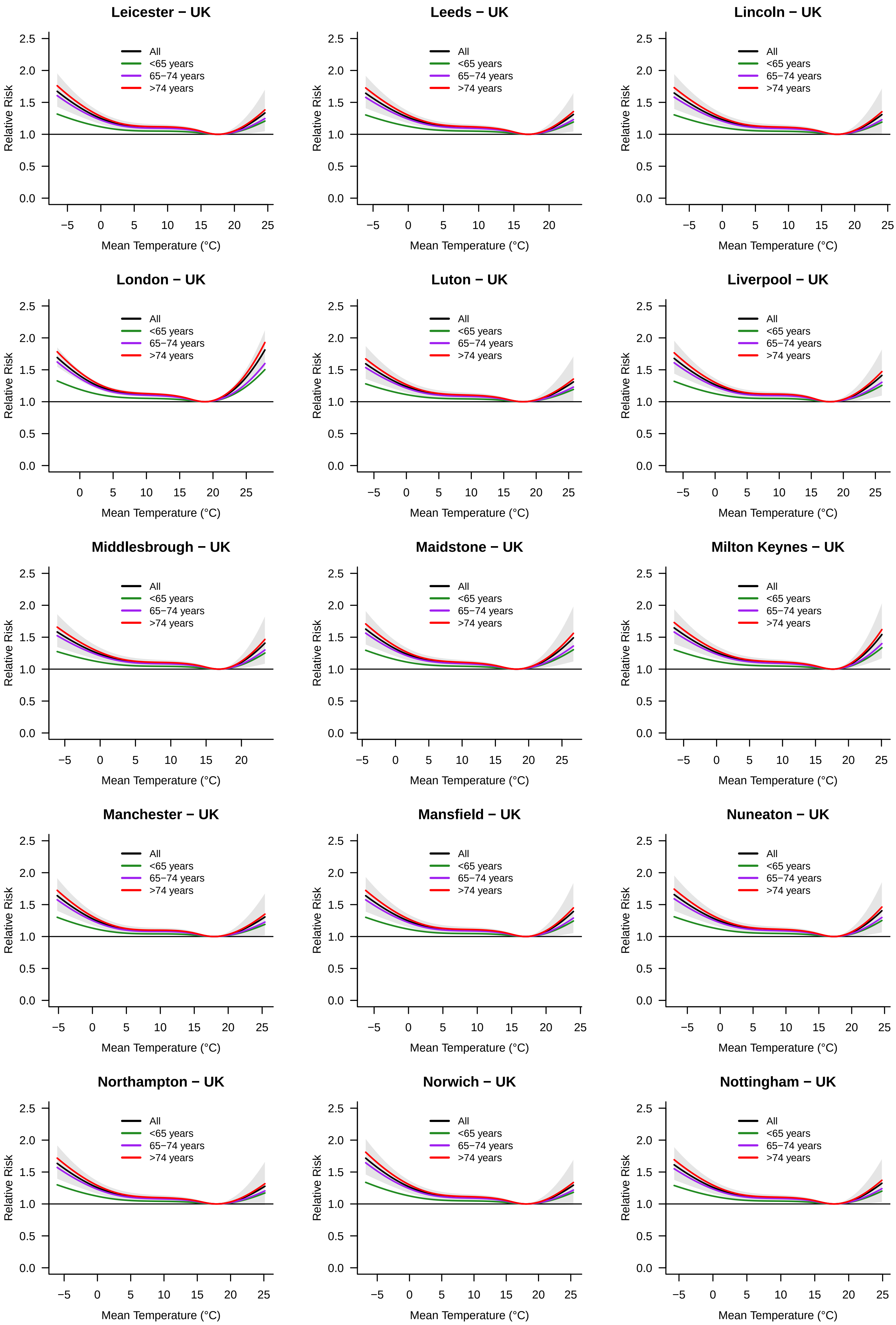
Leicester − UK
Leeds − UK
Lincoln − UK
London − UK
Luton − UK
Liverpool − UK
Middlesbrough − UK
Maidstone − UK
Milton Keynes − UK
Manchester − UK
Mansfield − UK
Nuneaton − UK
Northampton − UK
Norwich − UK
Nottingham − UK
All
<65 years
65−74 years
>74 years
Relative Risk
Mean Temperature (°C)

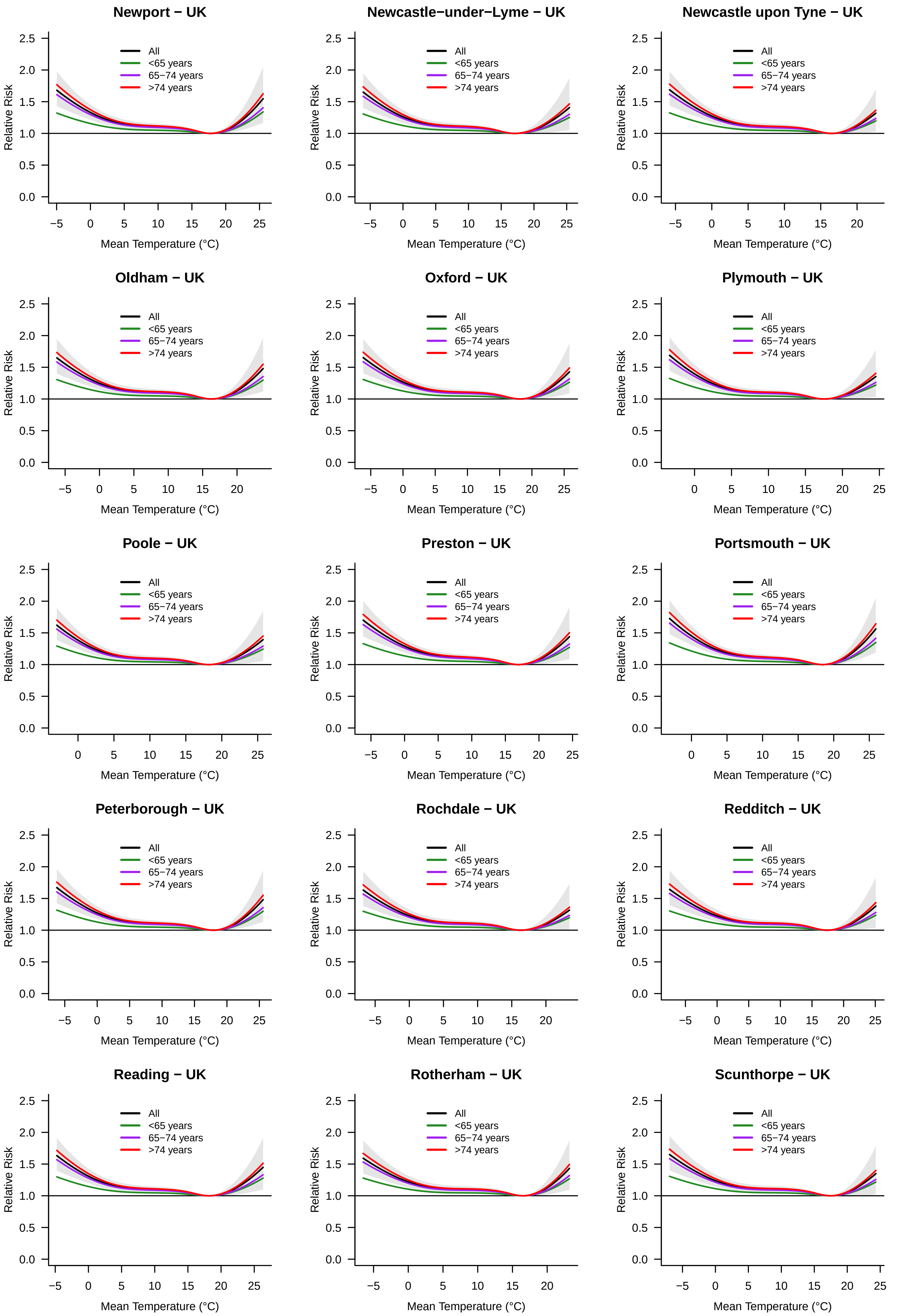
Newport − UK
Newcastle−under−Lyme − UK
Newcastle upon Tyne − UK
Oldham − UK
Oxford − UK
Plymouth − UK
Poole − UK
Preston − UK
Portsmouth − UK
Peterborough − UK
Rochdale − UK
Redditch − UK
Reading − UK
Rotherham − UK
Scunthorpe − UK
All
<65 years
65−74 years
>74 years
Relative Risk
Mean Temperature (°C)

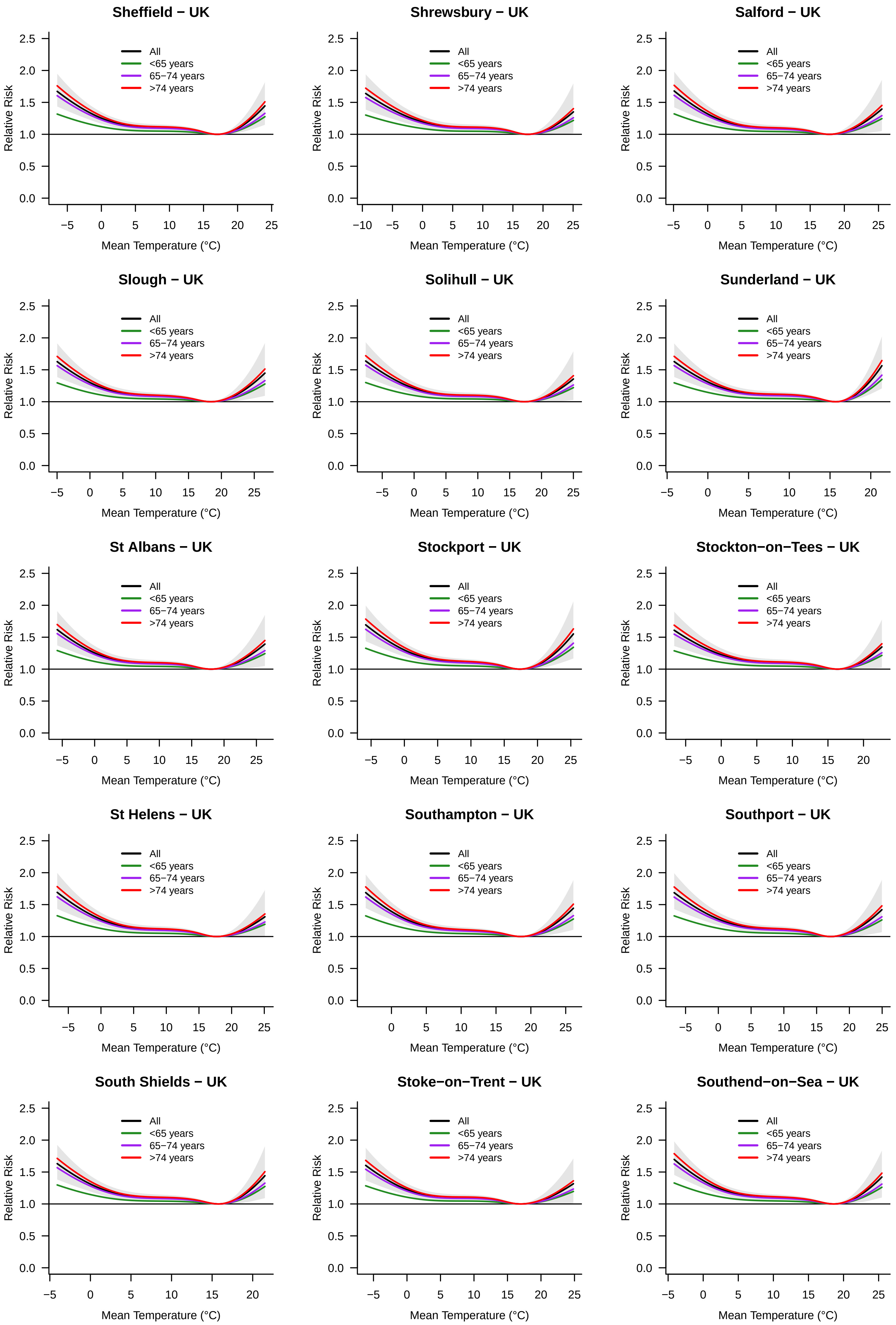
Sheffield − UK
Shrewsbury − UK
Salford − UK
Slough − UK
Solihull − UK
Sunderland − UK
St Albans − UK
Stockport − UK
Stockton−on−Tees − UK
St Helens − UK
Southampton − UK
Southport − UK
South Shields − UK
Stoke−on−Trent − UK
Southend−on−Sea − UK
All
<65 years
65−74 years
>74 years
Relative Risk
Mean Temperature (°C)

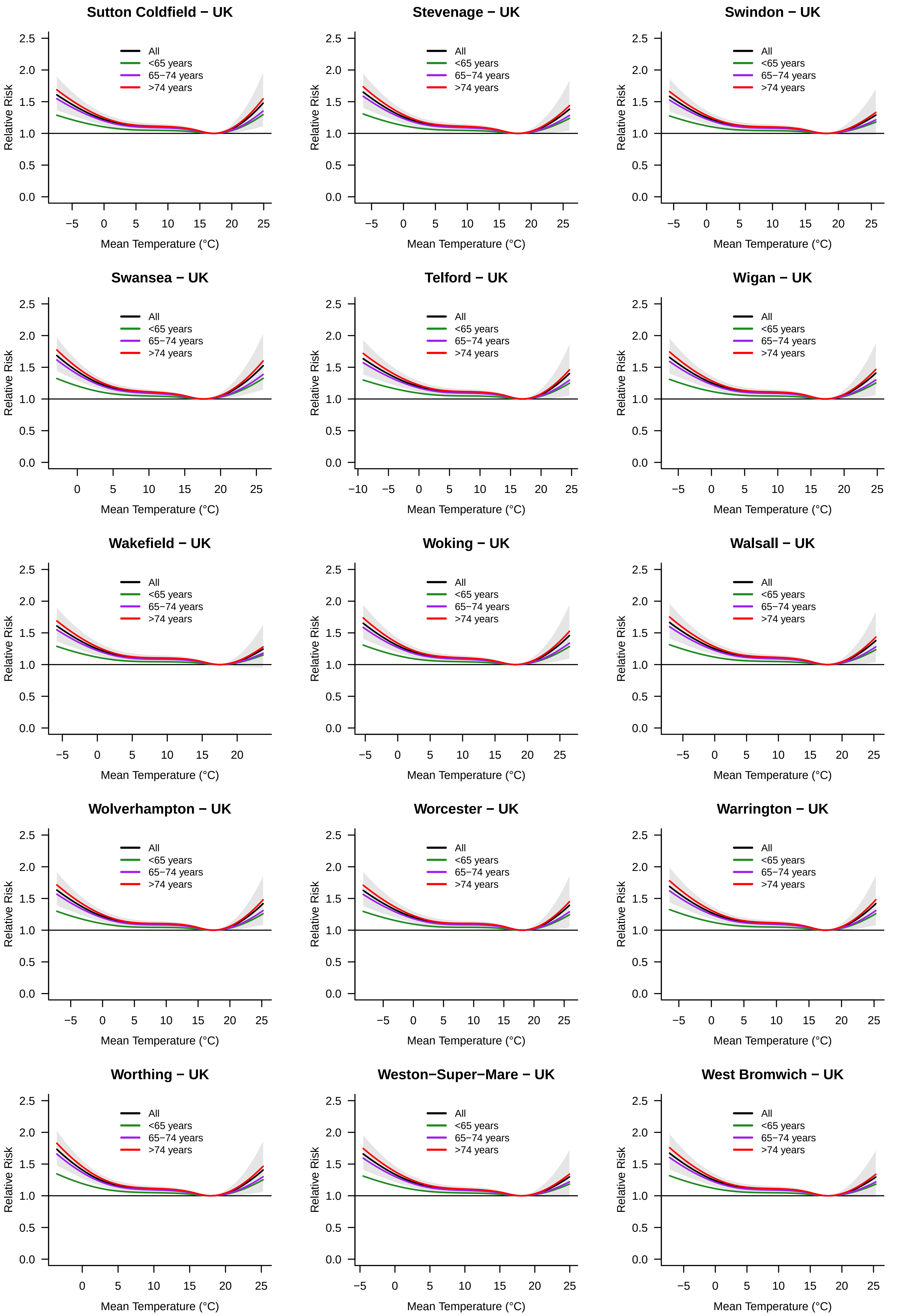

Sutton Coldfield − UK
Stevenage − UK
Swindon − UK
Swansea − UK
Telford − UK
Wigan − UK
Wakefield − UK
Woking − UK
Walsall − UK
Wolverhampton − UK
Worcester − UK
Warrington − UK
Worthing − UK
Weston−Super−Mare − UK
West Bromwich − UK
All
<65 years
65−74 years
>74 years
Relative Risk
Mean Temperature (°C)

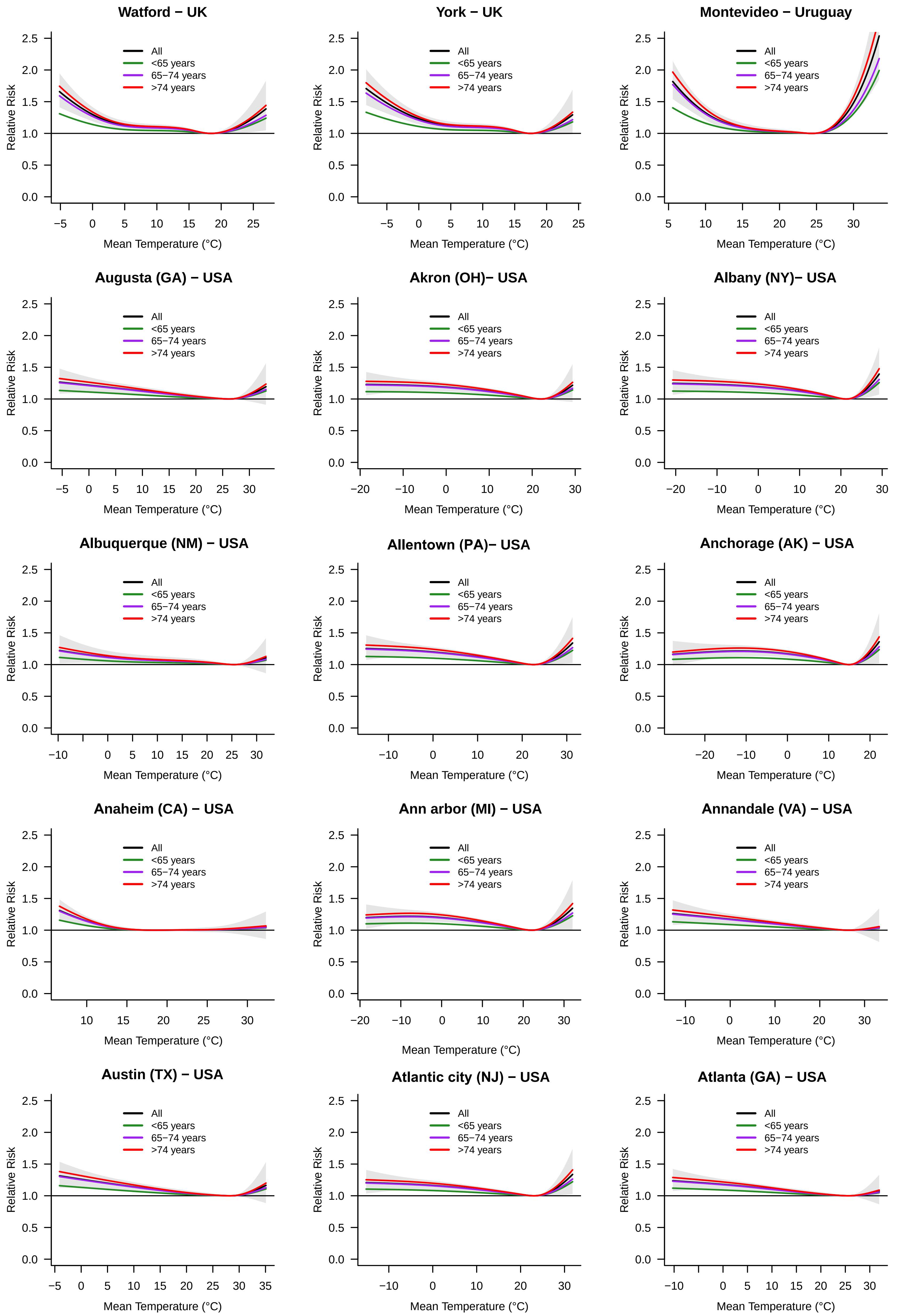
Watford − UK
York − UK
Montevideo − Uruguay
Augusta (GA) − USA
Akron (OH)− USA
Albany (NY)− USA
Albuquerque (NM) − USA
Allentown (PA)− USA
Anchorage (AK) − USA
Anaheim (CA) − USA
Ann arbor (MI) − USA
Annandale (VA) − USA
Austin (TX) − USA
Atlantic city (NJ) − USA
Atlanta (GA) − USA
All
<65 years
65−74 years
>74 years
Relative Risk
Mean Temperature (°C)

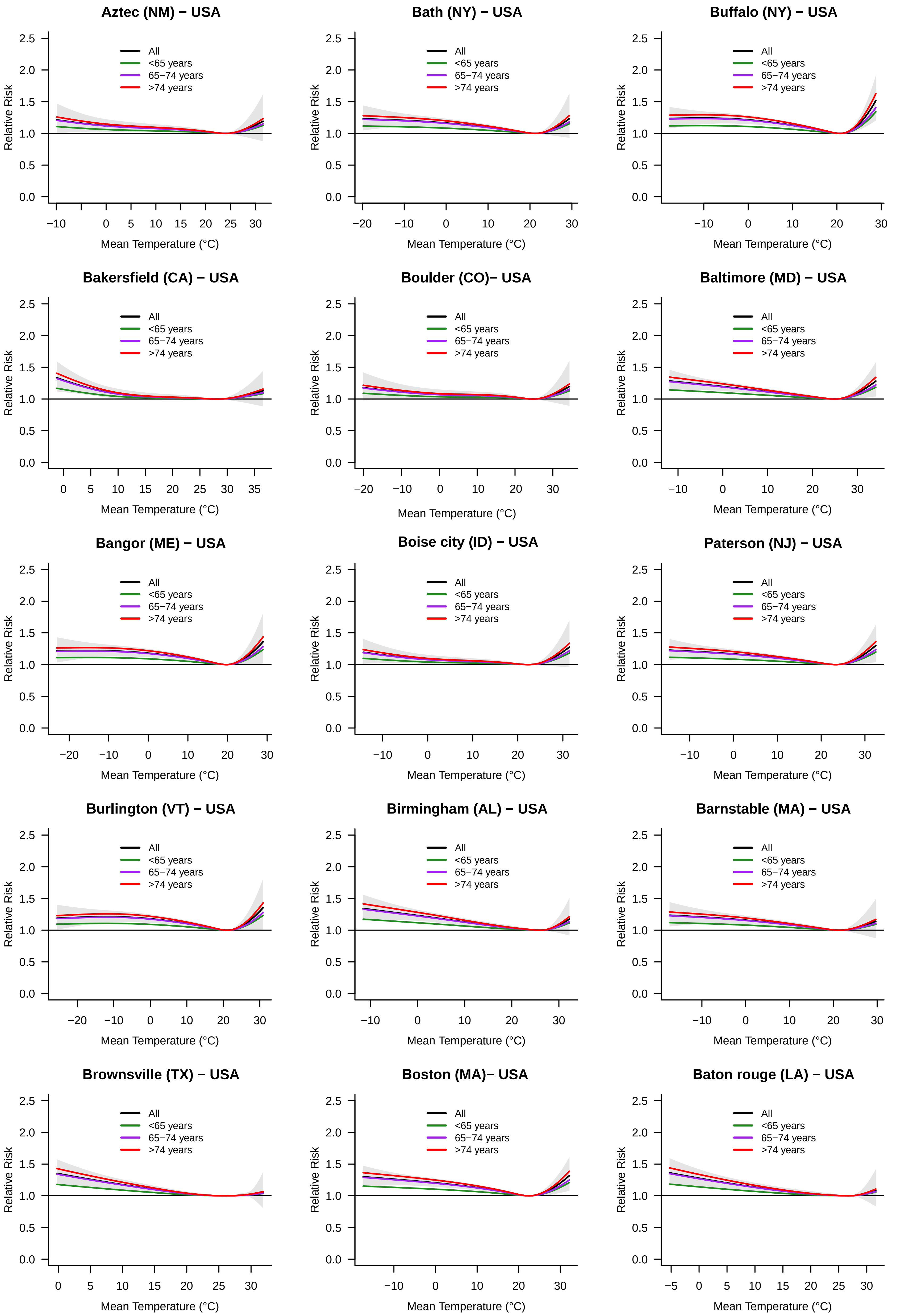

Aztec (NM) − USA
Bath (NY) − USA
Buffalo (NY) − USA
Bakersfield (CA) − USA
Boulder (CO)− USA
Baltimore (MD) − USA
Bangor (ME) − USA
Boise city (ID) − USA
Paterson (NJ) − USA
Burlington (VT) − USA
Birmingham (AL) − USA
Barnstable (MA) − USA
Brownsville (TX) − USA
Boston (MA)− USA
Baton rouge (LA) − USA
All
<65 years
65−74 years
>74 years
Relative Risk
Mean Temperature (°C)

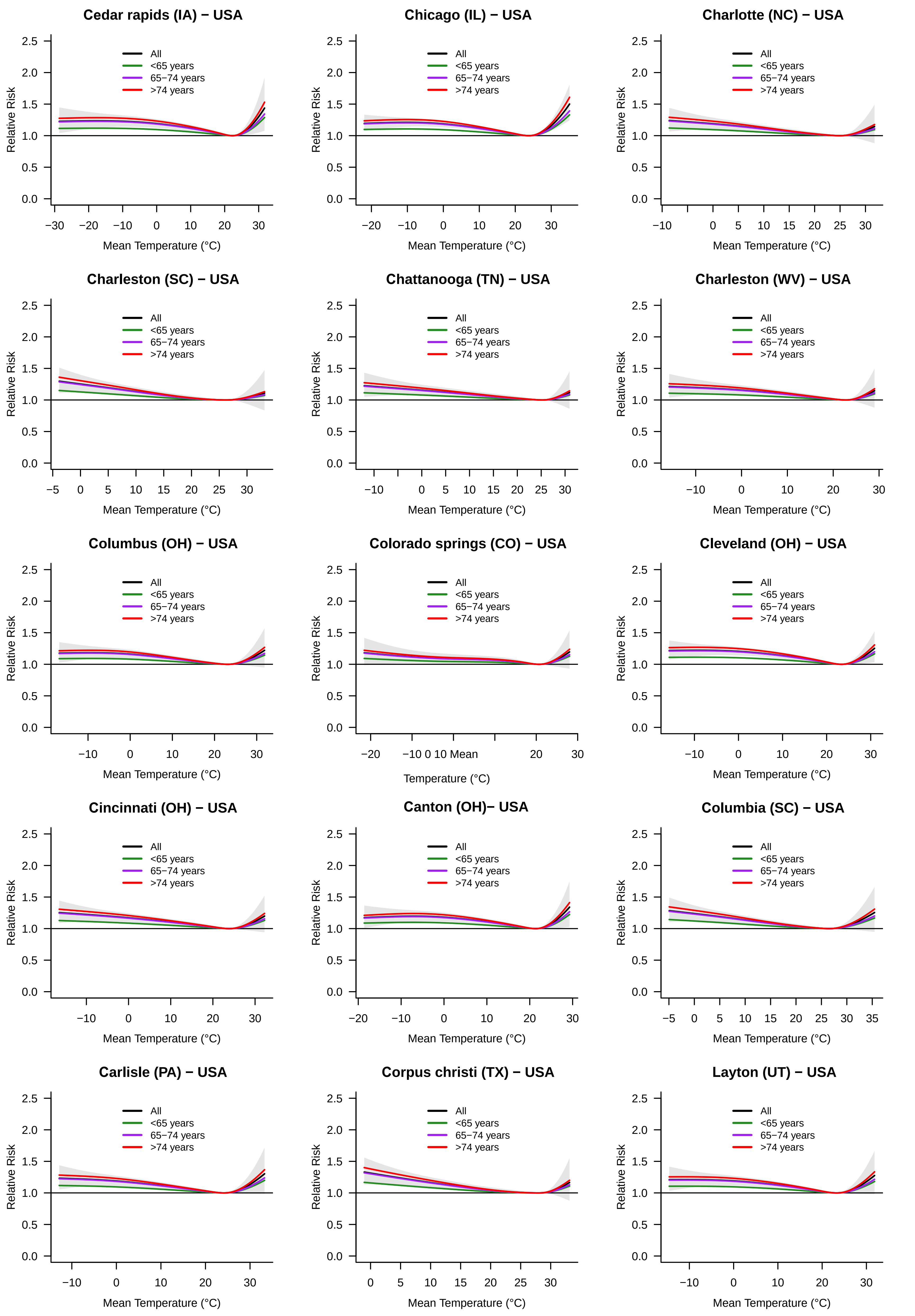

Cedar rapids (IA) − USA
Chicago (IL) − USA
Charlotte (NC) − USA
Charleston (SC) − USA
Chattanooga (TN) − USA
Charleston (WV) − USA
Columbus (OH) − USA
Colorado springs (CO) − USA
Cleveland (OH) − USA
Cincinnati (OH) − USA
Canton (OH)− USA
Columbia (SC) − USA
Carlisle (PA) − USA
Corpus christi (TX) − USA
Layton (UT) − USA
All
<65 years
65−74 years
>74 years
Relative Risk
Mean Temperature (°C)
−10 0 10 Mean
Temperature (°C)

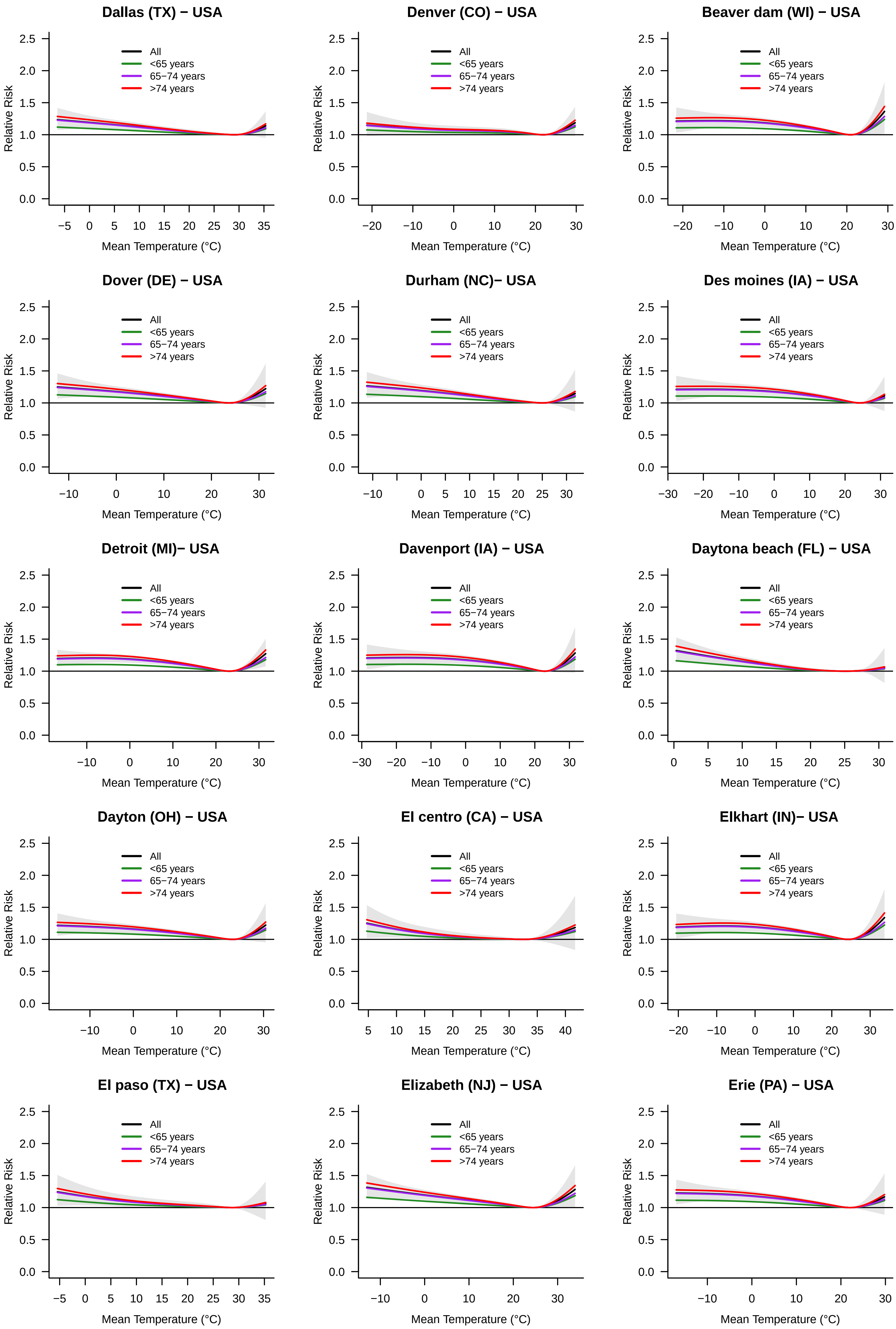
Dallas (TX) − USA
Denver (CO) − USA
Beaver dam (WI) − USA
Dover (DE) − USA
Durham (NC)− USA
Des moines (IA) − USA
Detroit (MI)− USA
Davenport (IA) − USA
Daytona beach (FL) − USA
Dayton (OH) − USA
El centro (CA) − USA
Elkhart (IN)− USA
El paso (TX) − USA
Elizabeth (NJ) − USA
Erie (PA) − USA
All
<65 years
65−74 years
>74 years
Relative Risk
0.0
0.5
1.0
1.5
2.0
2.5
Mean Temperature (°C)

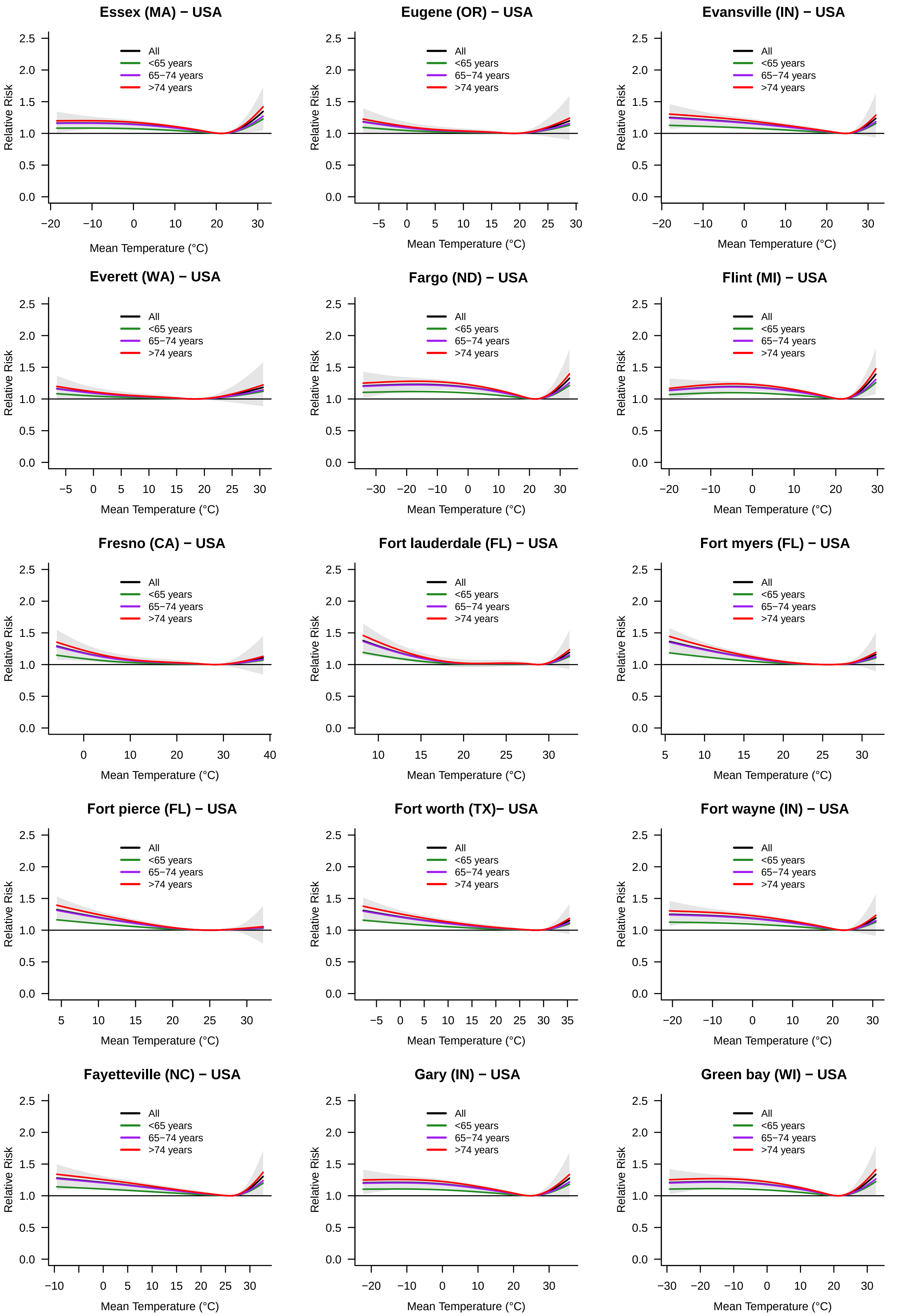
Essex (MA) − USA
Eugene (OR) − USA
Evansville (IN) − USA
Everett (WA) − USA
Fargo (ND) − USA
Flint (MI) − USA
Fresno (CA) − USA
Fort lauderdale (FL) − USA
Fort myers (FL) − USA
Fort pierce (FL) − USA
Fort worth (TX)− USA
Fort wayne (IN) − USA
Fayetteville (NC) − USA
Gary (IN) − USA
Green bay (WI) − USA
All
<65 years
65−74 years
>74 years
Relative Risk
0.0
0.5
1.0
1.5
2.0
2.5
Mean Temperature (°C)

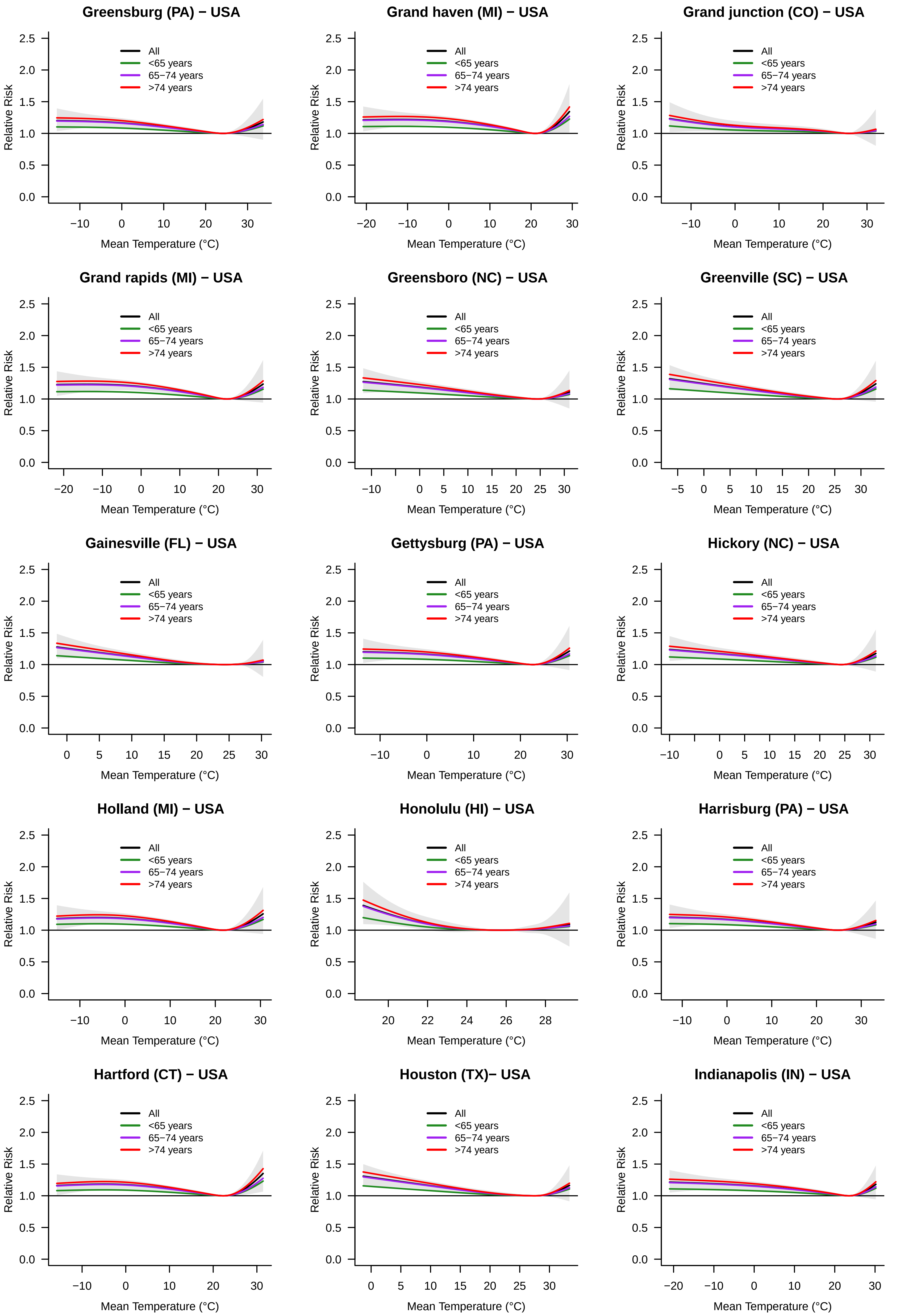
Greensburg (PA) − USA
Grand haven (MI) − USA
Grand junction (CO) − USA
Grand rapids (MI) − USA
Greensboro (NC) − USA
Greenville (SC) − USA
Gainesville (FL) − USA
Gettysburg (PA) − USA
Hickory (NC) − USA
Holland (MI) − USA
Honolulu (HI) − USA
Harrisburg (PA) − USA
Hartford (CT) − USA
Houston (TX)− USA
Indianapolis (IN) − USA
All
<65 years
65−74 years
>74 years
Relative Risk
Mean Temperature (°C)

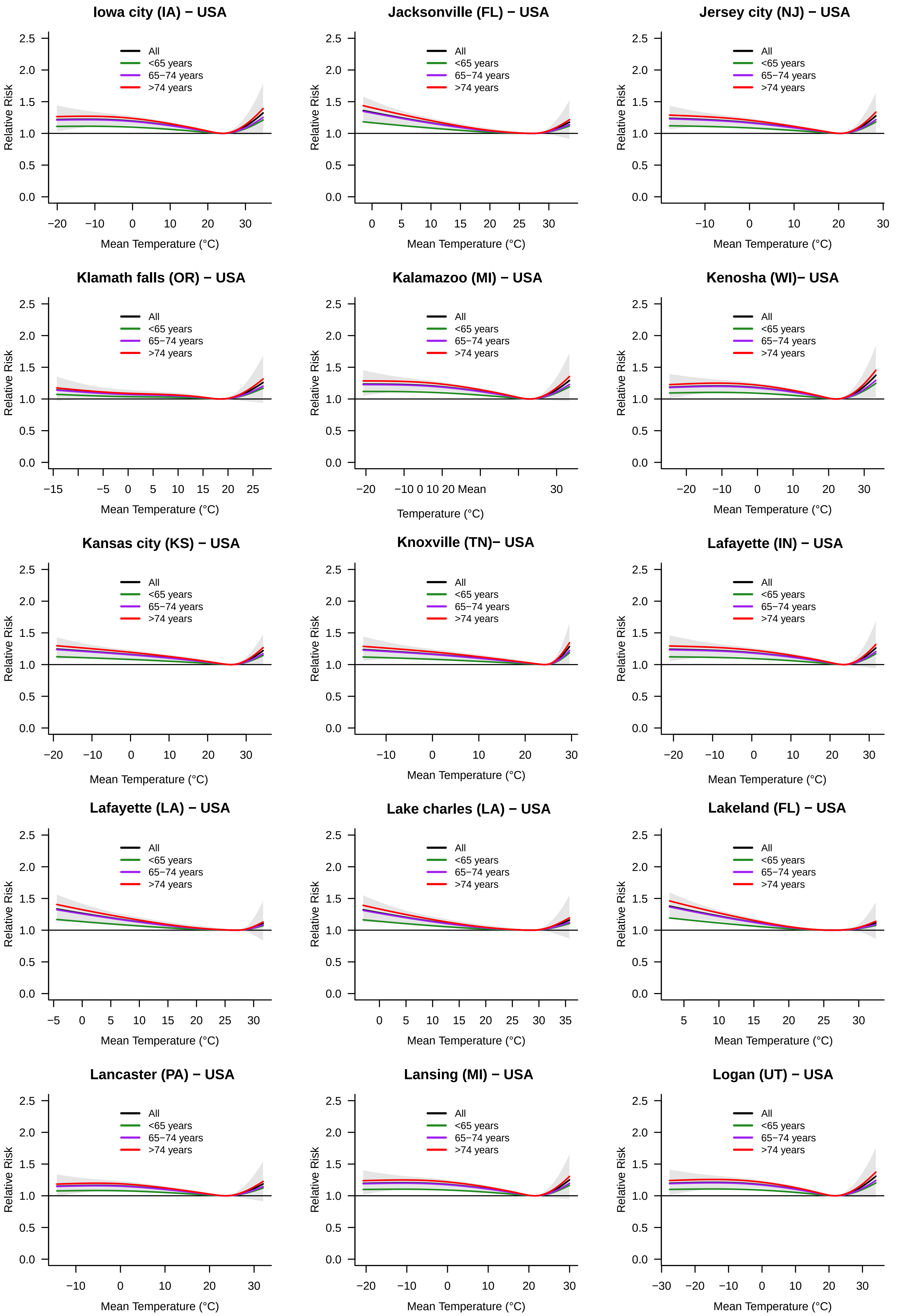

Iowa city (IA) − USA
Jacksonville (FL) − USA
Jersey city (NJ) − USA
Klamath falls (OR) − USA
Kalamazoo (MI) − USA
Kenosha (WI)− USA
Kansas city (KS) − USA
Knoxville (TN)− USA
Lafayette (IN) − USA
Lafayette (LA) − USA
Lake charles (LA) − USA
Lakeland (FL) − USA
Lancaster (PA) − USA
Lansing (MI) − USA
Logan (UT) − USA
All
<65 years
65−74 years
>74 years
Relative Risk
0.0
0.5
1.0
1.5
2.0
2.5
Mean Temperature (°C)
−10 0 10 20 Mean
Temperature (°C)

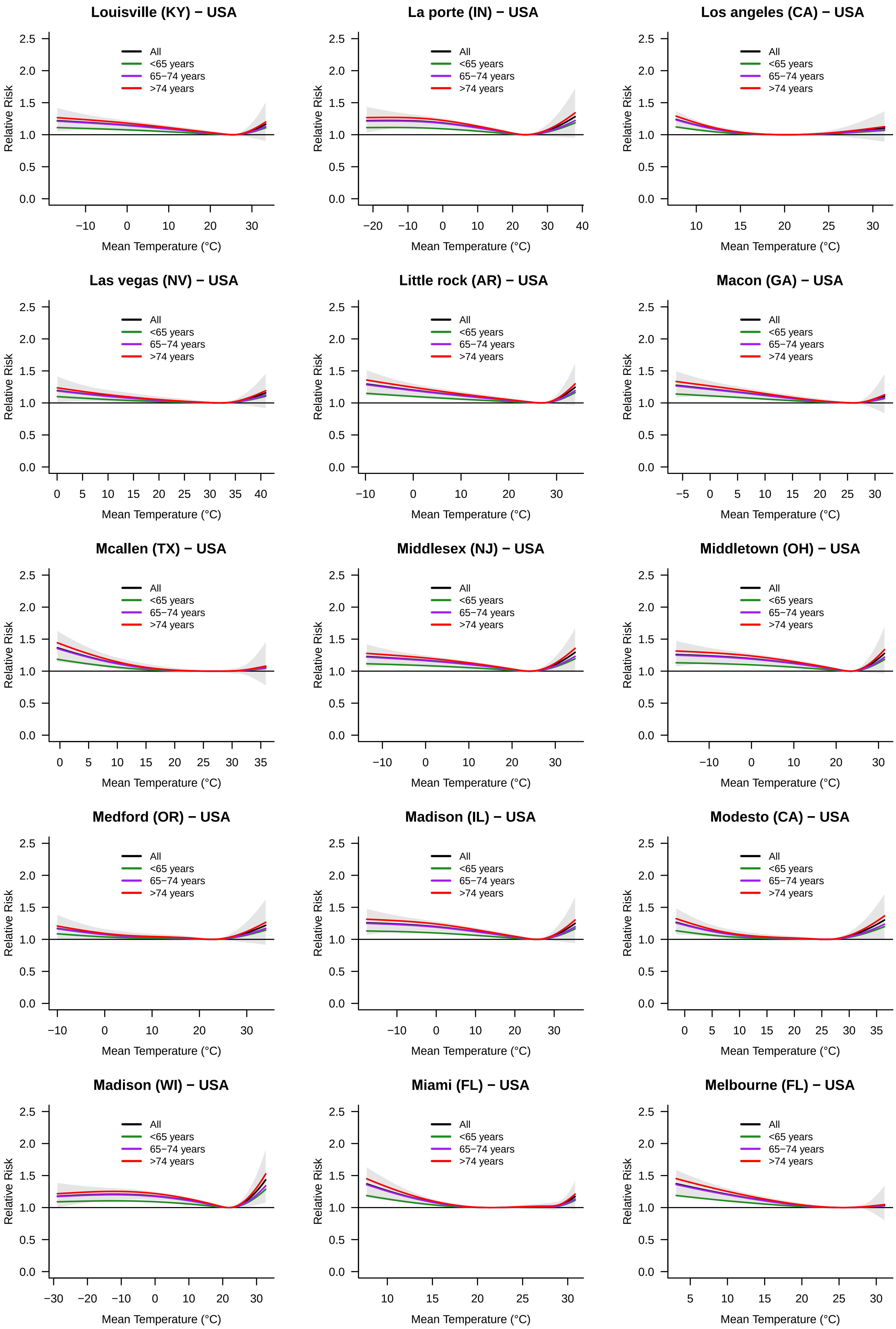
Louisville (KY) − USA
La porte (IN) − USA
Los angeles (CA) − USA
Las vegas (NV) − USA
Little rock (AR) − USA
Macon (GA) − USA
Mcallen (TX) − USA
Middlesex (NJ) − USA
Middletown (OH) − USA
Medford (OR) − USA
Madison (IL) − USA
Modesto (CA) − USA
Madison (WI) − USA
Miami (FL) − USA
Melbourne (FL) − USA
All
<65 years
65−74 years
>74 years
Relative Risk
0.0
0.5
1.0
1.5
2.0
2.5
Mean Temperature (°C)
−10 0 10 20 30
−20 −10 0 10 20 30 40
10 15 20 25 30
0 5 10 15 20 25 30 35 40
−10 0 10 20 30
−5 0 5 10 15 20 25 30
0 5 10 15 20 25 30 35
−10 0 10 20 30
−10 0 10 20 30
−10 0 10 20 30
−10 0 10 20 30
0 5 10 15 20 25 30 35
−30 −20 −10 0 10 20 30
10 15 20 25 30
5 10 15 20 25 30

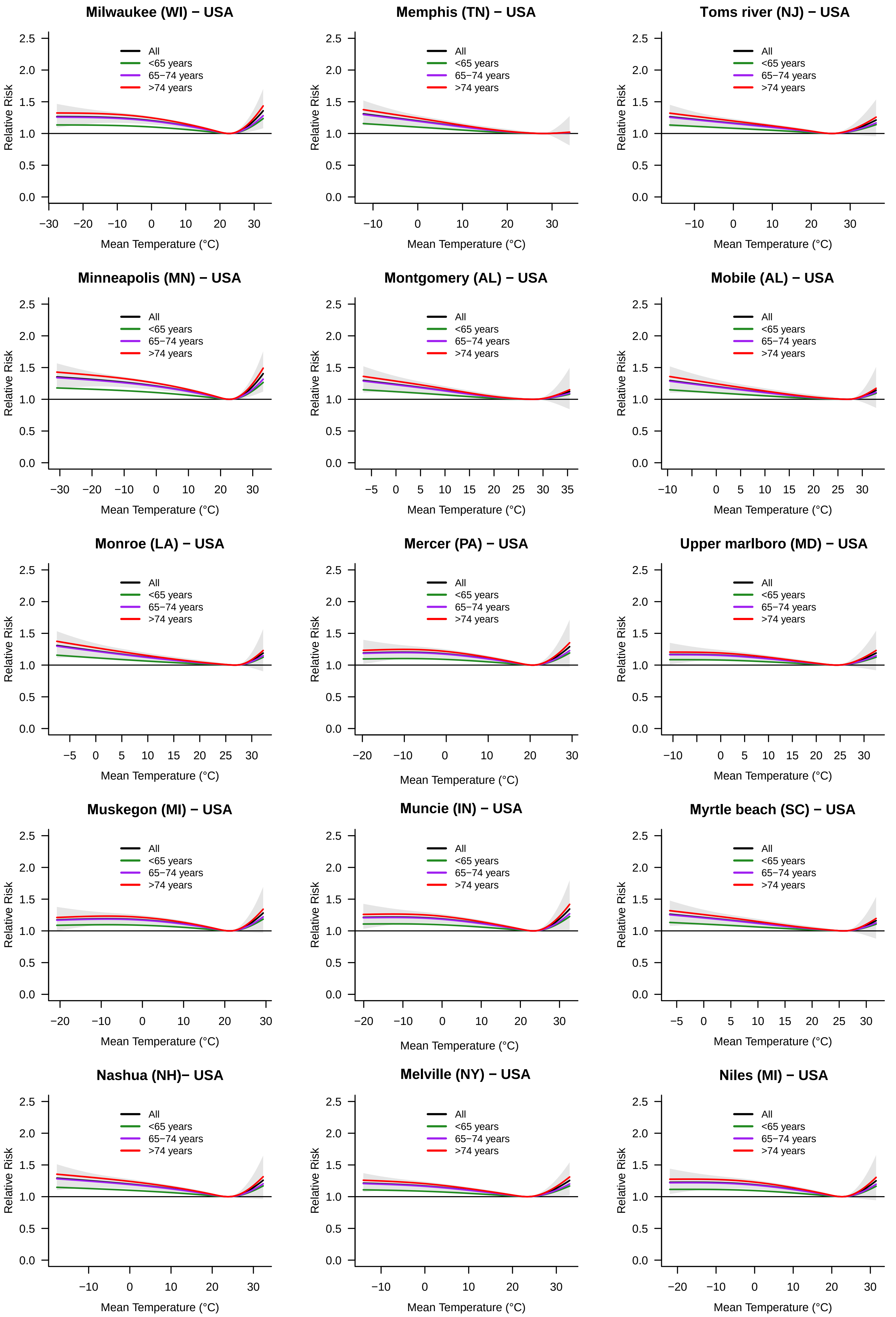
Milwaukee (WI) − USA
Memphis (TN) − USA
Toms river (NJ) − USA
Minneapolis (MN) − USA
Montgomery (AL) − USA
Mobile (AL) − USA
Monroe (LA) − USA
Mercer (PA) − USA
Upper marlboro (MD) − USA
Muskegon (MI) − USA
Muncie (IN) − USA
Myrtle beach (SC) − USA
Nashua (NH)− USA
Melville (NY) − USA
Niles (MI) − USA
All
<65 years
65−74 years
>74 years
Relative Risk
0.0
0.5
1.0
1.5
2.0
2.5
Mean Temperature (°C)

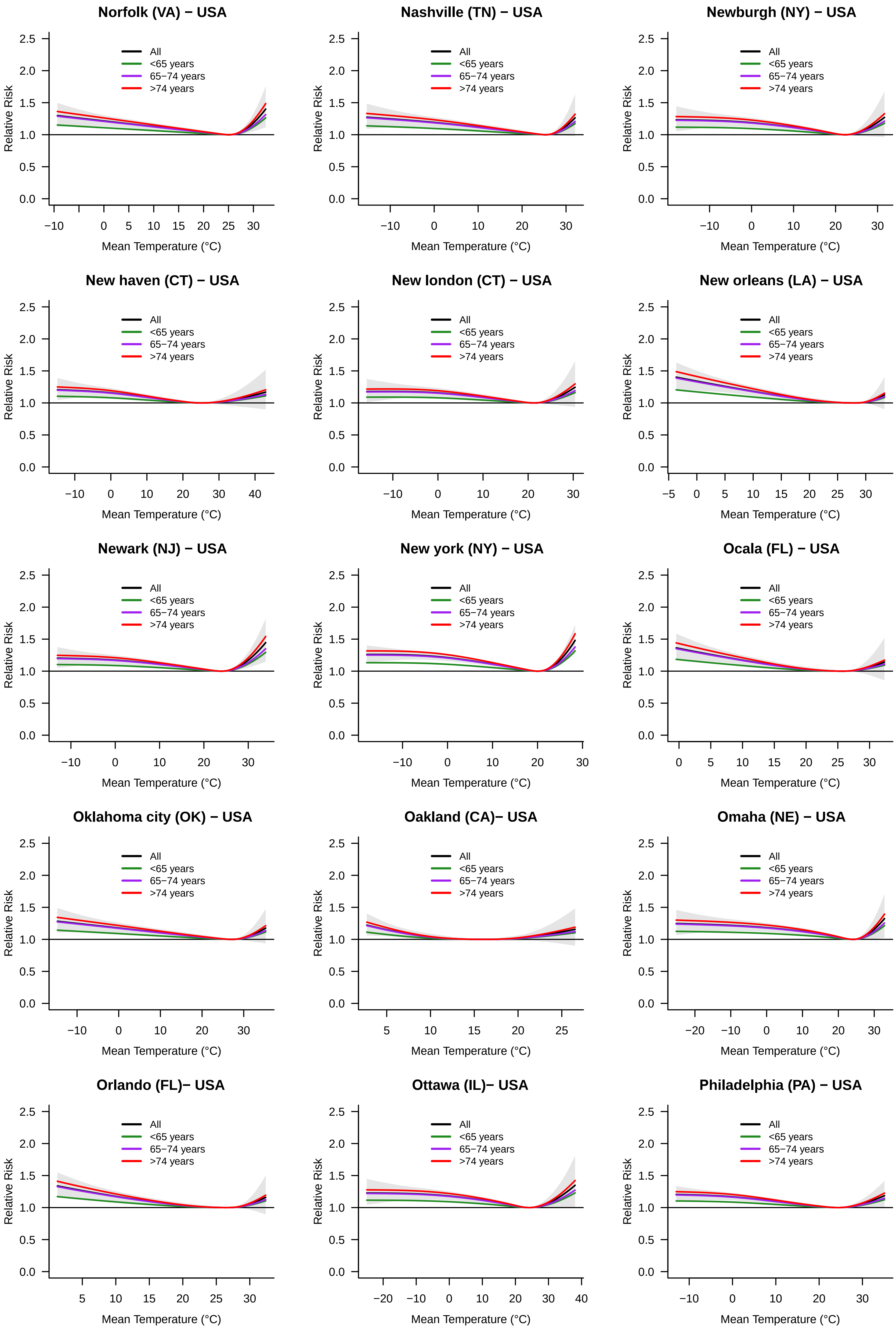
Norfolk (VA) − USA
Nashville (TN) − USA
Newburgh (NY) − USA
New haven (CT) − USA
New london (CT) − USA
New orleans (LA) − USA
Newark (NJ) − USA
New york (NY) − USA
Ocala (FL) − USA
Oklahoma city (OK) − USA
Oakland (CA)− USA
Omaha (NE) − USA
Orlando (FL)− USA
Ottawa (IL)− USA
Philadelphia (PA) − USA
All
<65 years
65−74 years
>74 years
Relative Risk
Mean Temperature (°C)

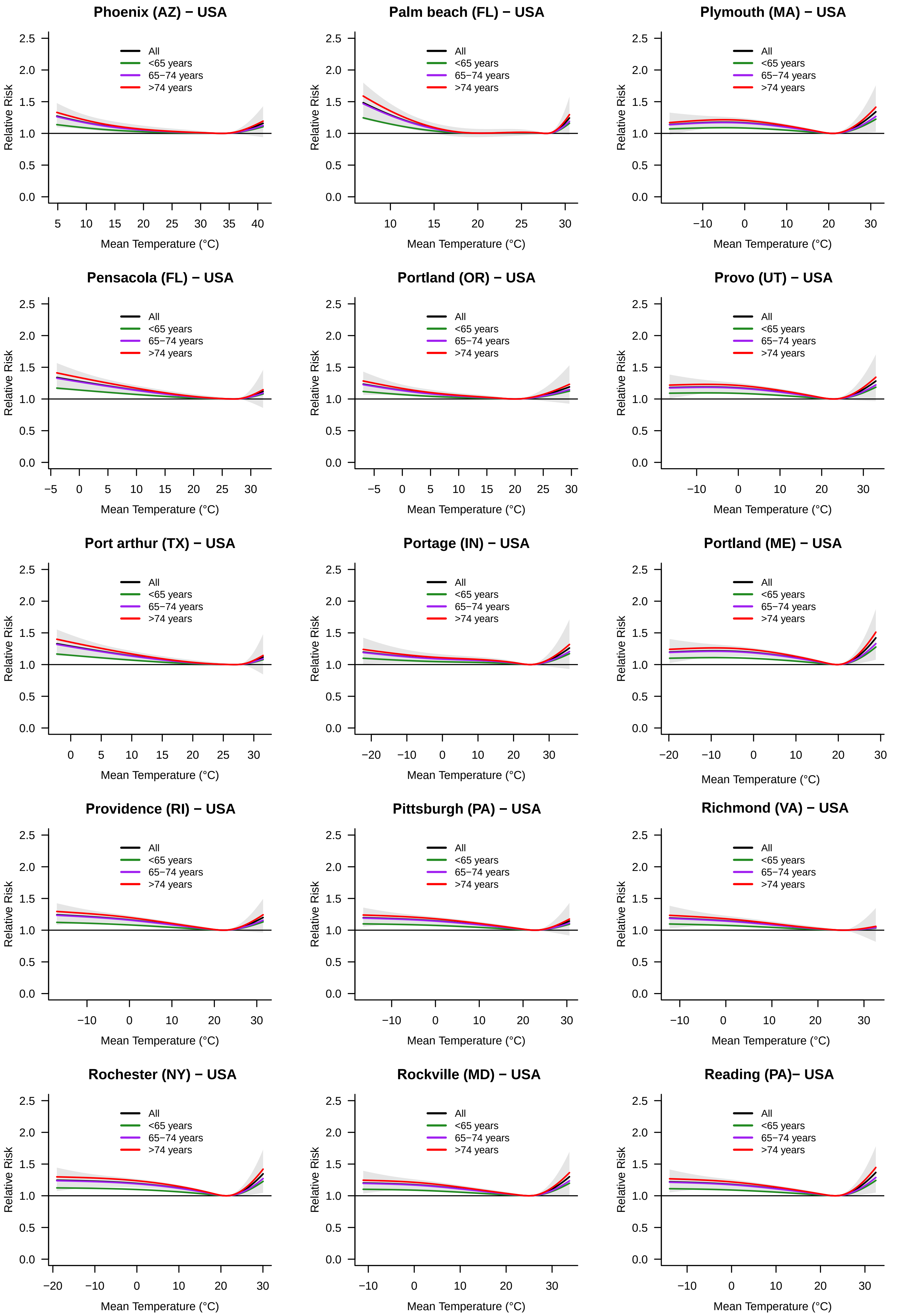
Phoenix (AZ) − USA
Palm beach (FL) − USA
Plymouth (MA) − USA
Pensacola (FL) − USA
Portland (OR) − USA
Provo (UT) − USA
Port arthur (TX) − USA
Portage (IN) − USA
Portland (ME) − USA
Providence (RI) − USA
Pittsburgh (PA) − USA
Richmond (VA) − USA
Rochester (NY) − USA
Rockville (MD) − USA
Reading (PA)− USA
All
<65 years
65−74 years
>74 years
Relative Risk
Mean Temperature (°C)

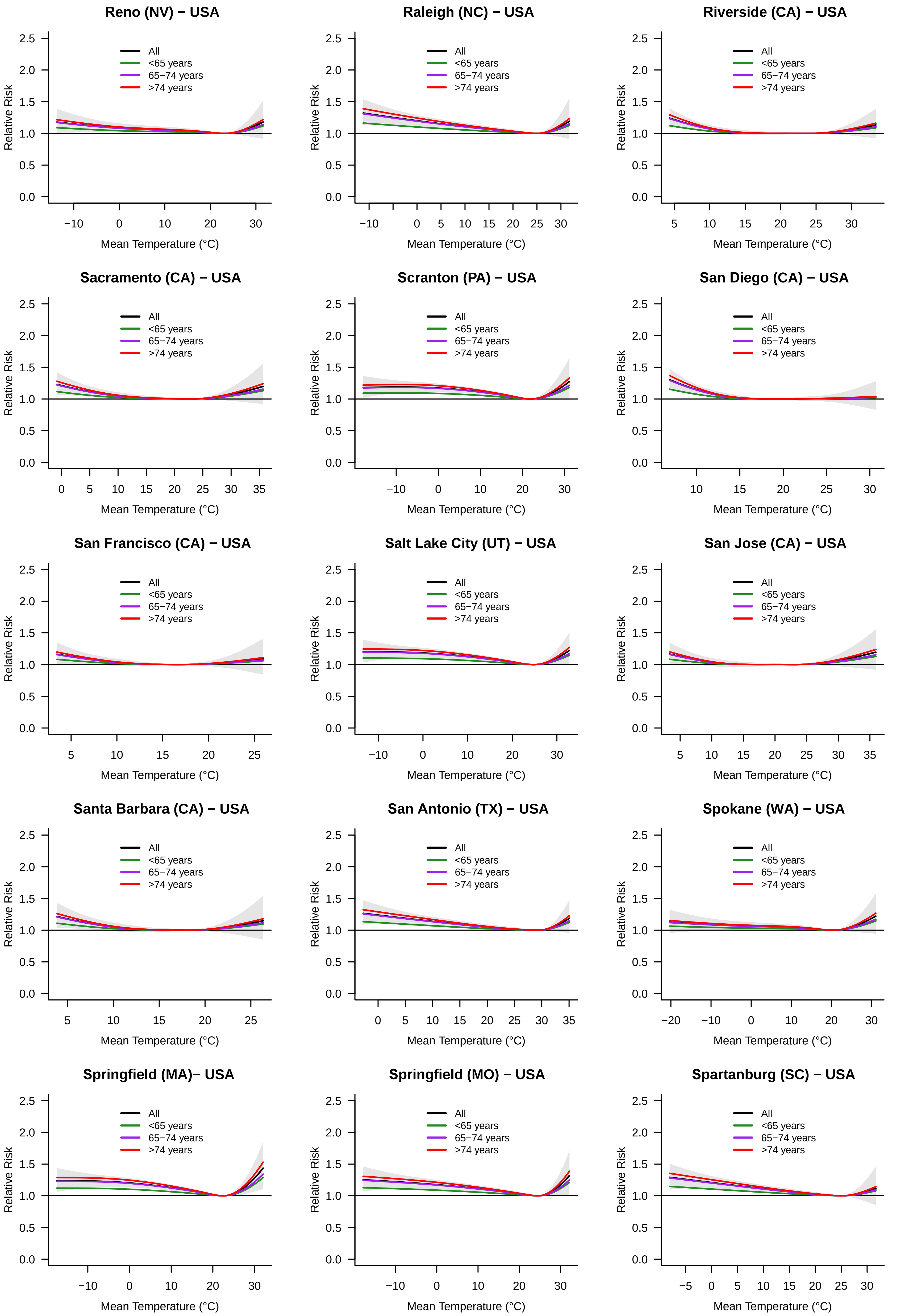
Reno (NV) − USA
Raleigh (NC) − USA
Riverside (CA) − USA
Sacramento (CA) − USA
Scranton (PA) − USA
San Diego (CA) − USA
San Francisco (CA) − USA
Salt Lake City (UT) − USA
San Jose (CA) − USA
Santa Barbara (CA) − USA
San Antonio (TX) − USA
Spokane (WA) − USA
Springfield (MA)− USA
Springfield (MO) − USA
Spartanburg (SC) − USA
All
<65 years
65−74 years
>74 years
Relative Risk
Mean Temperature (°C)

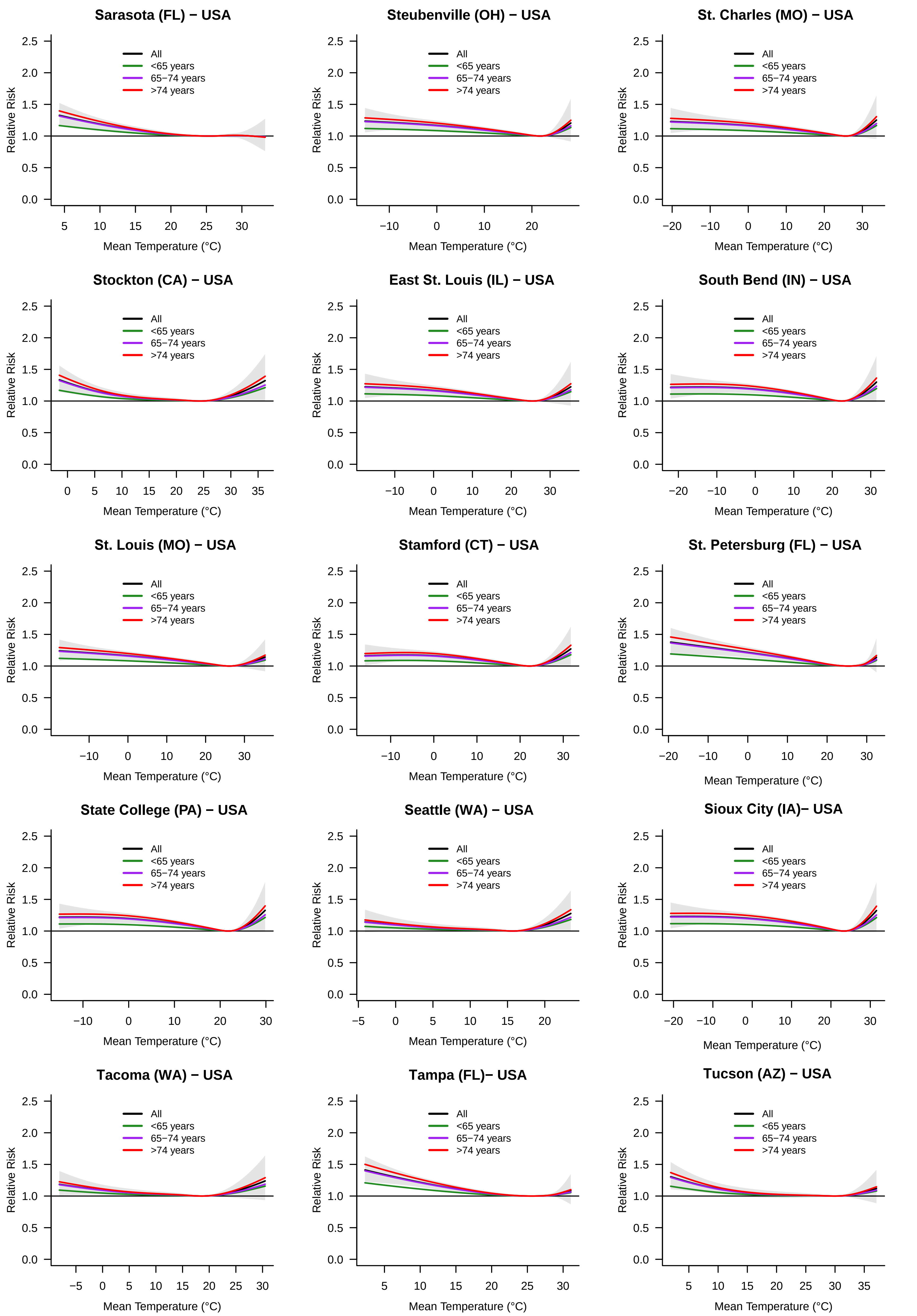
Sarasota (FL) − USA
Relative Risk
0.0 0.5 1.0 1.5 2.0 2.5
All
<65 years
65−74 years
>74 years
5 10 15 20 25 30
Mean Temperature (°C)
Steubenville (OH) − USA
Relative Risk
All
<65 years
65−74 years
>74 years
−10 0 10 20
Mean Temperature (°C)
St. Charles (MO) − USA
Relative Risk
All
<65 years
65−74 years
>74 years
−20 −10 0 10 20 30
Mean Temperature (°C)
Stockton (CA) − USA
Relative Risk
All
<65 years
65−74 years
>74 years
0 5 10 15 20 25 30 35
Mean Temperature (°C)
East St. Louis (IL) − USA
Relative Risk
All
<65 years
65−74 years
>74 years
−10 0 10 20 30
Mean Temperature (°C)
South Bend (IN) − USA
Relative Risk
All
<65 years
65−74 years
>74 years
−20 −10 0 10 20 30
Mean Temperature (°C)
St. Louis (MO) − USA
Relative Risk
All
<65 years
65−74 years
>74 years
−10 0 10 20 30
Mean Temperature (°C)
Stamford (CT) − USA
Relative Risk
All
<65 years
65−74 years
>74 years
−10 0 10 20 30
Mean Temperature (°C)
St. Petersburg (FL) − USA
Relative Risk
All
<65 years
65−74 years
>74 years
−20 −10 0 10 20 30
Mean Temperature (°C)
State College (PA) − USA
Relative Risk
All
<65 years
65−74 years
>74 years
−10 0 10 20 30
Mean Temperature (°C)
Seattle (WA) − USA
Relative Risk
All
<65 years
65−74 years
>74 years
−5 0 5 10 15 20
Mean Temperature (°C)
Sioux City (IA)− USA
Relative Risk
All
<65 years
65−74 years
>74 years
−20 −10 0 10 20 30
Mean Temperature (°C)
Tacoma (WA) − USA
Relative Risk
All
<65 years
65−74 years
>74 years
−5 0 5 10 15 20 25 30
Mean Temperature (°C)
Tampa (FL)− USA
Relative Risk
All
<65 years
65−74 years
>74 years
5 10 15 20 25 30
Mean Temperature (°C)
Tucson (AZ) − USA
Relative Risk
All
<65 years
65−74 years
>74 years
5 10 15 20 25 30 35
Mean Temperature (°C)

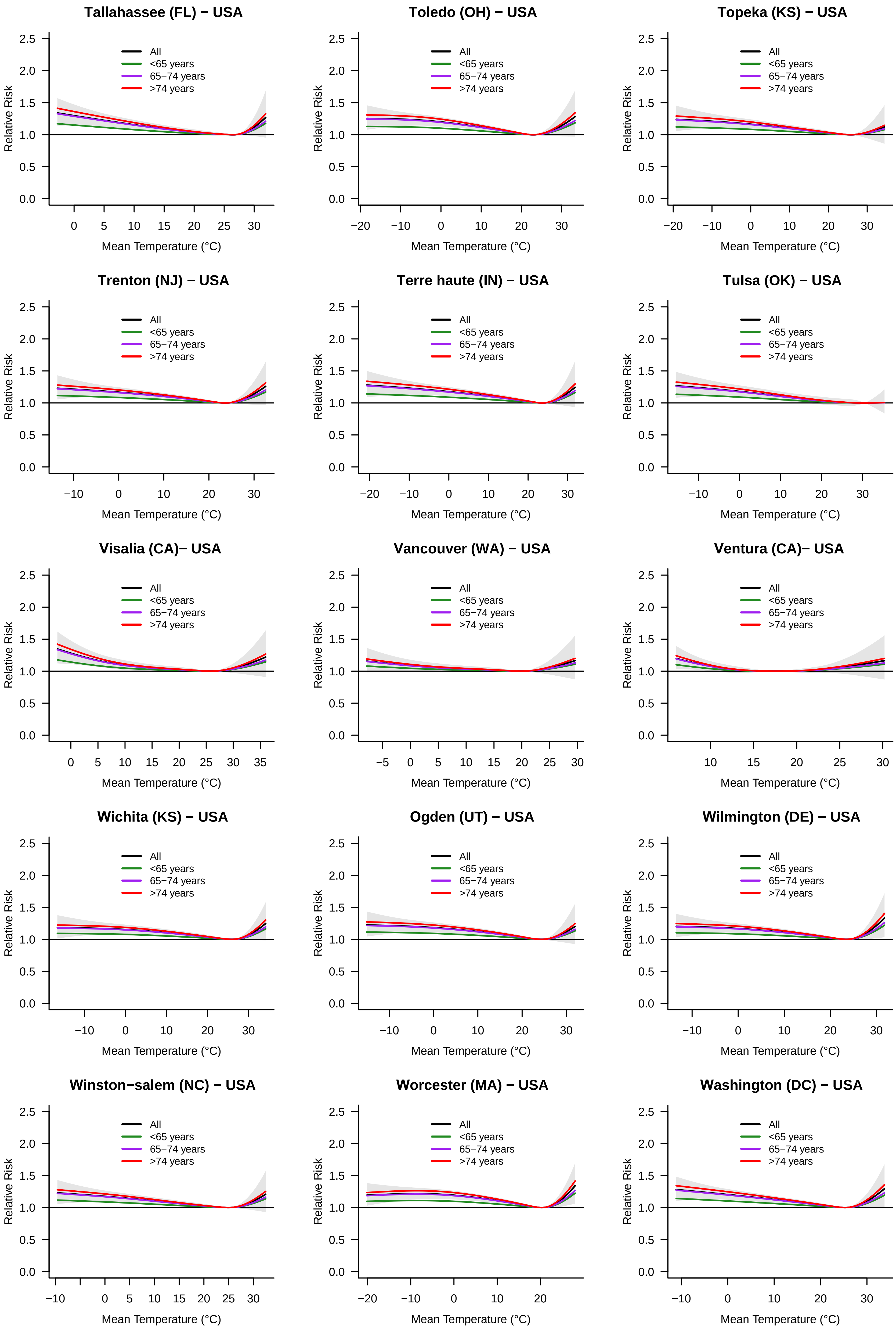

Tallahassee (FL) − USA
Toledo (OH) − USA
Topeka (KS) − USA
Trenton (NJ) − USA
Terre haute (IN) − USA
Tulsa (OK) − USA
Visalia (CA)− USA
Vancouver (WA) − USA
Ventura (CA)− USA
Wichita (KS) − USA
Ogden (UT) − USA
Wilmington (DE) − USA
Winston−salem (NC) − USA
Worcester (MA) − USA
Washington (DC) − USA
All
<65 years
65−74 years
>74 years
Relative Risk
Mean Temperature (°C)

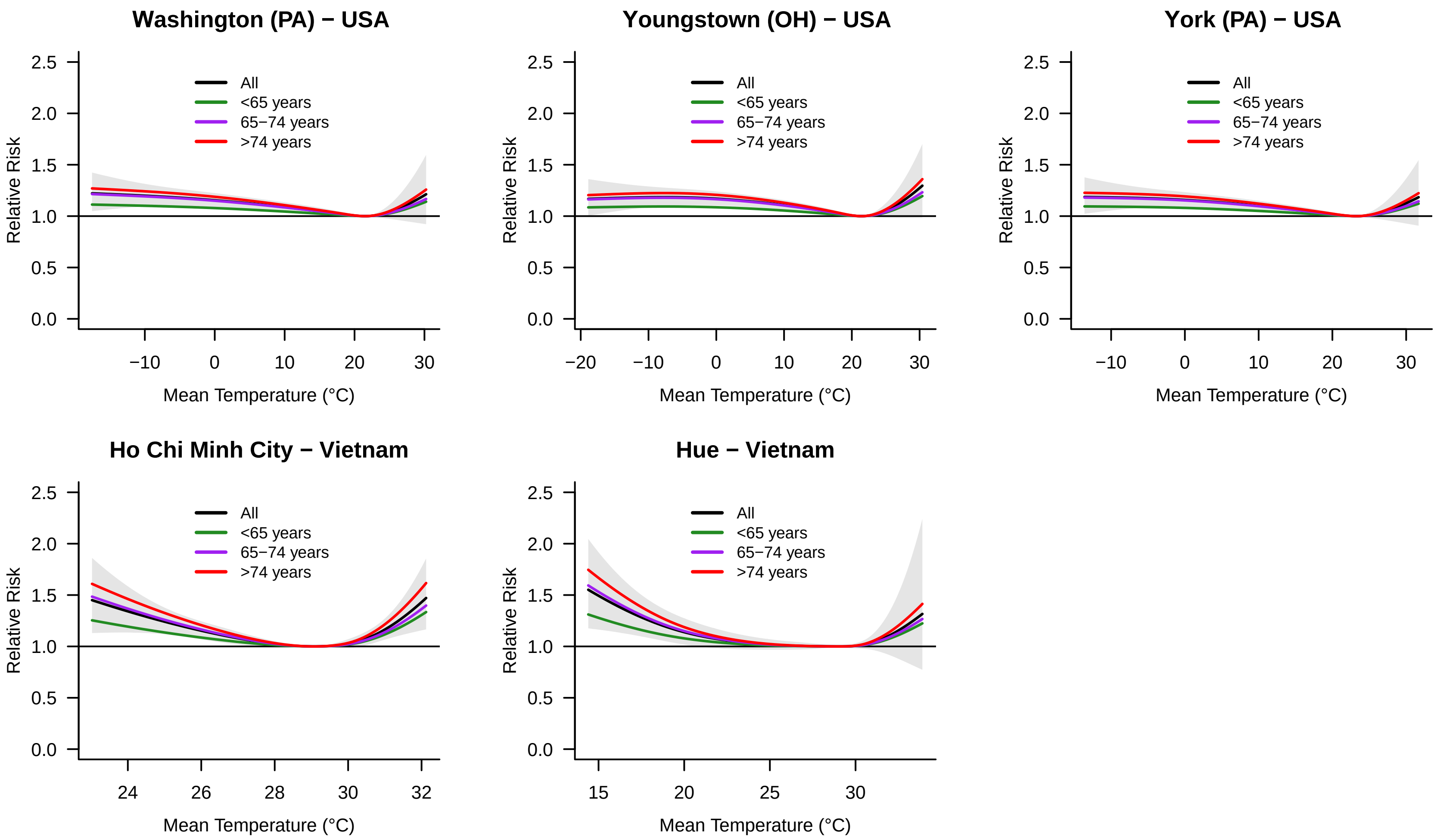


**Supplementary Figure 3.** Temperature-mortality associations by age groups in 800 studied locations. Exposure-response associations are estimated as best linear unbiased predictions (BLUPs) and reported as relative risk (RR) for a cumulative 21-day lag of daily temperature, versus the minimum mortality temperature. Black, solid line represents the association for all age groups (with 95% CI, shaded grey); whereas blue, orange, and red dashed lines represent the association for 0-64, 65-74, and ≥75 years age groups, respectively.

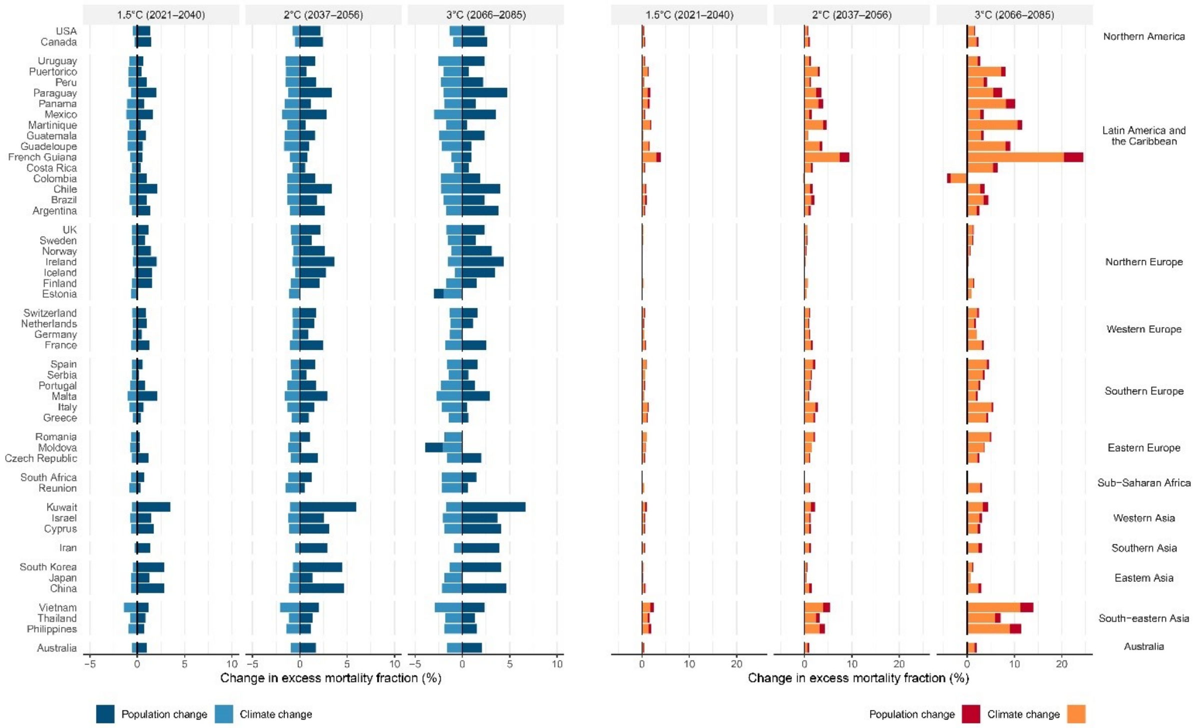


**Supplementary Figure 4.** Contributions of climate change and population change to the changes cold- (A) and heat-related (B) mortality at different levels of global warming under SSP3-7.0. Country/area-level changes by climate change and population aging are shown at 1.5°C, 2°C, and 3°C of global warming using 20-year window compared with the historical period 1995-2014. The future periods in which the 20-year running mean of global mean temperature first reaches the 1.5 °C, 2 °C, and 3 °C of warming above pre-industrial level (1850-1900) are 2021-2040, 2037–2056, 2066–2085, respectively under SSP3-7.0.

**Supplementary Table 1.** Description of the observed temperature and mortality data in the MCC locations.

| Country/ area | N locations | Period | Mortality data | Temperature data | Notes |
|---|---|---|---|---|---|
| *Argentina* | 3 cities | 2005-2014 | Non-external causes only (ICD-9: 0-799; ICD-10: A00-R99) from National Ministry of Health. | Mean daily temperature (in ˚C), computed as the 24-hour average based on hourly measurements from one meteorological station in each city provided by the National Weather Service. | Missing data amount for 0.91% and 0.00% of the mortality and temperature series, respectively. |
| *Australia* | 3 cities | 1995-2009 | Non-external causes only (ICD-9: 0-799; ICD-10: A00-R99) from Australian Bureau of Statistics. | Mean daily temperature (in ˚C), computed as the 24-hour average based on hourly measurements from meteorological stations located within ≤30 km of each city provided by Australian Bureau of Meteorology. | Missing data amount for 0.18% and 0.00% of the mortality and temperature series, respectively. |
| *Brazil* | 18 cities | 1997-2014 | Non-external causes only (ICD-9: 0-799; ICD-10: A00-R99) from the Ministry of Health. | Mean daily temperature (in ˚C), computed from the 24-h average of hourly measurements, from weather stations located within the urban area provided by National Institute of Meteorology of Brazil | Missing data amount for 1.85% and 3.21% of the mortality and temperature series, respectively. |
| *Canada* | 25 census metropolitan areas (CMA) and 1 city (Hamilton) | 1995-2014 | All causes collected from Canadian Mortality Database. | Mean daily temperature (in ˚C), computed as the 24-hour average based on hourly measurements, were obtained from Environment Canada collected from monitoring stations located closest to the CMA centre. | Missing data amount for 0.82% and 2.79% of the mortality and temperature series, respectively. |
| *Chile* | 4 cities | 2004-2014 | All causes provided by the Departamento de Estadísticas e Información de Salud (Ministerio de Salud)) | Mean daily temperature (in ˚C), computed as 24-hour average based on hourly measurements, were obtained from Sistema de Información Nacional de Calidad del Aire (SINCA), Ministerio del Medio Ambiente. | Missing data amount for 0.15% and 9.7% of the mortality and temperature series, respectively. |
| *China* | 14 cities | 1996-2014 | Non-external causes only (ICD-9: 0-799; ICD-10: A00-R99) from Municipal Center for | Mean daily temperature (in ˚C), computed as averaged hourly temperatures, were obtained from China Meteorological Data Sharing Service System (http:// data.cma.cn/). | Missing data amount for 6.98% and 7.35% of the mortality and temperature series, respectively. |

| | | | | | |
|---|---|---|---|---|---|
| | | | Disease Control and Prevention in each city. | | Data on 17 cities were originally collected, but we excluded 3 cities (Tangshan, Nanjing, Guangzhou) because of no data on non-external or suspected errors in data collection. |
| *Colombia* | 5 cities | 1998-2013 | All causes provided by the National Administrative Department of Statistics DANE | Mean daily temperature (in ˚C), computed as 24-hour average based on hourly measurements, were obtained from Instituto de Hidrología, Meteorología y Estudios Ambientales de Colombia (IDEAM) | Missing data amount for 0.00% and 3.98% of the mortality and temperature series, respectively. |
| *Costa Rica* | 1 city | 2000-2014 | All causes provided by the Instituto Nacional de Estadística y Censo. Open Access. | Meterological data were obtained from WMO-NOAA (Surface Data Hourly Global, DS3505) | Missing data amount for 0.00% and 0.97% of the mortality and temperature series, respectively. |
| *Cyprus* | 5 cities | 2004-2014 | All causes provided by the Health Monitoring Unit of the Ministry of Health of Cyprus | Meterological data were obtained from the Department of Meteorology, Ministry of Agriculture, Rural Development, and the Environment. | Missing data amount for 0.0% and 0.0% of the mortality and temperature series, respectively. |
| *Czech Republic* | 3 cities and 1 rural region | 1995-2014 | All causes provided by the Czech Statistical Office and the Institute of Health Information and Statistics | Meteorological data (temperature) were obtained from stations operated by the Czech Hydrometeorological Institute (measurements in standard climatic terms 7:00, 14:00 and 21:00 local time, and daily means) | Missing data amount for 0.00% and 0.00% of the mortality and temperature series, respectively. |
| *Estonia* | 4 cites and 1 region | 1997-2014 | All causes provided by *Estonian Causes of Death Registry* | Mean daily temperature (in ˚C) were computed as the 24-h average of hourly measurements collected from *Estonian Environment Agency.* | Missing data amount for 0.0% and 0.0% of the mortality and temperature series, respectively. |
| *Finland* | 1 metropolitan area | 1995-2014 | All causes provided by Statistics Finland | Mean daily temperature (in ˚C), Finnish Meteorological Institute. The weather stations around the country were interpolated onto a 10×10 km grid covering the whole of Finland, using a Kriging model. | Missing data amount for 0.00% and 4.88% of the mortality and temperature series, respectively. |
| *France* | 20 cities | 2000-2014 | All causes provided by French National Institute of | Mean daily temperature (in ˚C), computed as the mean of the minimal and maximal | Missing data amount for 0.25% and 0.04% of the mortality and |

| | | | | | |
|---|---|---|---|---|---|
| | | | Health and Medical Research (CepiDC), | temperature, were obtained from the Meteo France. A single weather station was selected for each city. | temperature series, respectively. |
| *French Guiana* | 1 city | 2000-2014 | All causes provided by French National Institute of Health and Medical Research | Mean, Max, Min daily temperature (in ˚C) were obtained from the Météo-France for a reference station. | Missing data amount for 0.00% and 0.00% of the mortality and temperature series, respectively. |
| *Germany* | 15 cities | 1995-2014 | All causes provided by Research Data Centres of the Federation and the Federal States of Germany (Forschungsdatenzentrum der Statistischen Ämter des Bundes und der Länder), | Mean daily temperature (in ˚C), computed as the 24-h average based on hourly measurements, was obtained from the Climate Data Centre of the German National Meteorological Service (Deutscher Wetterdienst). | Missing data amount for 0.00% and 0.00% of the mortality and temperature series, respectively. |
| *Greece* | 1 city | 2001-2010 | All causes provided by Hellenic Statistical Authority | Mean daily temperature (in ˚C) and relative humidity (%) were computed as the 24-h average based on hourly measurements collected from the National observatory of Athens (http://www.noa.gr/) from site "Thisio" located in the city of Athens. | Missing data amount for 0.00% and 7.05% of the mortality and temperature series, respectively. |
| *Guadeloupe* | 1 city | 2000-2014 | All causes provided by French National Institute of Health and Medical Research | Mean, Max, Min daily temperature (in ˚C) were obtained from the Météo-France for a reference station. | Missing data amount for 0.00% and 0.00% of the mortality and temperature series, respectively |
| *Guatemala* | 1 city | 2009-2014 | All causes provided by the Instituto Nacional de Estadística, Unidad de Estadística de Salud. | Temperature data are provided by the Instituto Nacional de Sismología, Vulancología, Meteorología y Hidrología. | Missing data amount for 0.00% and 2.15% of the mortality and temperature series, respectively. |
| *Iceland* | 1 city | 2000-2014 | All causes in individuals 18 years or older provided by the Directorate of Health in Iceland, Causes of Death Register. | Mean, Max, Min daily temperature (in ˚C) were obtained from the databank of the Icelandic meteorological institute 2021. | Missing data amount for 0.00% and 0.00% of the mortality and temperature series, respectively |
| *Iran* | 2 cities | 2002-2014 | All causes provided by the Ferdows organization of Mashhad Municipality | Mean, Max, Min daily temperature (in ˚C), computed as the 24-hour average based on hourly measurements collected from IRAN Meteorological Organization (IRIMO) (http://www.irimo.ir) | Missing data amount for 0.00% and 0.00% of the mortality and temperature series, respectively |

| | | | | | |
|---|---|---|---|---|---|
| *Island of Ireland* | 4 regions covering all Island population (ROI) and 2 in the Northern Ireland (NI)) | 1995-2007 | Non-external causes only (ICD-9: 0-799; ICD-10: A00-R99) provided by Irish Central Statistics Office Northern and Ireland Social Research Agency. | Mean daily temperature (in ˚C), computed as the 24-hour average based on hourly measurements, were obtained from two weather stations for each ROI regions and NI regions from Met Eireann, and the United Kingdom Meteorological Office. | Missing data amount for 0.01% and 0.00% of the mortality and temperature series, respectively |
| *Israel* | 2 cities and 2 districts | 1995-2014 | All causes provided by the Israeli Central Bureau of Statistics. | Mean daily temperature (in ˚C) were obtained from 7 weather stations in Israel. | Missing data amount for 0.00% and 0.38% of the mortality and temperature series, respectively |
| *Italy* | 11 cities | 1987-2010 | All causes provided by the obtained from local mortality registries and from the rapid mortality surveillance system | Mean daily temperature (in ˚C) was computed as the 24-h average based on 6-h measurements obtained from the Meteorological Service of the Italian Air Force. A single weather station was selected for each city, using the airport monitoring station located closest to the city center. | Missing data amount for 1.26% and 2.34% of the mortality and temperature series, respectively. Data on 12 cities were initially collected, but 1 (Rieti) was excluded because of potential problems in data collection (strange temporal patterns). |
| *Japan* | 47 prefectures | 1995-2014 | All causes provided by Ministry of Health, Labour and Welfare. | Weather station located within the urban area of the capital city (Japan Meteorology Agency) | Missing data amount for 0.00% and 0.04% of the mortality and temperature series, respectively |
| *Kuwait* | 1 city | 2000-2014 | Non-external causes only (ICD-9: 0-799; ICD-10: A00-R99) provided by the National Center for Health Information, Ministry of Health, Kuwait | Mean daily temperature (in ºC), computed as the 24-hour average based on hourly measurements from two sources: the Directorate General of Civil Aviation (Kuwait Airport) and Kuwait's Environmental Public Authority. | Missing data amount for 0.00% and 0.00% of the mortality and temperature series, respectively |
| *Malta* | 1 city | 1995-2014 | All causes provided by the Ministry for Health in Malta | Mean daily temperature (in ˚C) was obtained from Malta’s airport station. | Missing data amount for 0.04% and 0.00% of the mortality and temperature series, respectively |
| *Martinique* | 1 city | 2000-2014 | All causes provided by French National Institute of Health and Medical Research | Mean, Max, Min daily temperature (in ˚C) were obtained from the Météo-France for a reference station. | Missing data amount for 0.00% and 0.02% of the mortality and temperature series, respectively |
| *Mexico* | 10 metropolitan areas | 1998-2014 | All causes provided by National Institute of Statistics, Geography and Informatics | Mean daily temperature (in ˚C) was computed as the 24-hour average based on hourly measurements collected through the Servicio Meteorológico Nacional | Missing data amount for 0.00% and 27.03% of the mortality and temperature series, respectively |

| | | | | | |
|---|---|---|---|---|---|
| | | | | (SMN) and the Instituto Nacional de Ecología y Cambio Climático (INECC). | |
| *Moldova* | 4 cities | 2001-2010 | All causes provided by National Centre for Health Management | Mean daily temperature (in ˚C) computed as the average between daily minimum and maximum, were obtained from State Hydrometeorological Service, Moldova. A single weather station was selected for each city | Missing data amount for 0.00% and 0.00% of the mortality and temperature series, respectively |
| *The Netherlands* | 4 regions | 1995-2014 | All causes provided by Statistics Netherlands | Mean daily temperature (in ˚C) was obtained from the Royal Dutch Meteorological Institute (KNMI) as 24-hour average based on hourly measurements | Missing data amount for 0.00% and 0.00% of the mortality and temperature series, respectively |
| *Norway* | 1 city | 1995-2014 | All causes provided by Norwegian Cause of Death registry | Mean daily temperature (in ˚C) based on an observational modeled dataset from the Norwegian Meteorological Institute. | Missing data amount for 2.02% and 3.85% of the mortality and temperature series, respectively |
| *Panama* | 1 city | 2013-2014 | All causes provided by Instituto Nacional de Estadística y Censo, Centro de Información Estadística. | Temperature data are provided by the Empresa de Transmisión Eléctrica, S.A. (ETESA). Open Access. | Missing data amount for 0.00% and 10.66% of the mortality and temperature series, respectively |
| *Paraguay* | 1 city | 2004-2014 | All causes provided by Ministerio de Salud Pública y Bienestar Social, Dirección General de Información Estratégica en Salud, Subsistema de Información de Estadísticas Vitales | Temperature data are obtained from the Global Historical Climatology Network (NOAA/WMO) | Missing data amount for 0.00% and 0.00% of the mortality and temperature series, respectively |
| *Peru* | 18 regions | 2008-2014 | All causes provided by the Peruvian Ministry of Health (MINSA in Spanish) | Mean daily temperature (in ˚C) was obtained from the National Meteorology and Hydrology Service of Peru (SENAMHI in Spanish). A total of 18 weather stations (one station per Region) contributed data to each department series. | Missing data amount for 2.73% and 12.12% of the mortality and temperature series, respectively |
| *Philippines* | 12 cities | 2006-2014 | All causes provided by Philippine Statistics Agency | Mean daily temperature (in ˚C), computed as 24-hour average based on hourly measurements, were obtained from National Oceanic and Atmospheric Administration (NOAA). | Missing data amount for 0.04% and 0.00% of the mortality and temperature series, respectively |

| | | | | | |
|---|---|---|---|---|---|
| *Portugal* | 6 districts | 1995-2014 | All causes provided by Statistics Portugal. | Mean daily temperature (in ˚C) was computed as the 24-hour average based on hourly measurements collected from the National Oceanic and Atmospheric Administration (NOAA) | Missing data amount for 0.00% and 0.00% of the mortality and temperature series, respectively |
| *Puerto Rico* | 1 city | 2009-2014 | All causes provided by Instituto de Estadísticas Vitales de Puerto Rico, Área de Estadísticas Vitales del Departamento de Salud | Temperature data are obtained from the Global Historical Climatology Network (NOAA/WMO) | Missing data amount for 0.00% and 5.02% of the mortality and temperature series, respectively |
| *Reunion* | 1 city | 2000-2014 | All causes provided by French National Institute of Health and Medical Research | Mean, Max, Min daily temperature (in ˚C) were obtained from the Météo-France for a reference station. | Missing data amount for 0.00% and 0.00% of the mortality and temperature series, respectively. |
| *Romania* | 8 cities | 1995-2014 | All causes provided by Romanian National Institute of Statistics | Meteorological data (temperature) were obtained from stations operated by the National Meteorological Administration of Romania (NMA RO) (measurements in standard climatic terms, mean daily) by https://www.ecad.eu/ | Missing data amount for 0.00% and 0.00% of the mortality and temperature series, respectively |
| *Serbia* | 1 city | 1995-2014 | All causes, provided by the Statistical office of the Republic of Serbia | Mean daily temperature (in ˚C), computed as the 24-hour average based on 3-hourly measurements, were obtained from the Integrated Surface Dataset (ISD), National Climatic Data Center (NCDC) of the National Oceanic and Atmospheric Administration (NOAA). | Missing data amount for 0.00% and 0.00% of the mortality and temperature series, respectively |
| *South Africa* | 45 district municipalities | 1997-2013 | All causes provided by Statistics South Africa, which had no role in the design, analysis or interpretation of the study. | Mean daily temperature (in ˚C) was computed as the average between daily minimum and maximum collected from the Agricultural Research Council of South Africa and the National Oceanic and Atmospheric Administration (NOAA). | Missing data amount for 0.00% and 12.27% of the mortality and temperature series, respectively<br><br>7 locations were excluded because of a high % of missing data or unstable temporal patterns in the mortality data, possibly due to problems with data collection. |

| | | | | | |
|---|---|---|---|---|---|
| *South Korea* | 36 cities | 1997-2018 | All causes provided by Korea Bureau of Statistics | Mean daily temperature (in ˚C, computed as the 24-hour average based on hourly measurements, were obtained from weather stations located within the urban area managed by Korea Meteorological Administration. | Missing data amount for 0.00% and 0.01% of the mortality and temperature series, respectively |
| *Spain* | 52 cities | 1995-2014 | Non-external causes (ICD-9: 0-799; ICD-10: A00-R99) from the Spain National Institute of Statistics. | Mean daily temperature (in ˚C), computed as the 24-hour average based on hourly measurements, and was obtained from weather stations of the Spain National Meteorology Agency. A single weather station, located within the urban area or at the near airport, was selected for each city | Missing data amount for 0.00% and 0.84% of the mortality and temperature series, respectively |
| *Sweden* | 3 cities | 1995-2014 | All causes provided by the Swedish Cause of Death Register at the Swedish National Board of Health and Welfare | Mean daily temperature (in ˚C, computed as the 24-hour average based on hourly measurements, were obtained from the Environment and Health Administration. | Missing data amount for 0.00% and 2.06% of the mortality and temperature series, respectively |
| *Switzerland* | 7 cities and 1 metropolitan area (Lugano) | 1995-2013 | Non-external causes only other than accidents (ICD-10codes A00-R99, V01-V99, W00-X59) provided from Federal Office of Statistics (Switzerland) | Mean daily temperature (in ˚C), computed as the 24-hour average based on hourly measurements, were obtained from the IDAWEB database (a service provided by MeteoSwiss, the Swiss Federal Office of Meteorology and Climatology). A single weather station located within or near the urban area was selected for each city. | Missing data amount for 0.0% and 0.0% of the mortality and temperature series, respectively. |
| *Thailand* | 61 regions | 1999-2008 | Non-external (ICD-9: 0-799; ICD-10: A00-R99) mortality, provided the Ministry of Public Health, Thailand. | Mean daily temperature (in ˚C), computed as the average between daily minimum and maximum, were obtained from the Meteorological Department, Ministry of Information and Communication Technology, Thailand. | Missing data amount for 0.00% and 4.99% of the mortality and temperature series, respectively. The region of Phetchabun was excluded because of high percentage of missing data. |
| *UK* | 112 built-up areas | 1995-2014 | All causes provided by the Office of National Statistics. | Mean daily temperature (in ˚C) was computed as the 24-hour average based on hourly measurements from UKCP09 5kmx5km product | Missing data amount for 0.00% and 0.00% of the mortality and temperature series, respectively |
| *Uruguay* | 1 city | 2012-2014 | Non-external causes are provided by the Ministerio de Salud Publica (MSP). | Temperature data are provided by the Instituto Uruguayo de Meteorología (INUMET) | Missing data amount for 0.00% and 0.00% of the mortality and temperature series, respectively |

| | | | | | |
|---|---|---|---|---|---|
| *USA* | 210 cities | 1995-2006 | All causes provided by the National Center for Health Statistics (NCHS). | Mean daily temperature (in ˚C), computed as the 24-hour average based on hourly measurements, were obtained from the National Climatic Data Center (NCDC) of the National Oceanic and Atmospheric Administration (NOAA). | Missing data amount for 2.65% and 2.70% of the mortality and temperature series, respectively.<br><br>1 city was excluded (Nampa) because of high percentage of missing. |
| *Vietnam* | 2 cities | 2009-2013 | All causes provided by Provincial Department of Health. | Mean daily temperature (in ˚C), computed as computed from the 24-h average of hourly measurements, were obtained from National Oceanic and Atmospheric Administration's (NOAA) National Climate Data Center (NCDC). A single weather station was selected for each city. | Missing data amount for 0.00% and 0.57% of the mortality and temperature series, respectively |

**Supplementary Table 2.** Descriptive statistics of observation data by region and country/area.

| Country | N of cities | Period | Total deaths | Daily mean temperature (°C) [mean (min-max)] |
|---|---|---|---|---|
| **Northern America** | | | | |
| USA | 210 | 1995 - 2006 | 14,582,845 | 14.1 (3.4-25.0) |
| Canada | 26 | 1995 - 2014 | 2,590,214 | 7.0 (2.5-10.9) |
| **Latin America and the Caribbean** | | | | |
| Guadeloupe | 1 | 2000 - 2014 | 24,492 | 26.6 |
| Martinique | 1 | 2000 - 2014 | 18,641 | 27.0 |
| Puerto Rico | 1 | 2009 - 2014 | 20,192 | 26.7 |
| Costa Rica | 1 | 2000 - 2014 | 25,337 | 22.6 |
| Guatemala | 1 | 2009 - 2014 | 47,939 | 19.3 |
| Mexico | 10 | 1998 - 2014 | 2,980,086 | 18.8 (13.9-23.3) |
| Panama | 1 | 2013 - 2014 | 5,779 | 28.0 |
| Argentina | 3 | 2005 - 2014 | 623,834 | 18.1 (17.8-18.5) |
| Brazil | 18 | 1997 - 2014 | 3,079,875 | 23.3 (18.8-27.8) |
| Chile | 4 | 2004 - 2014 | 325,462 | 13.7 (11.5-15.4) |
| Colombia | 5 | 1998 - 2013 | 956,539 | 23.4 (13.9-28.0) |
| French Guiana | 1 | 2000 - 2014 | 6,592 | 27.0 |
| Paraguay | 1 | 2004 - 2014 | 33,303 | 23.2 |
| Peru | 18 | 2008 - 2014 | 633,137 | 17.9 (4.6-27.0) |
| Uruguay | 1 | 2012 - 2014 | 91,041 | 18.7 |
| **Northern Europe** | | | | |
| Estonia | 5 | 1997 - 2014 | 137,946 | 6.1 (5.5-6.7) |
| Finland | 1 | 1995 - 2014 | 146,417 | 6.3 |
| Iceland | 1 | 2000 - 2014 | 19,182 | 5.4 |
| Ireland | 6 | 1995 - 2007 | 557,877 | 10.0 (9.5-10.9) |
| Norway | 1 | 1995 - 2014 | 96,765 | 5.1 |
| Sweden | 3 | 1995 - 2014 | 533,181 | 8.5 (7.8-8.9) |
| UK | 112 | 1995 - 2014 | 4,448,105 | 10.5 (9.3-11.7) |
| **Western Europe** | | | | |
| France | 20 | 2000 - 2014 | 1,693,354 | 12.6 (10.8-16.3) |
| Germany | 15 | 1995 - 2014 | 3,079,580 | 10.2 (9.5-11.0) |
| Netherland | 4 | 1995 - 2014 | 2,754,019 | 10.3 (9.8-10.7) |
| Switzerland | 8 | 1995 - 2013 | 243,638 | 10.4 (8.6-12.9) |
| **Southern Europe** | | | | |
| Cyprus | 5 | 2004 - 2014 | 51,927 | 20.4 (19.9-21.3) |
| Greece | 1 | 2001 - 2010 | 287,969 | 18.7 |
| Italy | 11 | 1995 - 2010 | 613,479 | 15.5 (12.5-18.4) |
| Malta | 1 | 1995 - 2014 | 61,757 | 19.3 |
| Portugal | 6 | 1995 - 2014 | 1,010,888 | 16.3 (15.1-18.2) |
| Spain | 52 | 1995 - 2014 | 2,452,909 | 15.6 (11.1-21.9) |
| **Eastern Europe** | | | | |
| Czech Republic | 4 | 1995 - 2014 | 643,810 | 9.0 (8.2-9.8) |
| Moldova | 4 | 2001 - 2010 | 59,906 | 10.7 (10.2-11.3) |
| Romania | 8 | 1995 - 2014 | 824,327 | 10.7 (8.2-12.6) |
| Serbia | 1 | 1995 - 2014 | 393,574 | 13.0 |
| **Sub-Saharan Africa** | | | | |
| South Africa | 45 | 1997 - 2013 | 7,775,888 | 18.0 (12.4-22.8) |

| Country | N of cities | Period | Total deaths | Daily mean temperature (°C) [mean (min-max)] |
|---|---|---|---|---|
| Reunion | 1 | 2000 - 2014 | 12,946 | 24.5 |
| **Western Asia** | | | | |
| Israel | 4 | 1995 - 2014 | 356,920 | 20.4 (18.4-21.5) |
| Kuwait | 1 | 2000 - 2014 | 63,184 | 27.0 |
| **Southern Asia** | | | | |
| Iran | 2 | 2002 - 2014 | 763,675 | 16.7 (16-17.3) |
| **Eastern Asia** | | | | |
| China | 14 | 1996 - 2014 | 1,026,772 | 14.5 (7.4-23.7) |
| Japan | 47 | 1995 - 2014 | 21,353,321 | 15.5 (9.2-23.3) |
| South Korea | 36 | 1997 - 2014 | 2,445,155 | 13.1 (9.1-17.1) |
| **South-Eastern Asia** | | | | |
| Philippines | 12 | 2006 - 2014 | 408,430 | 28.2 (27.9-28.6) |
| Thailand | 61 | 1999 - 2008 | 1,801,653 | 27.6 (25.1-29.3) |
| Vietnam | 2 | 2009 - 2013 | 108,173 | 27.1 (25.7-28.5) |
| **Australia** | | | | |
| Australia | 3 | 1995 - 2009 | 789,037 | 18.2 (15.9-20.3) |

**Supplementary Table 3.** CMIP6 GCMs used in this study.

| Model Name | Modeling Center | Instituion Id | Nominal Resolution |
|---|---|---|---|
| **ACCES-CM2** | Commonwealth Scientific and Industrial Research Organisation and Australian Research Council Centre of Excellence for Climate System Science, Australia | CSIRO-ARCCSS | 250 km |
| **AWI-CM-1-1-MR** | Alfred Wegener Institute, Helmholtz Centre for Polar and Marine Research, Germany | AWI | 100 km |
| **BCC-CSM2-MR** | Beijing Climate Center, China | BCC | 100 km |
| **CESM2** | National Center for Atmospheric Research, USA | NCAR | 100 km |
| **CNRM-CM6-1** | Centre National de Recherches Meteorologiques and Centre Europeen de Recherche et de Formation Avancee en Calcul Scientifique, France | CNRM-CERFACS | 250 km |
| **CNRM-CM6-1-HR** | | CNRM-CERFACS | 50 km |
| **CNRM-ESM-2-1** | | CNRM-CERFACS | 250 km |
| **GFDL-ESM4** | National Oceanic and Atmospheric Administration, Geophysical Fluid Dynamics Laboratory, USA | NOAA-GFDL | 100 km |
| **IITEM-ESM** | Centre for Climate Change Research, Indian Institute of Tropical Meteorology Pune, India | CCCR-IITM | 250 km |
| **INM-CM4-8** | Institute for Numerical Mathematics, Russian Academy of Science, Russia | INM | 100 km |
| **INM-CM5-0** | | INM | 100 km |
| **IPSL-CM6A-LR** | Institut Pierre Simon Laplace, France | IPSL | 250 km |
| **MIROC6** | Japan Agency for Marine-Earth Science and Technology; Atmosphere and Ocean Research Institute, The University of Tokyo; National Institute for Environmental Studies; and RIKEN Center for Computational Science; Japan | MIROC | 250 km |
| **MIROC-ES2L** | | MIROC | 500 km |
| **MPI-ESM1-2-LR** | Max Planck Institute for Meteorology, Germany | MPI-M | 250 km |
| **MRI-ESM2-0** | Meteorological Research Institute, Japan | MRI | 100 km |
| **NORESM2-MM** | NorESM Climate modeling Consortium consisting of CICERO (Center for International Climate and Environmental Researc), MET-Norway (Norwegian Meteorological Institute), NERSC (Nansen Environmental and Remote Sensing Center), NILU (Norwegian Institute for Air Research), UiB (University of Bergen), UiO (University of Oslo) and UNI (Uni Research), Norway. | NCC | 100 km |
| **UKESM1-0-LL** | Met Office Hadley Centre, UK | MOHC | 250 km |

**Supplementary Table 4.** Projected changes (95% eCIs) in cold- and heat-related excess mortality (%) at different levels of global warming under SSP5-8.5 by country/area under the "climate only" scenario.

| Country/area | Changes in cold-related mortality (%) | | | Changes in heat-related mortality (%) | | |
|---|---|---|---|---|---|---|
| | 1.5 °C | 2 °C | 3 °C | 1.5 °C | 2 °C | 3 °C |
| **Overall** | -0.6 (-1.0 -0.3) | -1.1 (-1.6, -0.6) | -1.7 (-2.6, -1.0) | 0.4 (0.0, 0.8) | 0.8 (0.0, 1.7) | 1.8 (-0.5, 4.5) |
| **Australia** | | | | | | |
| Australia | -0.5 (-1.0, -0.1) | -0.9 (-1.6, -0.4) | -1.6 (-2.8, -0.6) | 0.3 (0.1, 0.7) | 0.6 (0.2, 1.3) | 1.5 (0.6, 3.3) |
| **Eastern Asia** | | | | | | |
| China | -0.8 (-1.3, -0.4) | -1.3 (-2.0, -0.7) | -2.3 (-3.3, -1.3) | 0.5 (0.1, 1.2) | 1.1 (0.3, 2.2) | 2.6 (0.6, 5.5) |
| Japan | -0.6 (-1.2, -0.2) | -1.1 (-1.7, -0.6) | -1.9 (-2.8, -1.1) | 0.2 (0.0, 0.5) | 0.3 (-0.1, 0.9) | 0.7 (-0.2, 1.9) |
| South Korea | -0.5 (-0.9, -0.2) | -0.8 (-1.3, -0.4) | -1.4 (-2.2, -0.6) | 0.2 (0.0, 0.7) | 0.5 (0.0, 1.1) | 1.1 (0.0, 2.7) |
| **Eastern Europe** | | | | | | |
| Czech Republic | -0.6 (-1.0, -0.2) | -0.9 (-1.6, -0.5) | -1.6 (-2.7, -0.9) | 0.5 (0.1, 0.9) | 1.0 (0.3, 1.9) | 2.2 (0.9, 4.8) |
| Moldova | -0.7 (-1.2, -0.2) | -1.2 (-1.8, -0.6) | -2.0 (-3.3, -1.1) | 0.8 (0.2, 1.7) | 1.6 (0.5, 3.0) | 3.6 (1.6, 6.8) |
| Romania | -0.6 (-1.0, -0.2) | -1.0 (-1.6, -0.6) | -1.8 (-3.1, -1.0) | 1.1 (0.4, 1.8) | 2.2 (0.8, 3.4) | 4.8 (2.4, 7.9) |
| Serbia | -0.6 (-0.9, -0.1) | -0.8 (-1.3, -0.4) | -1.4 (-2.6, -0.7) | 0.8 (0.1, 1.5) | 1.5 (0.3, 2.3) | 3.3 (1.3, 5.8) |
| **South America** | | | | | | |
| Costa Rica | -0.6 (-2.2, 1.1) | -0.7 (-3.1, 1.7) | -0.9 (-4.0, 2.5) | 0.5 (-0.7, 2.0) | 1.4 (-1.8, 5.0) | 5.2 (-6.9, 18.7) |
| Guatemala | -0.9 (-2.2, 0.1) | -1.5 (-3.6, 0.1) | -2.4 (-5.6, 0.4) | 0.2 (-0.4, 0.9) | 0.6 (-1.2, 2.7) | 2.5 (-4.4, 10.3) |
| Mexico | -1.1 (-1.9, -0.6) | -1.9 (-2.6, -1.3) | -3.0 (-4.1, -2.1) | 0.5 (0.1, 0.9) | 1.0 (0.3, 1.8) | 2.7 (0.6, 5.0) |
| Panama | -1.1 (-3.0, 0.4) | -1.5 (-4.4, 0.6) | -2.0 (-5.5, 0.9) | 1.1 (-1.3, 3.4) | 2.7 (-3.7, 7.7) | 7.9 (-15.1, 25.0) |
| Argentina | -0.6 (-1.0, 0.0) | -0.9 (-1.6, -0.4) | -1.8 (-2.8, -1.0) | 0.4 (0.0, 0.9) | 0.8 (0.3, 1.5) | 1.9 (0.7, 3.8) |
| Brazil | -0.7 (-1.4, 0.0) | -1.2 (-2.2, -0.5) | -2.0 (-3.1, -1.0) | 0.7 (-0.1, 1.4) | 1.5 (0.2, 3.3) | 3.5 (-0.2, 8.6) |
| Chile | -0.7 (-1.4, -0.2) | -1.2 (-2.3, -0.6) | -2.2 (-3.8, -1.1) | 0.5 (0.2, 1.0) | 1.1 (0.4, 2.1) | 2.8 (0.9, 5.2) |
| Colombia | -0.7 (-1.6, 0.0) | -1.3 (-2.8, -0.1) | -2.2 (-4.4, -0.2) | 0.0 (-1.7, 1.1) | -0.3 (-6.4, 2.8) | -3.4 (-38.6, 10.5) |
| French Guiana | -0.8 (-2.2, 0.6) | -1.0 (-2.9, 0.9) | -1.2 (-3.4, 1.1) | 2.8 (-4.0, 9.7) | 6.9 (-11.8, 20.5) | 19.3 (-45.4, 53.9) |
| Paraguay | -0.7 (-1.6, -0.2) | -1.2 (-2.7, -0.4) | -2.1 (-3.7, -0.8) | 1.2 (0.0, 4.0) | 2.4 (0.3, 7.6) | 5.6 (1.1, 11.9) |
| Peru | -0.9 (-2.0, 0.0) | -1.5 (-3.1, 0.0) | -2.3 (-4.8, 0.1) | 0.3 (-0.9, 1.5) | 1.0 (-2.6, 4.1) | 3.3 (-9.8, 13.4) |
| Uruguay | -0.8 (-1.7, -0.1) | -1.3 (-2.8, -0.5) | -2.5 (-4.2, -1.5) | 0.4 (0.1, 1.2) | 0.9 (0.4, 1.7) | 2.2 (0.8, 4.6) |
| Guadeloupe | -1.1 (-2.2, -0.2) | -1.6 (-3.1, -0.3) | -2.2 (-4.3, -0.2) | 1.3 (-1.0, 3.9) | 2.9 (-2.9, 8.1) | 8.1 (-10.5, 22.5) |

| Country/area | Changes in cold-related mortality (%) | | | Changes in heat-related mortality (%) | | |
|---|---|---|---|---|---|---|
| | 1.5 °C | 2 °C | 3 °C | 1.5 °C | 2 °C | 3 °C |
| Martinique | -0.9 (-2.1, 0.1) | -1.2 (-2.9, 0.2) | -1.7 (-3.9, 0.6) | 1.6 (-0.5, 4.3) | 3.8 (-1.4, 8.9) | 10.5 (-5.6, 25.7) |
| Puerto Rico | -0.9 (-2.1, 0.0) | -1.4 (-3.2, 0.1) | -2.0 (-4.6, 0.3) | 1.2 (-0.6, 3.2) | 2.6 (-1.6, 6.8) | 7.1 (-6.2, 18.5) |
| **Northern America** | | | | | | |
| Canada | -0.3 (-0.6, -0.1) | -0.6 (-1.0, -0.2) | -1.0 (-1.8, -0.4) | 0.4 (0.1, 0.9) | 0.8 (0.2, 1.9) | 2.0 (0.5, 4.8) |
| USA | -0.5 (-0.8, -0.2) | -0.8 (-1.3, -0.4) | -1.4 (-2.1, -0.8) | 0.3 (-0.1, 0.8) | 0.6 (-0.2, 1.5) | 1.4 (-0.4, 3.6) |
| **Northern Europe** | | | | | | |
| Estonia | -0.7 (-1.3, -0.2) | -1.1 (-2.2, -0.5) | -1.9 (-3.5, -0.9) | 0.2 (-0.1, 0.7) | 0.4 (-0.1, 1.3) | 0.8 (-0.3, 2.8) |
| Finland | -0.6 (-1.2, -0.1) | -0.9 (-1.9, -0.4) | -1.6 (-3.1, -0.6) | 0.3 (-0.2, 0.9) | 0.6 (0.0, 1.6) | 1.3 (0.2, 3.8) |
| Iceland | -0.3 (-1.2, 0.5) | -0.5 (-1.6, 0.3) | -0.8 (-2.5, 0.4) | 0.0 (-0.1, 0.2) | 0.0 (-0.3, 0.4) | 0.1 (-0.7, 1.2) |
| Ireland | -0.4 (-1.2, 0.3) | -0.8 (-1.8, -0.1) | -1.5 (-2.9, -0.1) | 0.1 (-0.2, 0.3) | 0.1 (-0.3, 0.6) | 0.3 (-0.8, 1.8) |
| Norway | -0.4 (-0.9, 0.0) | -0.6 (-1.5, -0.1) | -1.1 (-2.5, -0.2) | 0.1 (-0.2, 0.5) | 0.3 (-0.4, 1.0) | 0.6 (-1.0, 2.5) |
| Sweden | -0.5 (-1.1, -0.1) | -0.9 (-1.7, -0.4) | -1.5 (-2.6, -0.6) | 0.3 (-0.1, 0.6) | 0.5 (0.1, 1.2) | 1.1 (0.2, 2.9) |
| UK | -0.5 (-1.1, 0.0) | -1.0 (-1.7, -0.4) | -1.6 (-2.9, -0.8) | 0.2 (-0.2, 0.7) | 0.5 (-0.1, 1.2) | 1.2 (0.2, 3.4) |
| **South-eastern Asia** | | | | | | |
| Philippines | -0.9 (-1.9, -0.2) | -1.4 (-2.7, -0.2) | -1.9 (-3.6, -0.3) | 1.3 (0.3, 2.7) | 3.1 (0.4, 6.3) | 8.9 (-0.7, 20.5) |
| Thailand | -0.7 (-1.4, -0.1) | -1.2 (-2.1, -0.5) | -1.9 (-3.0, -0.9) | 1 (-0.2, 2.9) | 2.3 (-0.2, 5.8) | 6 (-1.8, 17.4) |
| Vietnam | -1.4 (-2.6, -0.5) | -2.1 (-3.8, -0.8) | -2.9 (-5.0, -1.0) | 1.7 (0.4, 3.4) | 3.9 (0.9, 8.0) | 11 (1.3, 26.8) |
| **Sub-Saharan Africa** | | | | | | |
| South Africa | -0.6 (-1.1, -0.3) | -1.1 (-1.8, -0.6) | -2.1 (-3.4, -1.2) | 0 (-0.4, 0.4) | 0 (-0.9, 0.8) | -0.2 (-3.9, 2) |
| Reunion | -0.8 (-1.9, -0.1) | -1.4 (-2.8, -0.1) | -2.1 (-4.2, 0.0) | 0.4 (-0.6, 1.4) | 0.9 (-1.7, 3.3) | 2.7 (-5.9, 9.9) |
| **Southern Asia** | | | | | | |
| Iran | -0.3 (-0.5, -0.1) | -0.5 (-0.9, -0.2) | -0.9 (-1.5, -0.3) | 0.5 (0.0, 1.0) | 1.0 (0.2, 2.0) | 2.5 (0.6, 4.7) |
| **Southern Europe** | | | | | | |
| Cyprus | -0.7 (-1.4, -0.1) | -1.1 (-1.9, -0.4) | -1.9 (-3.3, -0.8) | 0.5 (-0.1, 1.2) | 1.0 (-0.2, 2.4) | 2.4 (-0.4, 5.4) |
| Greece | -0.5 (-1.0, -0.2) | -0.8 (-1.4, -0.4) | -1.4 (-2.4, -0.6) | 1.0 (0.3, 2.4) | 1.9 (0.7, 3.8) | 4.1 (1.8, 7.2) |
| Italy | -0.8 (-1.3, -0.2) | -1.3 (-1.9, -0.6) | -2.1 (-3.3, -1.2) | 1.3 (0.6, 2.7) | 2.6 (1.0, 3.9) | 5.3 (2.8, 9.1) |
| Malta | -1.0 (-1.8, -0.3) | -1.6 (-2.5, -0.7) | -2.7 (-4.3, -1.3) | 0.4 (0.0, 0.9) | 0.8 (0.1, 1.7) | 1.9 (0.2, 3.9) |
| Portugal | -0.7 (-1.3, -0.3) | -1.3 (-2.0, -0.6) | -2.2 (-3.5, -1.3) | 0.5 (-0.1, 1.2) | 1.0 (0.0, 1.7) | 2.4 (1.0, 3.8) |

| **Country/area** | **Changes in cold-related mortality (%)** | | | **Changes in heat-related mortality (%)** | | |
|---|---|---|---|---|---|---|
| | **1.5 °C** | **2 °C** | **3 °C** | **1.5 °C** | **2 °C** | **3 °C** |
| Spain | -0.6 (-1.0, -0.2) | -0.9 (-1.5, -0.4) | -1.6 (-2.5, -0.9) | 0.9 (0.4, 1.7) | 1.8 (1.0, 2.8) | 4.2 (2.4, 6.3) |
| **Western Asia** | | | | | | |
| Israel | -0.7 (-1.4, 0.1) | -1.2 (-1.9, -0.5) | -2.1 (-3.2, -1.1) | 0.5 (-0.1, 1.2) | 1.2 (-0.2, 2.5) | 2.8 (-0.3, 6.0) |
| Kuwait | -0.6 (-1.1, -0.1) | -1.0 (-1.7, -0.3) | -1.8 (-3.0, -0.7) | 0.7 (-0.3, 1.8) | 1.5 (-0.7, 3.6) | 3.4 (-1.6, 7.7) |
| **Western Europe** | | | | | | |
| France | -0.6 (-1.1, -0.1) | -1.0 (-1.7, -0.5) | -1.8 (-2.9, -1.0) | 0.7 (0.2, 1.2) | 1.4 (0.6, 2.3) | 3.2 (1.5, 6.2) |
| Germany | -0.5 (-0.9, -0.1) | -0.7 (-1.3, -0.4) | -1.2 (-2.1, -0.6) | 0.4 (0.0, 0.9) | 0.9 (0.3, 1.8) | 2.1 (0.8, 4.7) |
| Netherland | -0.4 (-0.9, 0.0) | -0.7 (-1.3, -0.4) | -1.2 (-2.1, -0.5) | 0.4 (0.0, 0.8) | 0.7 (0.1, 1.4) | 1.6 (0.6, 3.5) |
| Switzerland | -0.5 (-0.9, -0.1) | -0.8 (-1.4, -0.3) | -1.3 (-2.4, -0.5) | 0.5 (0.0, 1.1) | 1.0 (0.0, 2.2) | 2.2 (0.0, 5.3) |

**Supplementary Table 5.** Projected changes (95% eCIs) in cold- and heat-related excess mortality (%) at different levels of global warming under SSP5-8.5 by country/area under the "climate-population" scenario.

| Country/area | Changes in cold-related mortality (%) | | | Changes in heat-related mortality (%) | | |
|---|---|---|---|---|---|---|
| | 1.5 °C | 2 °C | 3 °C | 1.5 °C | 2 °C | 3 °C |
| **Overall** | 0.1 (-0.2, 0.4) | 0.4 (0.0, 0.8) | 0.2 (-0.7, 0.8) | 0.5 (0.0, 1.0) | 1.0 (0.0, 2.2) | 2.5 (-1.3, 5.9) |
| **Australia** | | | | | | |
| Australia | 0.1 (-0.4, 0.6) | 0.4 (-0.4, 1.3) | 0.3 (-0.8, 1.6) | 0.4 (0.1, 0.9) | 0.9 (0.3, 1.7) | 2.0 (0.8, 4.1) |
| **Eastern Asia** | | | | | | |
| China | 1.0 (0.5, 1.6) | 2.3 (1.4, 3.3) | 2.3 (1.2, 3.5) | 0.7 (0.1, 1.5) | 1.6 (0.4, 3.0) | 3.5 (0.9, 6.9) |
| Japan | 0.1 (-0.5, 0.5) | -0.2 (-0.8, 0.2) | -1.7 (-2.6, -0.9) | 0.2 (0.0, 0.6) | 0.4 (-0.1, 1.0) | 0.8 (-0.2, 2.1) |
| South Korea | 1.5 (0.9, 2.0) | 2.7 (1.7, 3.7) | 3.1 (2.0, 4.2) | 0.3 (0.0, 0.8) | 0.7 (0.0, 1.5) | 1.4 (0.0, 3.4) |
| **Eastern Europe** | | | | | | |
| Czech Republic | -0.2 (-0.6, 0.2) | 0.3 (-0.3, 0.7) | 0.7 (-0.5, 1.6) | 0.5 (0.1, 1.0) | 1.2 (0.5, 2.2) | 2.7 (1.2, 5.7) |
| Moldova | -1.4 (-2.0, -0.7) | -2.1 (-3.1, -1.3) | -6.9 (-9.5, -4.4) | 0.8 (0.1, 1.6) | 1.7 (0.5, 3.1) | 3.7 (1.6, 7.1) |
| Romania | -1.2 (-1.7, -0.7) | -1.4 (-2.0, -0.8) | -1.4 (-2.9, -0.6) | 1.0 (0.3, 1.8) | 2.3 (0.8, 3.5) | 5.3 (2.8, 8.7) |
| Serbia | -0.9 (-1.4, -0.4) | -0.7 (-1.2, -0.2) | -0.6 (-1.8, 0.2) | 0.7 (0.0, 1.5) | 1.6 (0.3, 2.4) | 3.9 (1.6, 6.6) |
| **Latin America and the Caribbean** | | | | | | |
| Costa Rica | -0.3 (-1.3, 0.5) | -0.3 (-1.3, 0.6) | -0.3 (-1.4, 0.8) | 0.6 (-0.9, 2.6) | 1.8 (-2.5, 6.3) | 6.5 (-9.2, 22.3) |
| Guatemala | -0.5 (-1.6, 0.4) | -0.8 (-2.2, 0.4) | -1.2 (-3.1, 0.4) | 0.2 (-0.4, 1.1) | 0.7 (-1.5, 3.2) | 3.3 (-6.2, 12.9) |
| Mexico | -0.2 (-0.9, 0.3) | 0 (-0.7, 0.6) | -0.5 (-1.6, 0.3) | 0.6 (0.2, 1.1) | 1.4 (0.4, 2.4) | 3.6 (0.8, 6.4) |
| Panama | -0.7 (-2.0, 0.4) | -0.7 (-2.3, 0.3) | -0.8 (-2.4, 0.4) | 1.4 (-1.7, 4.1) | 3.6 (-5.2, 9.8) | 10.2 (-21.6, 29.9) |
| Argentina | 0.0 (-0.3, 0.6) | 0.5 (-0.2, 1.1) | 0.6 (-0.3, 1.5) | 0.5 (0.0, 1.0) | 1.1 (0.5, 1.8) | 2.5 (1.1, 4.6) |
| Brazil | -0.1 (-0.8, 0.7) | 0.0 (-1.0, 0.8) | -0.2 (-1.1, 0.8) | 0.9 (0.0, 1.7) | 2.0 (0.4, 4.2) | 4.8 (-0.2, 10.9) |
| Chile | 0.6 (-0.1, 1.3) | 1.4 (0.1, 2.6) | 1.3 (-0.4, 3.0) | 0.7 (0.2, 1.3) | 1.6 (0.6, 2.9) | 3.8 (1.3, 6.7) |
| Colombia | -0.1 (-0.9, 0.6) | -0.1 (-1.3, 1.0) | -0.8 (-2.1, 0.4) | 0.1 (-2.1, 1.4) | -0.3 (-9.4, 3.7) | -4.7 (-66.5, 13.9) |
| French Guiana | -0.4 (-1.4, 0.3) | -0.4 (-1.2, 0.3) | -0.4 (-1.1, 0.3) | 3.7 (-5.3, 11.5) | 9.3 (-17.8, 25) | 24.5 (-73.6, 61.1) |
| Paraguay | 0.5 (-0.4, 1.3) | 1.2 (-0.3, 2.5) | 1.5 (0.1, 3.6) | 1.6 (0.3, 4.6) | 3.3 (1.0, 8.7) | 7.6 (2.3, 14.6) |
| Peru | -0.4 (-1.2, 0.2) | -0.4 (-1.5, 0.4) | -0.9 (-2.6, 0.3) | 0.4 (-1.1, 1.7) | 1.2 (-3.5, 4.9) | 4.2 (-14.7, 16.2) |
| Uruguay | -1.0 (-1.9, -0.3) | -1.1 (-2.7, -0.4) | -2.2 (-4.0, -1.2) | 0.4 (0.1, 1.1) | 1.0 (0.5, 1.8) | 2.5 (1.0, 5.1) |
| Guadeloupe | -0.8 (-1.8, -0.2) | -1.0 (-2.1, -0.2) | -1.4 (-2.8, -0.2) | 1.5 (-1.2, 4.3) | 3.6 (-3.5, 9.4) | 9.5 (-12.8, 24.9) |
| Martinique | -0.7 (-1.8, 0.0) | -0.9 (-2.2, 0.1) | -1.2 (-2.9, 0.3) | 1.8 (-0.5, 4.6) | 4.4 (-1.5, 10.0) | 11.9 (-6.3, 27.7) |

| Country/area | Changes in cold-related mortality (%) | | | Changes in heat-related mortality (%) | | |
|---|---|---|---|---|---|---|
| | **1.5 °C** | **2 °C** | **3 °C** | **1.5 °C** | **2 °C** | **3 °C** |
| Puertorico | -0.8 (-1.8, -0.1) | -1.1 (-2.5, 0.0) | -1.9 (-4.4, 0.3) | 1.3 (-0.7, 3.6) | 3.1 (-1.9, 7.7) | 8.1 (-7.3, 20.3) |
| **Northern America** | | | | | | |
| Canada | 0.5 (0.3, 0.7) | 1.3 (0.8, 1.7) | 1.5 (0.7, 2.0) | 0.5 (0.1, 1.0) | 1.1 (0.3, 2.3) | 2.4 (0.7, 5.6) |
| USA | 0.3 (0.0, 0.6) | 0.9 (0.5, 1.4) | 0.7 (-0.1, 1.2) | 0.4 (-0.1, 0.9) | 0.8 (-0.2, 1.9) | 1.7 (-0.5, 4.3) |
| **Northern Europe** | | | | | | |
| Estonia | -1.3 (-2, -0.7) | -2.1 (-3.4, -1.2) | -3.4 (-5.3, -1.9) | 0.2 (-0.1, 0.7) | 0.4 (-0.1, 1.3) | 0.9 (-0.3, 3.1) |
| Finland | 0.2 (-0.4, 0.8) | 0.7 (-0.3, 1.5) | -0.2 (-1.6, 0.7) | 0.4 (-0.1, 1.0) | 0.7 (0.1, 1.9) | 1.5 (0.3, 4.3) |
| Iceland | 0.5 (-0.5, 1.6) | 1.6 (-0.4, 3.8) | 2.3 (-0.7, 5.5) | 0.0 (-0.2, 0.2) | 0.1 (-0.3, 0.5) | 0.2 (-0.9, 1.4) |
| Ireland | 0.7 (0.0, 1.6) | 1.7 (0.7, 2.9) | 2.6 (1.1, 4.6) | 0.1 (-0.2, 0.4) | 0.2 (-0.4, 0.8) | 0.4 (-1.0, 2.2) |
| Norway | 0.2 (-0.2, 0.6) | 1.2 (0.2, 2.2) | 1.9 (0.2, 3.6) | 0.2 (-0.3, 0.6) | 0.3 (-0.6, 1.3) | 0.8 (-1.3, 3) |
| Sweden | -0.3 (-0.8, 0.1) | -0.1 (-0.8, 0.4) | -0.2 (-1.3, 0.5) | 0.3 (0.0, 0.6) | 0.6 (0.1, 1.3) | 1.3 (0.3, 3.3) |
| UK | -0.2 (-0.8, 0.4) | 0.2 (-0.5, 0.8) | 0.2 (-0.9, 1.2) | 0.3 (-0.2, 0.7) | 0.6 (0.0, 1.3) | 1.4 (0.3, 3.8) |
| **South-eastern Asia** | | | | | | |
| Philippines | -0.5 (-1.2, -0.1) | -0.6 (-1.5, -0.1) | -0.8 (-1.7, -0.2) | 1.8 (0.5, 3.3) | 4.3 (0.7, 7.9) | 12.1 (-1.1, 25.3) |
| Thailand | -0.2 (-0.8, 0.7) | -0.2 (-0.8, 0.7) | -0.6 (-1.5, 0.2) | 1.4 (0.0, 3.5) | 3.2 (-0.1, 7.4) | 7.8 (-2.6, 20.8) |
| Vietnam | -0.8 (-1.8, -0.2) | -0.7 (-1.8, 0.0) | -1.0 (-2.2, 0.5) | 2.1 (0.6, 4.1) | 5.3 (1.4, 10.2) | 14.2 (2.2, 32.0) |
| **Sub-Saharan Africa** | | | | | | |
| South Africa | 0.0 (-0.5, 0.3) | 0.1 (-0.7, 0.7) | 0.4 (-1.1, 1.5) | 0.0 (-0.5, 0.5) | 0.1 (-1.2, 0.9) | -0.3 (-6.6, 2.7) |
| Reunion | -0.6 (-1.6, -0.1) | -0.9 (-2.2, -0.2) | -1.3 (-3.0, -0.2) | 0.5 (-0.8, 1.6) | 1.2 (-2.1, 3.9) | 3.7 (-9.0, 12.3) |
| **Southern Asia** | | | | | | |
| Iran | 0.3 (0.1, 0.5) | 1.4 (0.6, 2.1) | 2.6 (1.1, 3.9) | 0.6 (0.1, 1.2) | 1.4 (0.3, 2.6) | 3.4 (0.9, 6.4) |
| **Southern Europe** | | | | | | |
| Cyprus | 0.2 (-0.4, 0.8) | 1.2 (0.3, 2.1) | 1.8 (0.5, 3.2) | 0.6 (-0.1, 1.4) | 1.3 (-0.2, 3.0) | 3.0 (-0.5, 6.6) |
| Greece | -0.5 (-0.9, -0.1) | -0.5 (-1.0, -0.1) | -0.7 (-1.5, 0.0) | 1.1 (0.3, 2.4) | 2.1 (0.9, 4.1) | 4.6 (2.2, 8.0) |
| Italy | -0.8 (-1.3, -0.2) | -0.8 (-1.4, -0.2) | -0.8 (-2.0, 0.1) | 1.4 (0.6, 2.8) | 2.8 (1.2, 4.3) | 5.9 (3.3, 9.9) |
| Malta | 0.2 (-0.6, 1.0) | 0.8 (-0.3, 1.9) | -0.1 (-1.5, 1.5) | 0.5 (0.0, 1.1) | 1.1 (0.1, 2.1) | 2.3 (0.3, 4.6) |
| Portugal | -0.6 (-1.2, -0.2) | -0.6 (-1.2, 0.1) | -0.7 (-1.9, 0.1) | 0.6 (-0.1, 1.3) | 1.2 (0.1, 2.0) | 2.8 (1.4, 4.4) |

| Country/area | Changes in cold-related mortality (%) | | | Changes in heat-related mortality (%) | | |
|---|---|---|---|---|---|---|
| | **1.5 °C** | **2 °C** | **3 °C** | **1.5 °C** | **2 °C** | **3 °C** |
| Spain | -0.6 (-1.0, -0.2) | -0.3 (-0.7, 0.2) | 0.3 (-0.5, 1.0) | 1.0 (0.4, 1.8) | 2.1 (1.2, 3.2) | 4.8 (2.9, 7.2) |
| **Western Asia** | | | | | | |
| Israel | 0.0 (-0.6, 0.8) | 0.5 (-0.4, 1.4) | 0.5 (-0.8, 1.7) | 0.6 (-0.1, 1.4) | 1.4 (-0.2, 2.9) | 3.4 (-0.4, 6.9) |
| Kuwait | 1.9 (0.0, 3.5) | 4.2 (0.2, 7.5) | 5.1 (-0.1, 9.4) | 1.1 (-0.5, 2.5) | 2.3 (-1, 5.1) | 4.9 (-2.4, 10.5) |
| **Western Europe** | | | | | | |
| France | -0.3 (-0.8, 0.2) | 0.2 (-0.4, 0.8) | 0.1 (-1.0, 0.8) | 0.7 (0.2, 1.3) | 1.6 (0.7, 2.7) | 3.6 (1.8, 6.8) |
| Germany | -0.3 (-0.8, 0.1) | -0.4 (-0.9, -0.1) | -0.7 (-1.6, -0.1) | 0.5 (0.1, 0.9) | 1.1 (0.4, 2.0) | 2.3 (0.9, 5.1) |
| Netherland | 0.1 (-0.4, 0.5) | 0.3 (-0.3, 0.8) | -0.1 (-0.9, 0.7) | 0.5 (0.1, 0.9) | 0.9 (0.3, 1.7) | 1.9 (0.9, 4.1) |
| Switzerland | -0.1 (-0.4, 0.3) | 0.3 (-0.2, 0.7) | 0.3 (-0.6, 1.0) | 0.6 (0.0, 1.3) | 1.2 (0.0, 2.6) | 2.7 (0.0, 6.1) |

**Supplementary Table 6.** Projected changes (95% eCIs) in non-optimal temperature-related excess mortality (%) at different levels of global warming under SSP5-8.5 by country/area under the “climate-population” scenario.

| Country/area | 1.5 °C | 2 °C | 3 °C |
|---|---|---|---|
| **Overall** | 0.5 (0.1 to 0.9) | 1.5 (0.3 to 2.4) | 2.7 (-1.3 to 5.6) |
| **Australia** | | | |
| Australia | 0.5 (0.0, 1.0) | 1.3 (0.3, 2.3) | 2.3 (0.7, 4.1) |
| **Eastern Asia** | | | |
| China | 1.8 (1.0, 2.6) | 3.9 (2.4, 5.3) | 5.7 (3.1, 8.5) |
| Japan | 0.3 (-0.3, 0.7) | 0.1 (-0.5, 0.7) | -0.9 (-2.2, 0.1) |
| South Korea | 1.8 (1.2, 2.4) | 3.4 (2.3, 4.4) | 4.5 (2.9, 6.1) |
| **Eastern Europe** | | | |
| Czech Republic | 0.4 (-0.1, 0.8) | 1.5 (0.7, 2.4) | 3.4 (1.7, 5.5) |
| Moldova | -0.6 (-1.4, 0.1) | -0.5 (-1.7, 0.9) | -3.2 (-5.9, -0.3) |
| Romania | -0.2 (-0.8, 0.5) | 0.9 (-0.2, 2.0) | 3.9 (1.8, 6.1) |
| Serbia | -0.2 (-0.9, 0.5) | 0.9 (-0.1, 1.7) | 3.3 (1.2, 5.2) |
| **Latin America and the Caribbean** | | | |
| Costa Rica | 0.3 (-1.2, 2.0) | 1.6 (-2.6, 5.8) | 6.2 (-9.2, 21.7) |
| Guatemala | -0.3 (-1.5, 0.7) | -0.1 (-2.5, 2.4) | 2.1 (-7.5, 11.2) |
| Mexico | 0.4 (-0.3, 1.1) | 1.4 (0.3, 2.3) | 3.1 (0.4, 5.4) |
| Panama | 0.7 (-2.3, 3.3) | 2.9 (-5.6, 8.9) | 9.4 (-22.2, 28.6) |
| Argentina | 0.5 (-0.1, 1.0) | 1.6 (0.6, 2.5) | 3.1 (1.6, 4.7) |
| Brazil | 0.7 (0.1, 1.4) | 2.0 (0.5, 3.5) | 4.7 (-0.4, 10.1) |
| Chile | 1.3 (0.4, 2.3) | 3.0 (1.3, 4.6) | 5.0 (2.1, 7.9) |
| Colombia | 0.0 (-2.3, 1.2) | -0.4 (-9.8, 3.2) | -5.4 (-67.9, 13.3) |
| French Guiana | 3.2 (-5.7, 10.8) | 8.9 (-17.9, 24.3) | 24.1 (-74, 60.7) |
| Paraguay | 2.1 (0.9, 4.2) | 4.5 (2.2, 8.4) | 9.1 (4.5, 15.2) |
| Peru | 0.0 (-1.6, 1.1) | 0.8 (-4.0, 4.0) | 3.3 (-15.8, 14.8) |
| Uruguay | -0.6 (-1.3, 0.2) | -0.1 (-1.1, 0.7) | 0.3 (-1.2, 2.3) |
| Guadeloupe | 0.7 (-2.1, 3.1) | 2.7 (-4.5, 8.1) | 8.1 (-14.1, 23.1) |
| Martinique | 1.1 (-1.2, 3.6) | 3.5 (-2.3, 8.9) | 10.6 (-7.3, 26.2) |
| Puertorico | 0.6 (-1.4, 2.5) | 2.0 (-2.8, 6.2) | 6.2 (-8.7, 17.8) |
| **Northern America** | | | |
| Canada | 1.0 (0.5, 1.4) | 2.3 (1.4, 3.4) | 3.9 (2.0, 6.6) |
| USA | 0.6 (0.1, 1.1) | 1.7 (0.6, 2.7) | 2.4 (0.1, 4.6) |
| **Northern Europe** | | | |
| Estonia | -1.1 (-1.8, -0.5) | -1.7 (-2.9, -0.8) | -2.5 (-4.4, -0.8) |
| Finland | 0.6 (0.0, 1.1) | 1.4 (0.4, 2.5) | 1.3 (-0.1, 3.1) |
| Iceland | 0.6 (-0.4, 1.6) | 1.7 (-0.3, 3.8) | 2.5 (-0.4, 5.5) |
| Ireland | 0.8 (0.0, 1.7) | 1.9 (0.8, 3.0) | 3.1 (0.9, 4.8) |
| Norway | 0.3 (-0.3, 0.8) | 1.6 (0.3, 2.8) | 2.7 (0.1, 4.9) |
| Sweden | 0 (-0.4, 0.4) | 0.5 (-0.2, 1.2) | 1.1 (-0.1, 2.4) |
| UK | 0.1 (-0.5, 0.5) | 0.7 (0.0, 1.3) | 1.6 (0.4, 3.1) |

| **Country/area** | **1.5 °C** | **2 °C** | **3 °C** |
|---|---|---|---|
| **Overall** | 0.5 (0.1 to 0.9) | 1.5 (0.3 to 2.4) | 2.7 (-1.3 to 5.6) |
| **South-eastern Asia** | | | |
| Philippines | 1.3 (-0.1, 2.6) | 3.7 (0.1, 7.0) | 11.3 (-1.8, 24.3) |
| Thailand | 1.2 (-0.1, 3.0) | 3.1 (-0.3, 6.9) | 7.2 (-3.2, 19.5) |
| Vietnam | 1.4 (-0.1, 3.3) | 4.6 (1.0, 9.0) | 13.2 (2.4, 30.4) |
| **Sub-Saharan Africa** | | | |
| South Africa | 0.0 (-0.7, 0.3) | 0.2 (-1.3, 0.8) | 0.1 (-6.9, 2.3) |
| Reunion | -0.1 (-1.5, 1.0) | 0.2 (-3.0, 2.7) | 2.3 (-10.3, 10.4) |
| **Southern Asia** | | | |
| Iran | 0.8 (0.3, 1.4) | 2.8 (1.4, 4.1) | 6.0 (3.0, 9.0) |
| **Southern Europe** | | | |
| Cyprus | 0.9 (-0.1, 1.8) | 2.5 (0.4, 4.5) | 4.8 (0.6, 8.6) |
| Greece | 0.6 (-0.1, 1.7) | 1.6 (0.5, 3.3) | 3.9 (1.8, 6.8) |
| Italy | 0.6 (-0.1, 1.8) | 2.0 (0.8, 3.2) | 5.1 (2.9, 8.0) |
| Malta | 0.7 (-0.2, 1.6) | 1.8 (0.4, 3.2) | 2.2 (-0.1, 4.3) |
| Portugal | 0.0 (-0.3, 0.3) | 0.6 (0.1, 1.3) | 2.1 (1.2, 3.0) |
| Spain | 0.4 (-0.1, 1.2) | 1.8 (1.0, 2.9) | 5.1 (3.2, 7.3) |
| **Western Asia** | | | |
| Israel | 0.6 (-0.4, 1.5) | 1.9 (-0.1, 3.7) | 3.8 (-0.3, 7.5) |
| Kuwait | 2.9 (0.4, 5.2) | 6.4 (0.9, 11.3) | 10.0 (0.1, 17.7) |
| **Western Europe** | | | |
| France | 0.4 (-0.1, 0.9) | 1.8 (1.1, 2.6) | 3.7 (2.3, 5.9) |
| Germany | 0.1 (-0.4, 0.6) | 0.7 (0.1, 1.4) | 1.6 (0.4, 3.6) |
| Netherland | 0.5 (0.1, 1.0) | 1.3 (0.6, 2.0) | 1.8 (0.9, 3.2) |
| Switzerland | 0.5 (-0.2, 1.2) | 1.5 (0.1, 2.8) | 3.0 (0.1, 6.0) |

**Supplementary Table 7.** Changes (95% eCIs) in non-optimal temperature-related excess mortality (%) due to population aging at different levels of global warming under SSP5-8.5 by country/area under the "climate-population" scenario compared with the "climate-only" scenario.

| Country/area | 1.5 °C | 2 °C | 3 °C |
|---|---|---|---|
| **Overall** | 0.8 (0.6 to 0.9) | 1.7 (1.2 to 2.1) | 2.6 (0.9, 3.5) |
| **Australia** | | | |
| Australia | 0.7 (0.2, 1.2) | 1.5 (0.4, 2.7) | 2.4 (0.6, 4.0) |
| **Eastern Asia** | | | |
| China | 2.0 (1.4, 2.5) | 4.1 (3.0, 5.2) | 5.4 (3.9, 6.7) |
| Japan | 0.7 (0.5, 0.9) | 0.9 (0.7, 1.1) | 0.2 (0.1, 0.3) |
| South Korea | 2.0 (1.4, 2.6) | 3.7 (2.5, 4.7) | 4.8 (3.3, 6.1) |
| **Eastern Europe** | | | |
| Czech Republic | 0.5 (0.3, 0.6) | 1.4 (1.0, 1.8) | 2.8 (2.0, 3.6) |
| Moldova | -0.7 (-1.0, -0.5) | -0.9 (-1.3, -0.5) | -4.8 (-6.5, -3.1) |
| Romania | -0.6 (-0.8, -0.4) | -0.2 (-0.4, -0.1) | 0.9 (0.7, 1.1) |
| Serbia | -0.4 (-0.5, -0.3) | 0.3 (0.2, 0.3) | 1.4 (1.0, 1.8) |
| **Latin America and the Caribbean** | | | |
| Costa Rica | 0.4 (-0.8, 1.5) | 0.9 (-1.5, 3.0) | 1.9 (-3, 5.3) |
| Guatemala | 0.4 (-0.1, 1.0) | 0.9 (-0.3, 2.0) | 2.0 (-1.2, 4.3) |
| Mexico | 1.0 (0.8, 1.3) | 2.2 (1.7, 2.8) | 3.4 (2.4, 4.2) |
| Panama | 0.7 (-0.4, 1.6) | 1.7 (-1.2, 3.8) | 3.5 (-5.8, 7.0) |
| Argentina | 0.7 (0.5, 0.8) | 1.7 (1.3, 2.1) | 3.0 (2.2, 3.7) |
| Brazil | 0.8 (0.5, 1.0) | 1.7 (1.1, 2.2) | 3.1 (1.4, 4.3) |
| Chile | 1.5 (0.7, 2.2) | 3.1 (1.5, 4.5) | 4.4 (2.2, 6.4) |
| Colombia | 0.7 (0.2, 1.0) | 1.2 (-1.3, 1.9) | 0.2 (-25.9, 3.7) |
| French Guiana | 1.2 (-1.2, 2.7) | 3.0 (-5.5, 6.1) | 6.0 (-28.6, 9.5) |
| Paraguay | 1.6 (0.9, 2.3) | 3.3 (1.8, 4.6) | 5.6 (3.2, 7.7) |
| Peru | 0.6 (0.0, 1.1) | 1.3 (-0.2, 2.3) | 2.2 (-3.7, 4.2) |
| Uruguay | -0.2 (-0.3, -0.1) | 0.2 (0.1, 0.3) | 0.6 (0.4, 0.8) |
| Guadeloupe | 0.5 (0.0, 0.9) | 1.3 (-0.3, 2.3) | 2.2 (-1.7, 3.8) |
| Martinique | 0.3 (0.0, 0.6) | 1.0 (-0.1, 1.9) | 1.8 (-0.6, 3.0) |
| Puertorico | 0.3 (-0.1, 0.7) | 0.8 (-0.2, 1.6) | 1.2 (-1.1, 2.0) |
| **Northern America** | | | |
| Canada | 0.9 (0.6, 1.2) | 2.1 (1.4, 2.7) | 2.9 (1.9, 3.9) |
| USA | 0.8 (0.6, 1.0) | 1.9 (1.3, 2.4) | 2.4 (1.6, 3.0) |
| **Northern Europe** | | | |
| Estonia | -0.6 (-0.7, -0.4) | -1.0 (-1.3, -0.7) | -1.4 (-1.8, -0.9) |
| Finland | 0.9 (0.4, 1.3) | 1.8 (0.9, 2.6) | 1.7 (0.8, 2.4) |
| Iceland | 0.8 (0.0, 1.6) | 2.1 (-0.1, 4.1) | 3.1 (-0.2, 6.2) |
| Ireland | 1.2 (0.8, 1.4) | 2.6 (1.9, 3.3) | 4.2 (3.0, 5.2) |
| Norway | 0.6 (0.2, 0.9) | 1.9 (0.7, 3.1) | 3.2 (1.1, 5.1) |
| Sweden | 0.3 (0.2, 0.4) | 0.9 (0.5, 1.2) | 1.4 (0.9, 1.9) |
| UK | 0.4 (0.3, 0.4) | 1.2 (1.0, 1.4) | 2.1 (1.6, 2.5) |

| Country/area | 1.5 °C | 2 °C | 3 °C |
|---|---|---|---|
| **Overall** | 0.8 (0.6 to 0.9) | 1.7 (1.2 to 2.1) | 2.6 (0.9, 3.5) |
| **South-eastern Asia** | | | |
| Philippines | 0.8 (0.3, 1.4) | 1.9 (0.7, 3.1) | 4.3 (0.3, 6.3) |
| Thailand | 0.9 (0.5, 1.4) | 2.0 (0.9, 2.9) | 3.1 (0.3, 4.7) |
| Vietnam | 1.0 (0.5, 1.8) | 2.8 (1.3, 5.1) | 5.1 (2.5, 7.8) |
| **Sub-Saharan Africa** | | | |
| South Africa | 0.5 (0.4, 0.7) | 1.3 (0.9, 1.5) | 2.4 (-0.1, 3.2) |
| Reunion | 0.3 (-0.1, 0.5) | 0.6 (-0.4, 1.3) | 1.7 (-2.8, 3.5) |
| **Southern Asia** | | | |
| Iran | 0.6 (0.3, 0.9) | 2.2 (1.1, 3.2) | 4.4 (2.3, 6.2) |
| **Southern Europe** | | | |
| Cyprus | 1.0 (0.4, 1.6) | 2.6 (1.0, 3.8) | 4.4 (1.7, 6.6) |
| Greece | 0.1 (0.0, 0.1) | 0.5 (0.3, 0.7) | 1.2 (0.7, 1.7) |
| Italy | 0.1 (0.1, 0.1) | 0.7 (0.5, 0.9) | 1.9 (1.3, 2.4) |
| Malta | 1.3 (0.6, 1.9) | 2.6 (1.2, 3.7) | 3.0 (1.4, 4.4) |
| Portugal | 0.2 (0.1, 0.2) | 0.9 (0.6, 1.1) | 1.9 (1.4, 2.4) |
| Spain | 0.0 (0.0, 0.0) | 0.9 (0.6, 1.3) | 2.5 (1.5, 3.4) |
| **Western Asia** | | | |
| Israel | 0.8 (0.3, 1.3) | 1.9 (0.6, 3.1) | 3.1 (1.0, 5.0) |
| Kuwait | 2.7 (0.6, 4.6) | 5.9 (1.2, 10.0) | 8.4 (1.6, 13.9) |
| **Western Europe** | | | |
| France | 0.3 (0.3, 0.4) | 1.5 (1.1, 1.8) | 2.3 (1.8, 2.8) |
| Germany | 0.2 (0.1, 0.2) | 0.5 (0.3, 0.6) | 0.8 (0.5, 1.0) |
| Netherland | 0.6 (0.4, 0.8) | 1.3 (0.8, 1.7) | 1.4 (1.0, 1.9) |
| Switzerland | 0.5 (0.2, 0.7) | 1.3 (0.5, 1.9) | 2.0 (0.9, 3.0) |

## Supplementary References


1 Gasparrini, A., Armstrong, B. & Kenward, M. G. Distributed lag non-linear models. *Stat Med* **29**, 2224-2234 (2010).

2 Gasparrini, A., Armstrong, B. & Kenward, M. G. Multivariate meta-analysis for non-linear and other multi-parameter associations. *Stat Med* **31**, 3821-3839, doi:10.1002/sim.5471 (2012).

3 Sera, F., Armstrong, B., Blangiardo, M. & Gasparrini, A. An extended mixed-effects framework for meta-analysis. *Stat Med* **38**, 5429-5444, doi:10.1002/sim.8362 (2019).
