# Supplementary material for: Impact of population aging on future temperature-related mortality at different global warming levels
Source: Nat Commun. 2024 Feb 27;15:1796. doi: 10.1038/s41467-024-45901-z (PMC10899213; doi:10.1038/s41467-024-45901-z)
Supplement: Supplementary file 2 — Reporting Summary [file 41467_2024_45901_MOESM2_ESM.pdf]

nature portfolio

Corresponding author(s): Kai Chen

Last updated by author(s): Dec 5, 2023

Reporting Summary

Nature Portfolio wishes to improve the reproducibility of the work that we publish. This form provides structure for consistency and transparency in reporting. For further information on Nature Portfolio policies, see our Editorial Policies and the Editorial Policy Checklist.

Statistics

For all statistical analyses, confirm that the following items are present in the figure legend, table legend, main text, or Methods section.

n/a	Confirmed	
☐	☒	The exact sample size (*n*) for each experimental group/condition, given as a discrete number and unit of measurement
☐	☒	A statement on whether measurements were taken from distinct samples or whether the same sample was measured repeatedly
☒	☐	The statistical test(s) used AND whether they are one- or two-sided<br>*Only common tests should be described solely by name; describe more complex techniques in the Methods section.*
☐	☒	A description of all covariates tested
☒	☐	A description of any assumptions or corrections, such as tests of normality and adjustment for multiple comparisons
☐	☒	A full description of the statistical parameters including central tendency (e.g. means) or other basic estimates (e.g. regression coefficient) AND variation (e.g. standard deviation) or associated estimates of uncertainty (e.g. confidence intervals)
☐	☒	For null hypothesis testing, the test statistic (e.g. *F*, *t*, *r*) with confidence intervals, effect sizes, degrees of freedom and *P* value noted<br>*Give P values as exact values whenever suitable.*
☒	☐	For Bayesian analysis, information on the choice of priors and Markov chain Monte Carlo settings
☒	☐	For hierarchical and complex designs, identification of the appropriate level for tests and full reporting of outcomes
☐	☒	Estimates of effect sizes (e.g. Cohen's *d*, Pearson's *r*), indicating how they were calculated

Our web collection on statistics for biologists contains articles on many of the points above.

Software and code

Policy information about availability of computer code

Data collection	No software was used in data collection.
Data analysis	R version 4.2.3; A sample of the R code analysis is available from https://github.com/CHENlab-Yale/MCC_ProjAging_Temp.

For manuscripts utilizing custom algorithms or software that are central to the research but not yet described in published literature, software must be made available to editors and reviewers. We strongly encourage code deposition in a community repository (e.g. GitHub). See the Nature Portfolio guidelines for submitting code & software for further information.

Data

Policy information about availability of data

All manuscripts must include a data availability statement. This statement should provide the following information, where applicable:

- Accession codes, unique identifiers, or web links for publicly available datasets
- A description of any restrictions on data availability
- For clinical datasets or third party data, please ensure that the statement adheres to our policy

All data needed to evaluate the conclusions in the paper are present in the paper and/or the Supplementary Materials. Data were collected within the MCC Collaborative Research Network under a data sharing agreement and cannot be made publicly available.

Research involving human participants, their data, or biological material

Policy information about studies with human participants or human data. See also policy information about sex, gender (identity/presentation), and sexual orientation and race, ethnicity and racism.

Reporting on sex and gender	We did not report the results by sex or gender.
Reporting on race, ethnicity, or other socially relevant groupings	We did not report the results by race, ethnicity, or other socially relevant groupings.
Population characteristics	The population in the exposure-response function analysis is decedents in 800 locations from 50 countries/areas.
Recruitment	No recruitment process was involved. We obtained the daily counts of deaths due to non-external causes (International Classification of Diseases, 9th Revision codes 0-799 or 10th Revision codes A00-R99) or all-cause deaths from the health departments in each studied country/area.
Ethics oversight	Using anonymized daily city-level mortality records, this study was Not Human Subject research.

Note that full information on the approval of the study protocol must also be provided in the manuscript.

Field-specific reporting

Please select the one below that is the best fit for your research. If you are not sure, read the appropriate sections before making your selection.

☒ Life sciences ☐ Behavioural & social sciences ☐ Ecological, evolutionary & environmental sciences

For a reference copy of the document with all sections, see nature.com/documents/nr-reporting-summary-flat.pdf

Life sciences study design

All studies must disclose on these points even when the disclosure is negative.

Sample size	All deaths in 800 locations from 50 countries/areas during 1995-2014 (historical) and projected deaths at 1.5, 2, and 3 °C of global warming.
Data exclusions	Data records with missing data for mortality or temperature records were excluded.
Replication	This is an observational study using data on daily mortality, air temperature, and climate projections data, and could be replicated using publicly available code.
Randomization	There was no experimental design or group allocation involved in this study.
Blinding	There was no experimental design or group allocation involved in this study.

Reporting for specific materials, systems and methods

We require information from authors about some types of materials, experimental systems and methods used in many studies. Here, indicate whether each material, system or method listed is relevant to your study. If you are not sure if a list item applies to your research, read the appropriate section before selecting a response.

Materials & experimental systems

n/a	Involved in the study	
☒	☐	Antibodies
☒	☐	Eukaryotic cell lines
☒	☐	Palaeontology and archaeology
☒	☐	Animals and other organisms
☒	☐	Clinical data
☒	☐	Dual use research of concern
☒	☐	Plants

Methods

n/a	Involved in the study	
☒	☐	ChIP-seq
☒	☐	Flow cytometry
☒	☐	MRI-based neuroimaging

Plants

Seed stocks	No applicable.
Novel plant genotypes	No applicable.
Authentication	No applicable.
